# Decoding non-canonical mRNA decay by the endoplasmic-reticulum stress sensor IRE1α

**DOI:** 10.1038/s41467-021-27597-7

**Published:** 2021-12-15

**Authors:** Adrien Le Thomas, Elena Ferri, Scot Marsters, Jonathan M. Harnoss, David A. Lawrence, Iratxe Zuazo-Gaztelu, Zora Modrusan, Sara Chan, Margaret Solon, Cécile Chalouni, Weihan Li, Hartmut Koeppen, Joachim Rudolph, Weiru Wang, Thomas D. Wu, Peter Walter, Avi Ashkenazi

**Affiliations:** 1 Department of Cancer Immunology, Genentech, Inc., 1 DNA Way, South San Francisco, CA 94080 USA; 2 Department of Structural Biology, Genentech, Inc., 1 DNA Way, South San Francisco, CA 94080 USA; 3 Department of Discovery Chemistry, Genentech, Inc., 1 DNA Way, South San Francisco, CA 94080 USA; 4 Department of Microchemistry, Proteomics and Lipidomics, Genentech, Inc., 1 DNA Way, South San Francisco, CA 94080 USA; 5 Department of Pathology, Genentech, Inc., 1 DNA Way, South San Francisco, CA 94080 USA; 6 Howard Hughes Medical Institute, University of California, San Francisco, CA 94143 USA; 7 University of California, San Francisco, CA 94143 USA; 8 Department of Oncology Bioinformatics Genentech, Inc., 1 DNA Way, South San Francisco, CA 94080 USA

**Keywords:** RNA, Cancer models, Endoplasmic reticulum

## Abstract

Inositol requiring enzyme 1 (IRE1) mitigates endoplasmic-reticulum (ER) stress by orchestrating the unfolded-protein response (UPR). IRE1 spans the ER membrane, and signals through a cytosolic kinase-endoribonuclease module. The endoribonuclease generates the transcription factor XBP1s by intron excision between similar RNA stem-loop endomotifs, and depletes select cellular mRNAs through regulated IRE1-dependent decay (RIDD). Paradoxically, in mammals RIDD seems to target only mRNAs with XBP1-like endomotifs, while in flies RIDD exhibits little sequence restriction. By comparing nascent and total IRE1α-controlled mRNAs in human cells, we identify not only canonical endomotif-containing RIDD substrates, but also targets without such motifs—degraded by a process we coin RIDDLE, for RIDD lacking endomotif. IRE1α displays two basic endoribonuclease modalities: highly specific, endomotif-directed cleavage, minimally requiring dimers; and more promiscuous, endomotif-independent processing, requiring phospho-oligomers. An oligomer-deficient IRE1α mutant fails to support RIDDLE in vitro and in cells. Our results advance current mechanistic understanding of the UPR.

## Abstract (web summary)

IRE1 helps mitigate endoplasmic-reticulum stress by cleaving specific mRNAs at a conserved sequence endomotif via regulated IRE1-dependent decay (RIDD). Here the authors discover a more promiscuous IRE1 activity dubbed RIDD lacking endomotif (RIDDLE).

## Introduction

The endoplasmic-reticulum (ER) mediates folding of newly synthesized secretory and membrane proteins. Excess folding demand leads to ER accumulation of misfolded proteins, causing ER stress. This engages an intracellular signaling network, dubbed the unfolded-protein response (UPR), which aims to reestablish homeostasis^1–3^. The mammalian UPR entails three ER-transmembrane proteins: IRE1α, PERK, and ATF6, which coordinate adaptive changes to expand ER capacity while abating ER load^1,3,4^. If adaptation fails, the UPR triggers apoptotic cell death^4–6^. UPR dysregulation contributes to several diseases^7–11^. Cancer cells often leverage the UPR, including IRE1α, to circumvent ER stress and maintain malignant growth^10,12–17^. Better mechanistic understanding of the UPR would help elucidate its role in disease, and advance its potential for medical translation.

IRE1α comprises ER-lumenal and transmembrane domains, and a cytosolic kinase-endoribonuclease (KR) module^18,19^. Unfolded-protein sensing by the lumenal domain drives IRE1α homo-oligomerization, kinase-mediated *trans*-autophosphorylation, and endoribonuclease activation^19–24^. The RNase produces the transcription factor spliced X-box binding protein 1 (XBP1s), and depletes multiple cellular mRNAs through a process called regulated IRE1-dependent decay (RIDD)^25–27^. XBP1s-target genes support protein folding and ER-associated degradation (ERAD)^28^. IRE1α cleaves unspliced XBP1u mRNA at two similar stem-loop endomotifs, removing a 26-nt intron^29–31^. In turn, RtcB ligates the severed exons, generating XBP1s^32–34^. Cleavage of XBP1u at each splice site requires an energetically stable stem, as well as a 7-nt consensus sequence CNG|CAGN within the loop, with scission between G and C in the third and fourth positions^34,35^.

RIDD remains puzzling^27,36^. In the budding yeast *S. cerevisiae*, IRE1 triggers non-conventional mRNA splicing of the *XBP1* ortholog *HAC1*, yet lacks RIDD activity^26^. Conversely, in the fission yeast *S. pombe*, IRE1 performs RIDD, which targets a UG|CU core motif within variably sized stem-loop structures, but *HAC1* mRNA is absent^37^. In the fruit fly *D. melanogaster*, RIDD primarily targets ER-bound mRNAs, with minimal sequence and unknown structure restriction^26,38,39^. By contrast, in mammals, cleavage of known RIDD mRNA substrates seems to require an XBP1u-like consensus loop sequence CNG|CAGN, enclosed by a stable stem^40–43^. Mammalian RIDD regulates several additional cellular functions besides abating ER load, including triglyceride and cholesterol metabolism^44^; apoptosis signaling through DR5^45–47^; protective autophagy via BLOC1S1 (BLOS1)^48^; and DNA repair through Ruvbl1^49,50^. A canonical stem-loop endomotif is necessary but not sufficient to predict mammalian RIDD, while translational stalling can enhance mRNA depletion^41^. Other mechanisms, such as NO-GO decay and the cytosolic exosome, have been implicated in completing degradation after endomotif-directed mRNA cleavage by IRE1^51^. However, it is unknown whether IRE1 itself can conduct full RNA digestion. Autophosphorylation supports IRE1 oligomerization^22,45,50,52^ and may affect RNase output^21,22,53–57^. The specific requirements for endomotif-restricted *vs*. non-restricted IRE1 RNase activity remain ill-defined, and the significance of the latter in cells of higher metazoans remains elusive.

To investigate the scope of RIDD in a human cell line, we took a dual next-generation RNA sequencing approach that distinguishes total cellular mRNAs from nascent transcripts. Surprisingly, in ER-stressed cells, human IRE1α depletes not only canonical endomotif-containing RIDD substrates, but also multiple mRNAs not possessing such consensus sequences, identified as targets of a process we dub “RIDD lacking endomotif” (RIDDLE). By isolating homotypic complexes of the human IRE1α kinase-endoribonuclease (IRE1-KR) module in monomeric, dimeric, or oligomeric form, we demonstrate that RIDD and RIDDLE reside in two distinct RNase modalities: endomotif-directed cleavage, minimally requiring IRE1α dimers; and endomotif-independent cleavage, necessitating phospho-oligomers. Indeed, an IRE1α mutation that specifically disrupts oligomerization permits endomotif-directed cleavage yet blocks RIDDLE both in vitro and in cells.

## Results

### Integrating RNAseq and GROseq to identify potential mRNA targets of IRE1-dependent decay

We reasoned that subtracting nascent-transcript changes from global alterations in mRNA abundance would help distinguish mRNA decay from diminished transcription. Accordingly, to uncover mRNAs subject to IRE1-dependent decay, we applied two parallel RNA sequencing approaches: (1) classical RNAseq, which interrogates the steady-state transcriptome; and (2) global nuclear run-on sequencing (GROseq)^58^, which probes the nascent transcriptome. We determined dependency on IRE1α and ER stress by treating human MDA-MB-231 breast cancer cells, harboring homozygous wildtype (WT) or CRISPR/Cas9 knockout (KO) *IRE1*α alleles^14^, with the classical ER stressor, Thapsigargin (Tg).

**Fig. 1:**
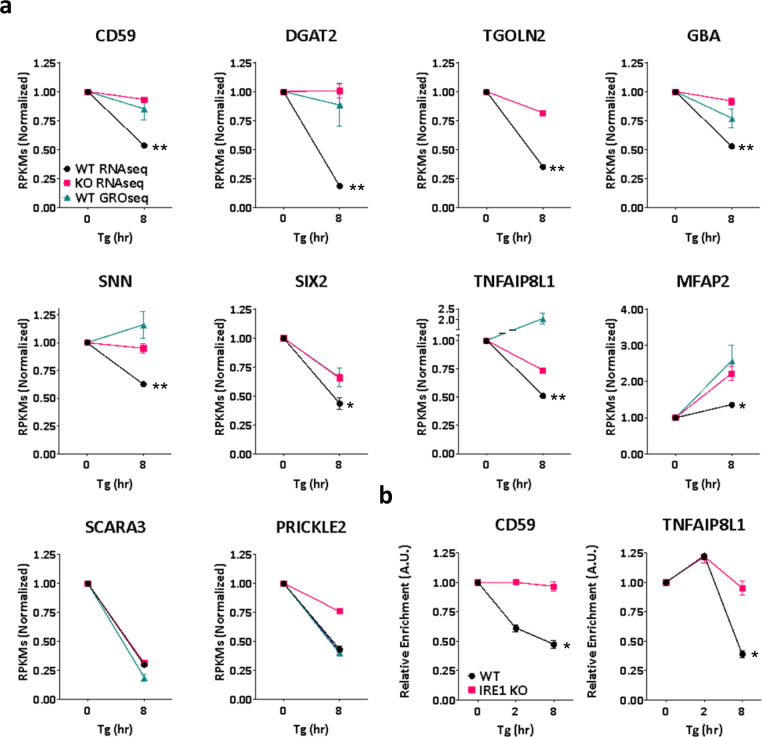
Integrative RNAseq and GROseq analyses identify human RIDD and RIDDLE targets. **a** Mean RPKM values for various examples of IRE1α RNase targets from the RNAseq and GROseq datasets in WT and IRE1α KO MDA-MB-231 cells before and after ER-stress induction by Tg (100 nM). Note that the GROseq data points in some cases are hidden behind the RNAseq data points. *n* = 3 biologically independent experiments. Data are presented as mean values ± SEM. An Unpaired *t*-test was used to calculate *p*-values. **b** Kinetic RT-qPCRs analysis of CD59 and TNFAIP8L1 transcripts in IRE1α WT and KO MDA-MB-231 cells, before and after ER-stress induction by Tg (100 nM) for 2 and 8 h. *n* = 3 biologically independent experiments. Data are presented as mean values ± SEM. An Unpaired *t*-test was used to calculate *p*-values. *P ≤ 0.05; **P ≤ 0.01.

Most global-transcript levels did not change under these conditions (Supplementary Fig. 1a). We identified 54 mRNAs as potential RIDD substrates: these displayed an IRE1α-dependent, ER stress-induced decrease in abundance of at least 1.4-fold as measured by RNAseq, without a corresponding decline in transcription as measured by GROseq (Supplementary Table 1). Of note, these data do not distinguish between direct IRE1α-mediated decay and indirect IRE1α-triggered RNA depletion. We graphically illustrate the regulation of eight of these mRNAs, including the known RIDD targets CD59 and DGAT2, as well as several novel ones, namely, TGOLN2, GBA, SNN, SIX2, TNFAIP8L1, and MFAP2 (Fig. 1a). By contrast, other mRNAs showed more complex behaviors: SCARA3 exemplifies a transcript that is downregulated independently of IRE1α altogether, consistent with other results^25,41^. Furthermore, PRICKLE2 represents an mRNA that is transcriptionally downregulated in an IRE1α-dependent manner. Moreover, MFAP2 exemplifies an mRNA that is upregulated by ER stress while being suppressed via IRE1α. We further confirmed IRE1α-dependent modulation of several of these transcripts by kinetic RT-qPCR analysis. Individual mRNAs displayed different decay rates, with some—such as TNFAIP8L1, SNN, and GBA—showing a lag in depletion after Tg addition (Fig. 1b and Supplementary Fig. 1b). We also verified IRE1α-dependent depletion of select transcripts in response to a different ER stress inducer, i.e., Tunicamycin (Tm), and/or in other cell lines, namely, U2OS and HCT116 (Supplementary Fig. 1c–e). Furthermore, immunoblot analyses indicated appreciable IRE1α-dependent downregulation of proteins encoded by several of the above mRNAs in Tg-treated MDA-MB-231 cells (Supplementary Fig. 1f); or in AMO1 and KMS27 human multiple myeloma cells displaying constitutive phospho-IRE1α and XBP1s (Supplementary Fig. 1g, h).

Bioinformatic analysis of the integrated RNAseq and GROseq data suggested a notable frequency of hits in annotated categories of cell death or survival, cell signaling, post-translational modification, cell morphology, and cell cycle (Supplementary Table 2). Many targets lacked a signal peptide or anchor (Supplementary Table 1), consistent with modulation of diverse cellular functions^45–47^.

As expected, GROseq detected over 300 mRNAs that displayed IRE1α-dependent transcriptional upregulation by ER stress, enriched in Gene Ontology categories of ER stress, ER-to-Golgi vesicle transport, IRE1-mediated UPR, N-linked glycosylation, ERAD (Endoplasmic-Reticulum-Associated protein Degradation), and others (Supplementary Table 3). Several mRNAs represented known XBP1s transcriptional targets (Supplementary Fig. 1i). The GROseq data also covered the genomic region encoding the XBP1u intron (Supplementary Fig. 1j), providing further methodological validation.

### IRE1α displays two distinct endoribonuclease modalities

**Fig. 2:**
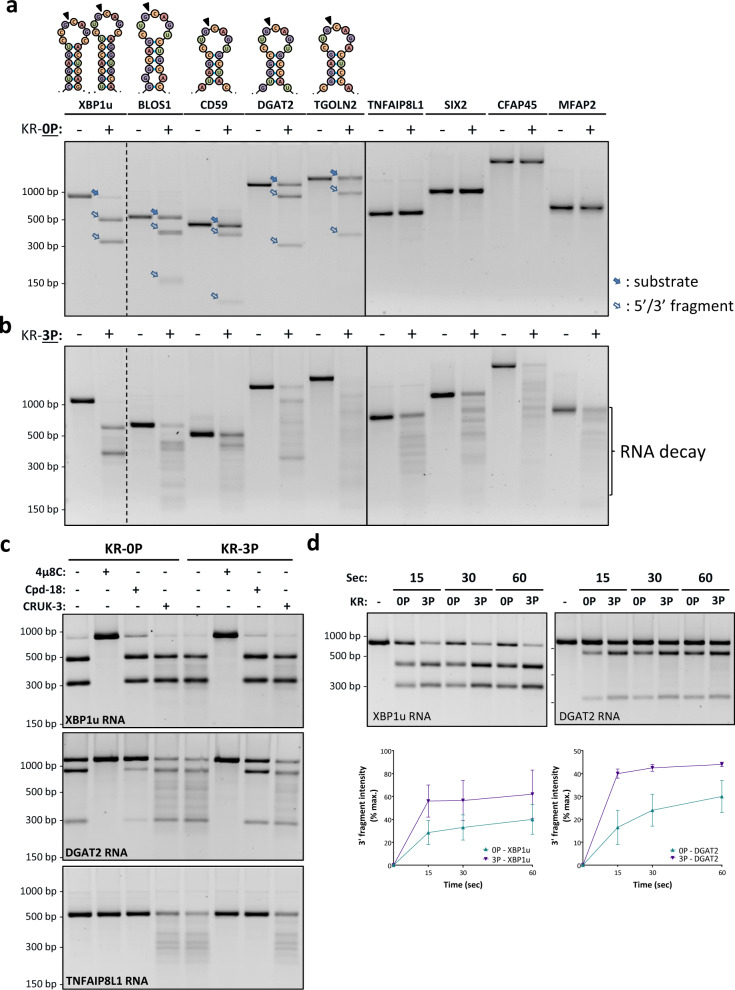
Phosphorylation state of IRE1α-KR affects RNase modality. In vitro-generated T7-RNAs were incubated with purified recombinant IRE1α protein (residues G547-L977) comprising the kinase-endoribonuclease module (IRE1-KR), in non-phosphorylated (KR-0P) (**a**) or fully phosphorylated (KR-3P) (**b**) form, and RNA products were analyzed by agarose gel electrophoresis. Where shown, solid arrows indicate RNA substrates; open arrows mark RNA cleavage products, representing the 5′ and 3′ fragments around the cleavage site. Specific RNA endomotifs are depicted on top for respective targets. **c** IRE1-KR-0P and IRE1-KR-3P digestions of XBP1, DGAT2, and TNFAIP8L1 transcripts in the presence of the IRE1α RNase inhibitor 4μ8C, the IRE1α kinase-based RNase inhibitor Compound 18 (Cpd-18), or the IRE1α kinase-based RNase activator Compound 3 (CRUK-3), all at 5 µM. **d** IRE1-KR-3P digestion of XBP1u and DGAT2 mRNA at a shorter duration. Intensities of the 3′ RNA fragment were quantified for XBP1u and DGAT2 using GelQuantNET from the RNA digestion agarose gels shown above. *n* = 2 biologically independent samples. Data are presented as mean values ± SEM.

To explore the molecular features that might govern IRE1’s RNase modality, we purified recombinant human IRE1-KR proteins in unphosphorylated (0P) or fully phosphorylated (3P) states (Supplementary Fig. 2a). IRE1-KR-0P efficiently cleaved an XBP1u-based T7 RNA polymerase transcript, at both of the known stem-loop endomotifs: processing produced ~500-nt and ~350-nt fragments, corresponding to the 5′ and 3′ exons; and the 26-nt intron (Fig. 2a and Supplementary Fig. 2b–d). Scrambling either loop sequence prevented cleavage, while inserting 43-nt or 50-nt random sequences between the splice sites proportionally shifted the resulting bands (Supplementary Fig. 2b, d). Thus, IRE1-KR-0P performs precise cleavage of XBP1u RNA at the two consensus sites, in keeping with other data^22,29,31^.

Next, we examined the capacity of IRE1-KR-0P to process 8 of the 54 potential RIDD targets identified above, including 3 established and 5 newly uncovered ones (Fig. 2a). We chose these particular mRNAs because their length was suitable for direct in vitro transcription and observation by agarose gel electrophoresis. IRE1-KR-0P performed single-site cleavage of four of the RNAs, encoding BLOS1, CD59, DGAT2, and TGOLN2. Each of these contains an XBP1-like stem-loop endomotif, having the core consensus sequence CNGCAGN within a projected stem-loop secondary structure; the 5′ and 3′ fragments produced by IRE1-KR-0P for each RNA agreed in size with the location of the stem-loop endomotif (Fig. 2a and Supplementary Table 4). Compared to XBP1u RNA, processing of the latter transcripts left more RNA substrate intact at the timepoint analyzed, indicating generally slower or less efficient reactions. Scrambling the loop sequence of CD59 and DGAT2 prevented cleavage by IRE1-KR-0P (Supplementary Fig. 2e), confirming its endomotif-restricted endoribonuclease activity.

Surprisingly, under the same reaction conditions, IRE1-KR-0P failed to cleave the other 4 RNAs, encoding TNFAIP8L1, SIX2, CFAP45, and MFAP2 (Fig. 2a). Resistance of these RNAs to processing correlated with their lack of a robust canonical stem-loop endomotif, as described below.

IRE1-KR-3P produced the same XBP1u RNA cleavage fragments as did IRE1-KR-0P; however, IRE1-KR-3P distinctly generated additional XBP1u fragments, visible as a faint smear (Fig. 2b and Supplementary Fig. 2c), suggesting that it can catalyze further RNA decay. Uniquely, IRE1-KR-3P also cleaved into multiple fragments each of the 8 RNA substrates, including those that resisted cleavage by IRE1-KR-0P. Since this promiscuous processing occurs outside the endomotif, we categorize it as RIDDLE activity.

To ascertain dependence on IRE1α, we included the IRE1α RNase-directed inhibitor, 4μ8c^59^, which completely prevented RNA cleavage by both IRE1-KR-0P and IRE1-KR-3P (Fig. 2c). In contrast, an IRE1α kinase-directed RNase inhibitor, Compound 18^15,60^, blocked RNA decay, but not endomotif cleavage; and a kinase-based IRE1α RNase activator, CRUK-3^61^, endowed IRE1-KR-0P with an IRE1-KR-3P-like ability to cleave RNA in both modalities. Upon processing by IRE1-KR-3P, endomotif-mutated XBP1u and CD59 RNA substrates were more stable than WT counterparts (Supplementary Fig. 2f), suggesting that in vitro endomotif-based cleavage can prime these mRNAs for RIDDLE, which mediates further decay of the initial fragments. Kinetic analyses indicated faster endomotif cleavage by IRE1-KR-3P than IRE1-KR-0P, evident by swifter generation of 3′ products from substrate RNAs (Fig. 2d), and more rapid scission of a short synthetic hairpin endomotif (Supplementary Fig. 2g). These results demonstrate that IRE1α can switch between two different modalities: (1) endomotif-restricted activity, which mediates both XBP1u intron excision and canonical RIDD; (2) endomotif-independent activity, which mediates RIDDLE, including the further degradation of classical endomotif-containing substrates. IRE1-KR-0P can support the first modality but not the second, whereas IRE1-KR-3P conducts both.

Combining the data for the above 8 RNAs with empirical results for four additional validated targets, i.e., PIGQ, BMP4, BCAM, and SNN (Supplementary Fig. 2h), together with 29 earlier characterized human or mouse RIDD substrates^27,43^, we developed a computational algorithm, dubbed gRIDD, which accounts for all of these verified substrates, and determines the presence of any canonical stem-loop endomotifs for any given mRNA (see Supplemental Methods for a detailed description). This algorithm takes into account features that include: (1) conformity of the loop sequence to the consensus; (2) loop length and stem stability in the context of the 55–60 flanking bases; (3) number of paired bases and any unpaired bases in the stem. To validate gRIDD, we analyzed a test set of four additional transcripts from our screen. Two proved computationally to possess a canonical stem-loop endomotif, with either an exact match to or a single nucleotide variation from the consensus loop sequence (GBA and WT1, respectively); accordingly, both IRE1-KR-0P and IRE1-KR-3P should cleave these latter RNAs. Another test RNA (CCDC69) had a weak endomotif that failed gRIDD criteria, while a fourth one (AIM2) lacked an endomotif altogether; accordingly, only IRE1-KR-3P should cleave these latter RNAs. Supporting gRIDD’s accuracy, all test RNAs displayed the predicted cleavage characteristics (Supplementary Fig. 2i). Moreover, additional known RIDD targets, i.e., murine ANGPTL3^44^, murine SUMO2, human SUMO3^42^, and human DR5^47^, also met the algorithm’s criteria, whereas 10 mRNAs previously excluded from RIDD^41^ did not.

Of the 54 mRNAs we identified here (Supplementary Table 4), gRIDD mapped 30 transcripts as possessing a canonical stem-loop endomotif (RIDD), including 22 with an exact, and 8 with an acceptably variant, consensus loop sequence. In addition, gRIDD mapped 24 transcripts as lacking endomotif (RIDDLE), including 15 with one or more sub-par stem-loop sequences that fail the algorithm’s criteria, and another 9 that had no discernable endomotif. Our data empirically confirmed RNAs from each of these subclasses (Fig. 2a and Supplementary Fig. 2h, i).

To seek further validation of the RIDDLE modality, we examined whether IRE1α could access a RIDDLE substrate in ER-stressed cells. We first visualized the endogenous IRE1α protein in MDA-MB-231 cells by immunofluorescence with a sensitive and selective monoclonal antibody^14^. IRE1α displayed diffuse and multi-punctate staining in polarized juxtanuclear regions, consistent with ER localization, with no detectable staining in KO cells (Supplementary Fig. 2j). To co-visualize specific mRNAs, we performed in situ hybridization for the RIDDLE target, TNFAIP8L1, or the IRE1α-independent mRNA, PRICKLE2. Whereas IRE1α puncta showed readily measurable co-localization with TNFAIP8L1 mRNA, they displayed substantially less positional overlap with PRICKLE2 mRNA (Supplementary Fig. 2j, k). Thus, IRE1α protein can specifically access a RIDDLE mRNA substrate in ER-stressed cells.

### RIDDLE is more promiscuous in substrate recognition, yet non-random

**Fig. 3:**
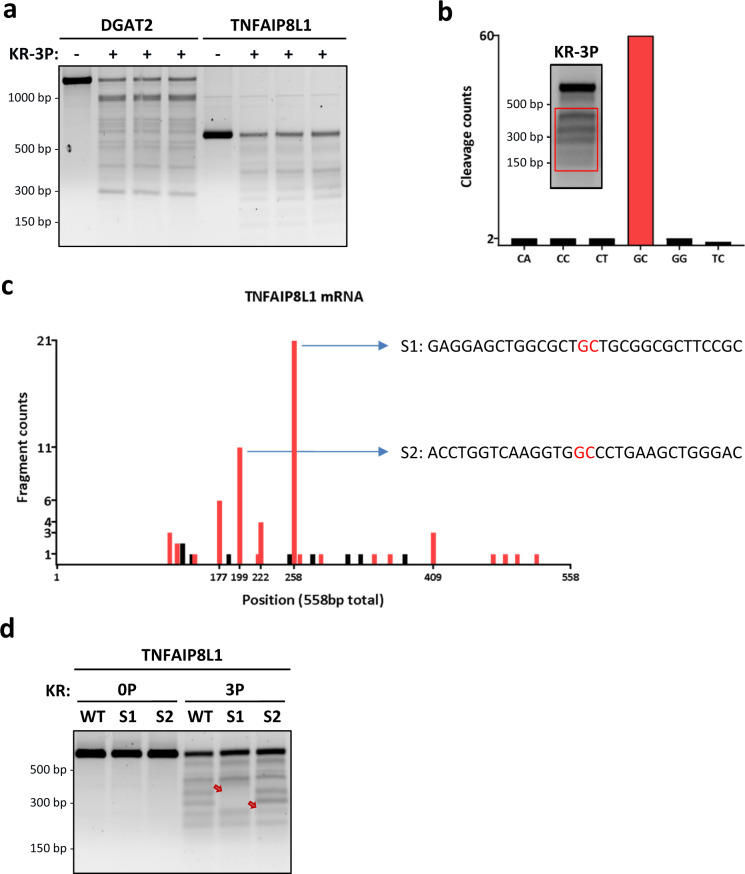
RIDDLE is more promiscuous in substrate recognition, yet non-random. **a** Comparative KR-3P digestion of DGAT2 and TNFAIP8L1 transcripts performed in three independent experiments. **b** Amount of RNA fragments sequenced whose 3′ end leads to the cleaved nt pair designated on the *x* axis. The first nt in the pair represents the last sequenced nt from the RNA fragment, while the second shows the subsequent base in the RNA sequence. Inset: red box indicates the portion of the gel that was extracted for Sanger sequencing. **c** Mapping of the last base pair (3′ end) from each individual RNA fragment sequenced within the TNFAIP8L1 mRNA. Red bars indicate cleavage sites between a GC nt pair. Black bars indicate non GC cleavage sites. **d** RNA digestions of WT TNFAIP8L1 and TNFAIP8L1 mutated at locations S1 and S2. The red arrows indicate a change in banding pattern as compared to WT.

To test the uniformity of RNA decay by IRE1-KR-3P, we performed three independent RNA digestions of DGAT2 and TNFAIP8L1. Strikingly, IRE1-KR-3P generated the same banding pattern in all three cases, indicating that—although the processing appeared more promiscuous than endomotif-directed cleavage—it entailed consistent, non-random fragmentation (Fig. 3a). To search for underlying sequence requirements, we subjected TNFAIP8L1 RNA to cleavage by IRE1-KR-3P, resolved the products by agarose gel electrophoresis, and extracted them for Sanger sequencing. Overall, 60 of the 69 reads thus obtained showed cleavage at GC sites, with infrequent processing at other positions (Fig. 3b). Alignment along the TNFAIP8L1 RNA indicated two relatively enriched cleavage locations, designated S1 (21/69) and S2 (11/69), both at GC sites (Fig. 3c, and Supplementary Data 1). The sequences surrounding these two sites did not meet gRIDD’s stem-loop endomotif criteria (Supplementary Table 5). Regardless, replacing GC by TA at S1 or S2 abolished or diminished the corresponding cleavage product (Fig. 3d), confirming site selectivity. Likewise, analysis of DGAT2 RNA also revealed a preponderance of GC cleavages (34/61 reads, excluding the endomotif), and mutation of the most prevalent site precluded the corresponding product (Supplementary Fig. 3a–c, Supplementary Data 1 and Supplementary Table 5). Thus, although RIDDLE is less restricted, it appears to favor GC sites.

To study RIDDLE further, we performed a kinetic analysis for cleavage of the endomotif-lacking AIM2 RNA (Supplementary Fig. 3d). Some AIM2 fragments decreased in abundance over time whereas others persisted or even accumulated, suggesting that RIDDLE entails mainly endoribonuclease activity, though exoribonuclease activity against some of the initial products cannot be ruled out. In addition, we treated MDA-MB-231 cells with the previously characterized kinase-directed cellular IRE1α activator, G-9807^62^. This compound induced upregulation of XBP1s, as well as downregulation of the RIDD substrate DGAT2 and the RIDDLE targets TNFAIP8L1 and SIX2 (Supplementary Fig. 3e). Thus, direct kinase-based activation of cellular IRE1α produces a similar set of RNase modalities as does indirect activation by classical ER stressors.

### Phospho-oligomeric state governs IRE1α’s endoribonuclease modality

**Fig. 4:**
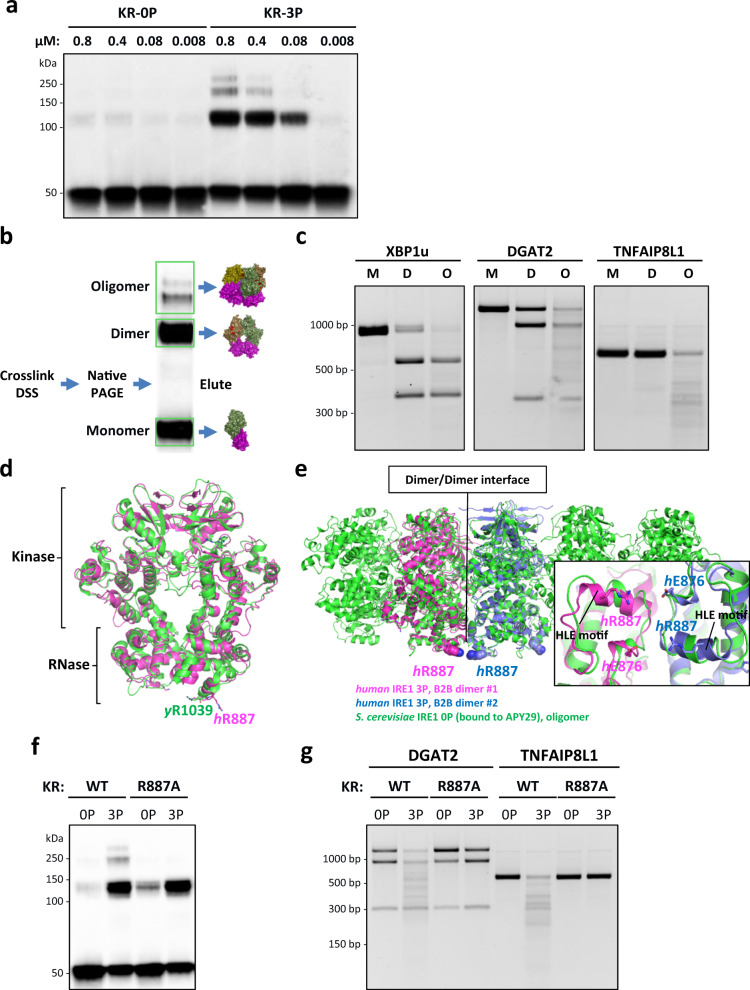
Phospho-oligomeric state governs IRE1α‘s RNase modality. **a** DSS crosslinking of IRE1-KR-0P and IRE1-KR-3P at various concentrations. (All lanes have the same final amount of protein loaded.) **b** Cartoon depicting the procedure to extract KR-3P fractions before incubation with T7 RNAs. **c** Digestion of XBP1u, DGAT2, and TNFAIP8L1 RNA by isolated fractions of IRE1-KR-3P: M, monomer; D, dimer; and O, oligomer. **d** WT structure of IRE1α dimer with the residues of interest highlighted: alignment of yeast B2B IRE1α (PDB ID: 3FBV, green cartoon) and human B2B IRE1α (PDB ID: 6W3C, magenta cartoon). **e** Alignment of yeast oligomeric IRE1α (PDB ID: 3FBV, green cartoon) and two dimers of human IRE1α (PDB ID: 6W3C, magenta and blue cartoons). Inset: R887-E876 spatial arrangement based on the alignment. **f** DSS crosslinking of purified recombinant IRE1-KR-3P WT and R887A. **g** Digestion of DGAT2 and TNFAIP8L1 RNA by IRE1-KR-3P WT and R887A.

While activation of the IRE1α RNase minimally requires homodimerization, the importance of higher-order assembly is unclear^56,63^. To investigate the latter, we covalently tethered IRE1-KR protomers by chemical crosslinking and studied their RNase mode. Immunoblot analysis revealed that IRE1-KR-0P was primarily monomeric yet formed some detectable dimers in a concentration-dependent manner; in contrast, IRE1-KR-3P assembled not only more prominent, concentration-dependent dimers, but also oligomers displaying relative masses consistent with potential tetramers and hexamers (Fig. 4a). These results agree with other evidence that phosphorylation of IRE1α coincides with dimerization and higher-order oligomerization^22,52,62,64^.

IRE1-KR-3P retained RNase activity after crosslinking, evident by cleavage of XBP1u, DGAT2, and TNFAIP8L1 RNAs (Supplementary Fig. 4a). To examine RNase modality, we used native polyacrylamide gel electrophoresis to resolve crosslinked IRE1-KR-3P complexes into monomers, dimers, and oligomers (Fig. 4b). We excised and eluted each complex from the gel, and reacted it with RNA substrates in solution (Fig. 4c). Whereas IRE1-KR-3P monomers were inactive as expected, dimers processed the XBP1u RNA at both intron excision sites, and cleaved the DGAT2 RNA endomotif; however, they failed to further degrade DGAT2 RNA appreciably, nor did they cleave TNFAIP8L1 RNA. In contrast, IRE1-KR-3P oligomers cleaved XBP1u more efficiently, while substantially degrading both DGAT2 and TNFAIP8L1 RNAs. Upon prolonged incubation, IRE1-KR-3P oligomers achieved complete RNA degradation, as evident for DGAT2 RNA (Supplementary Fig. 4b). Thus, whereas endomotif-directed cleavage at minimum requires dimers, RIDDLE necessitates higher-order oligomerization. Supporting this conclusion, Compound 18, which we found earlier to block endomotif-independent but not endomotif-directed cleavage by IRE1-KR-3P (Fig. 2c), fully inhibited IRE1-KR-3P oligomerization, while incompletely attenuating dimerization (Supplementary Fig. 4c). Moreover, CRUK-3, which enhanced both endomotif-directed cleavage and endomotif-independent degradation by IRE1-KR-0P (Fig. 2c), congruently augmented IRE1-KR-0P dimerization and oligomerization (Supplementary Fig. 4c). Mutational analysis confirmed that at least two of the three phosphorylation sites (serine 724, 726, 729) within the kinase activation loop of IRE1α were important for enhanced dimer formation, higher-order oligomerization, and the corresponding RNase modality (Supplementary Fig. 4d). The triply phosphorylated protein showed a markedly better capacity to dimerize and oligomerize and to perform RNA decay. To address more specifically the importance of phosphorylation for RIDDLE, we tested DGAT2 processing by increasing concentrations of non-phosphorylated IRE1-KR-0P protein (Supplementary Fig. 4e). Despite progressively oligomerizing at higher concentrations, IRE1-KR-0P remained restricted to endomotif-directed processing and failed to perform RIDDLE even at 12 µM. In contrast, IRE1-KR-3P formed comparable amounts of higher-order oligomers at a much lower concentration of 0.8 µM, and carried out both endomotif-directed cleavage and RIDDLE. These data suggest that IRE1α phosphorylation regulates RIDDLE activity beyond a direct support of dimerization and oligomerization.

To obtain further mechanistic insight, we screened for human IRE1α mutations that might disrupt oligomerization, by testing several amino acid positions previously studied within yeast IRE1^63,64^. One variant—R887A—proved particularly useful. Arginine 887 resides in the RNase domain within a helix loop element (HLE), shown to be important for binding and cleavage of *HAC1* mRNA in *S. cerevisiae*^64^. As such, R887 does not stabilize IRE1’s so-called back-to-back (B2B) dimer interface (Fig. 4d). However, structural overlay of human B2B dimers (PDB ID: 6W3C) onto oligomeric *S. cerevisiae* IRE1 (PDB ID: 3FBV) places R887 at the interface of two B2B dimers within the projected human oligomer (Fig. 4e). Importantly, although IRE1-KR-R887A dimerized, it failed to form higher-order oligomers, regardless of phosphorylation (Fig. 4f). Compared to WT IRE1-KR-0P, unphosphorylated R887A performed endomotif-directed cleavage of DGAT2 and did not appreciably degrade DGAT2 or TNFAIP8L1 RNAs (Fig. 4g). Strikingly, phosphorylated IRE1-KR-R887A retained endomotif-directed DGAT2 cleavage, but unlike WT IRE1-KR-3P, it failed to degrade DGAT2 and TNFAIP8L1 RNAs. These loss-of-function results strongly validate the conclusion that RIDDLE depends on higher-order phospho-oligomers of IRE1α.

### R887A mutant IRE1α displays cellular deficiency in oligomerization and RIDDLE

**Fig. 5:**
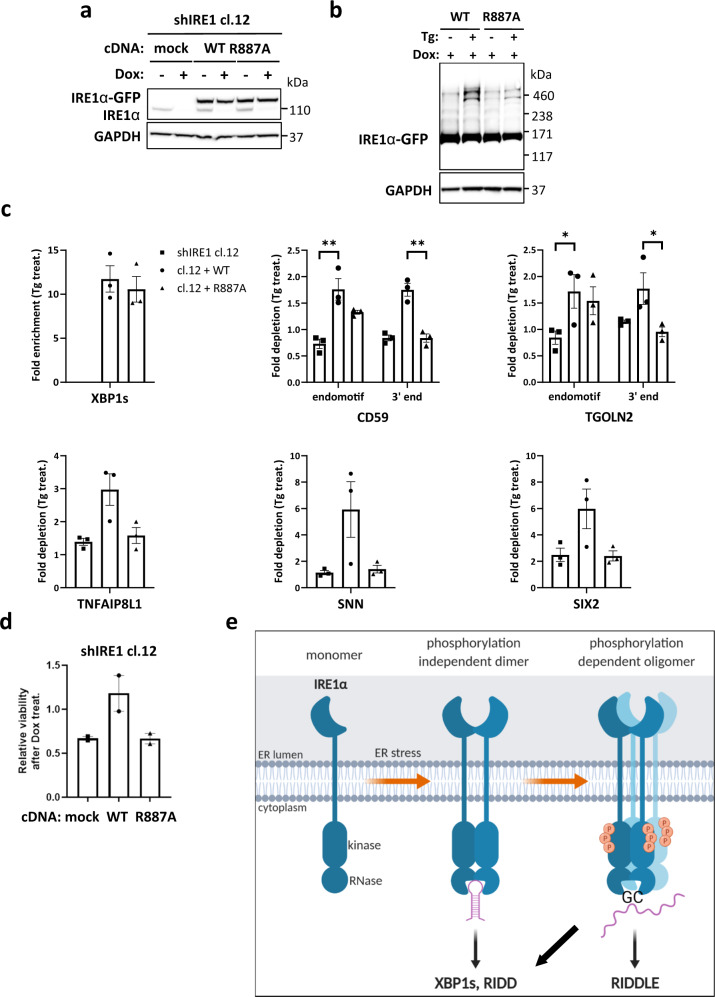
R887A mutant IRE1α displays cellular deficiency in oligomerization and RIDDLE. **a** Western blot analysis of endogenous and ectopic IRE1α variant expression in MDA-MB-231 cells harboring Dox-inducible IRE1α shRNA stably transfected with transgenic WT or R887A mutant versions of IRE1α-GFP. **b** Immunoblot analysis of MDA-MB-231 cells after treatment with Tg (100 nM, 4 h) followed by DSS crosslinking. Left panel shows parental shIRE1α cl.12 cell line. Right panel shows IRE1α WT and R887A rescues of Doxycycline-treated cl.12 cells with endogenous IRE1α knockdown. **c** RT-qPCR analysis of IRE1α RNase targets CD59, TGOLN2 (RIDD), and TNFAIP8L1, SNN, and SIX2 (RIDDLE). Ct values for XBP1 in sample shIRE1 cl.1 prior to Tg treatment were >34, precluding ratio calculations and were therefore not plotted. *n* = 3 biologically independent experiments. Data are presented as mean values ± SEM. A 2-way ANOVA test was used to calculate p-values for CD59 and TGOLN2, and an unpaired *t*-test for the remaining targets. **d** Analysis of cell viability by Cell-Titer Glo after Dox treatment for 7 days on Ultra-Low Attachment (ULA) plates. *n* = 2 biologically independent experiments. Data are presented as mean values ± SEM. **e** Model depicting IRE1α’s principal modes of endoribonuclease function and their underlying phospho-oligomeric states during ER stress. *P ≤ 0.05; **P ≤ 0.01.

To extend the principles gleaned from our studies of IRE1-KR in vitro to full-length IRE1α in cells, we devised a functional complementation strategy: we stably transfected shRNA-resistant cDNA expression plasmids encoding GFP-tagged^56^ WT or IRE1α-R887A into MDA-MB-231 cells harboring doxycycline (Dox)-inducible shRNAs against IRE1α, and isolated GFP-positive transfectants by cell sorting. As expected, each ectopic protein was expressed independent of Dox-inducible depletion of endogenous IRE1α (Fig. 5a). The transgenic proteins migrated at a higher molecular mass, consistent with their GFP tagging, and showed elevated expression relative to endogenous IRE1α. Crosslinking analysis of reconstituted cells confirmed that R887A was markedly deficient in oligomer formation as compared to IRE1α-WT (Fig. 5b). RT-qPCR analysis after a 72-h Dox-induced depletion of intrinsic IRE1α demonstrated that the ectopic WT and mutant variants supported a comparable fold-induction of XBP1s upon ER stress (Fig. 5c), suggesting similar capacity for endomotif-directed cleavage. To monitor RNase modality toward RIDD targets, we designed two specific RT-qPCR primer pairs for CD59 or TGOLN2: One encompasses the endomotif and therefore measures endomotif-directed cleavage (RIDD); the other covers the mRNA’s 3′ end and hence detects decay (RIDDLE; schematized in Supplementary Fig. 5a). Although both WT and R887A mediated endomotif-directed cleavage of both CD59 and TGOLN2, only IRE1α-WT enabled 3′-end depletion, i.e., RIDDLE (Fig. 5c). Moreover, R887A also appeared inferior to IRE1α-WT in mediating 3′-end depletion of mRNAs encoding TNFAIP8L1, SNN, and SIX2 (Fig. 5c), as well as GBA and BCAM (Supplementary Fig. 5b); yet it performed endomotif-directed cleavage of DGAT2 similar to IRE1α-WT (Supplementary Fig. 5b). Kinetic analysis of CD59 depletion under ER stress indicated similar rates for endomotif and 3′-end depletion (Supplementary Fig. 5c), suggesting close temporal association of the two RNase modalities in this context. To examine an additional cell line, we applied a similar complementation strategy to HCC1806 cells harboring shRNA-based knockdown of endogenous IRE1α with ectopic expression of IRE1α-WT or R887A, which produced similar results (Supplementary Fig. 5d, e). These data support the same two basic IRE1α RNAse modalities in vitro as well as in cells.

The failure of IRE1α-R887A to oligomerize and to acquire the RIDDLE modality afforded an opportunity to explore the functional consequences of this mutation. To this end, we leveraged earlier work demonstrating that certain cancer cell lines depend on IRE1α for viability during 3D growth^14^. As expected, in both MDA-MB-231 and HCC1806 cells, transgenic IRE1α-WT rescued the loss of viability conferred by Dox-inducible knockdown of endogenous IRE1α; in contrast, IRE1α-R887A failed comparably to restore cell viability (Fig. 5d and Supplementary Fig. 5e–h). These results suggest a linkage between IRE1α’s capacity to oligomerize, perform RIDDLE, and support cell viability during 3D growth, at least for the cell lines examined here.

## Discussion

Our studies conceptually advance the current mechanistic understanding of the UPR by shedding light on the fascinating, yet puzzling, process of IRE1-dependent mRNA decay. Although in fly cells this process is relatively unrestricted by substrate sequence, in mammalian cells it was thought to require an XBP1-like stem-loop endomotif^40–44^. Despite some tantalizing clues that human IRE1α may degrade a wider scope of mRNAs^43,51^, to date this apparent disparity has not been deciphered. Our dual next-generation sequencing strategy and validation studies, coupled with the discovery of a second basic endoribonuclease modality of IRE1α and its coordinate phospho-oligomeric state, enabled several advances: (1) a refinement of the canonical stem-loop endomotif; (2) the development of an algorithm to accurately discern such endomotifs in prospective mRNAs; and perhaps more importantly, (3) the identification of RIDDLE as a specific, biologically relevant activity of human IRE1α.

As previously considered^40–42^, the majority of RIDD substrates did not harbor secretion signals, and nor did the RIDDLE substrates identified here. We confirmed that IRE1α could colocalize with a RIDDLE-targeted mRNA in ER-stressed cells. Nevertheless, given that IRE1 is an ER-resident membrane protein, our observations raise the question of how it gains access to mRNAs that are not translated by membrane-bound ribosomes. Further work is required to determine whether such mRNAs reach the ER in a non-conventional manner—independent of a signal sequence—perhaps via sites of IRE1 clustering^65^. In addition, a fraction of IRE1 molecules may become selectively proteolyzed to allow severed IRE1-KR domains to venture into the cytosol and act on non-membrane-bound mRNAs^66,67^.

Transcripts decayed through both the RIDD and RIDDLE modalities showed enrichment in functional categories such as cell morphology and cell death or survival, which may be linked to the cellular response to ER stress. For example, DGAT2 promotes triglyceride synthesis^44^, while TNFAIP8L1 promotes apoptosis^68^.

Previous understanding divided IRE1’s endoribonuclease modality into XBP1 processing versus RIDD. It was further thought that variation in target-sequence selectivity for RIDD substrates was probably due to inter-species diversity. Our findings establish a distinct conceptual framework (Fig. 5e) based on the two endoribonuclease modalities of human IRE1α described here. The first modality, which minimally requires IRE1α dimerization, but can also be performed within phospho-oligomers (likely by the dimeric building blocks from which the oligomers assemble), carries out endomotif-specific RNA cleavage; the second, which strictly requires phospho-oligomerization, conducts more promiscuous endoribonuclease activity. The first modality enables both the dual cleavage of XBP1u and consensus-site cleavage of RIDD targets containing a robust XBP1-like stem-loop endomotif. The second modality mediates RIDDLE, which digests RNA substrates that either have a sub-optimal stem-loop endomotif, or lack one altogether. Importantly, RIDDLE also further degrades canonical endomotif-cleaved RIDD substrates. Our detailed experiments with DGAT2 and TNFAIP8L1 RNA suggest that RIDDLE favors GC sites; future studies should examine additional RIDDLE substrates to determine whether such sites are universal. Several miRNAs previously identified as RNase substrates of IRE1α were also found to be cleaved at GC sites^69^. Of note, these miRNAs did not meet the consensus criteria of the gRIDD algorithm (Supplementary Table 6). It will therefore be interesting to explore whether their cleavage by IRE1α involves the RIDDLE modality.

Our conceptualization aligns IRE1α multimer assembly with endoribonuclease modality. Phosphorylated oligomers performed RNase cleavage with better efficiency than dimers, pointing to a “rheostatic” nature of IRE1α activation, as previously suggested^34,35^. Accordingly, stronger ER stress could drive higher levels of IRE1α phospho-oligomerization, increasing catalytic efficiency for both XBP1s generation and RNA decay. Mutations that impaired phosphorylation and/or oligomerization confirmed and reinforced the requirement of distinct phospho-oligomeric states for IRE1α’s two basic RNase modes. Each phosphorylation site appeared similarly important for efficient RNA decay, with triple phosphorylation leading to the strongest activity. Although the non-phosphorylated IRE1-KR-0P was capable of cleaving XBP1u in vitro, perhaps because some B2B dimers still form in this setting, it could not perform RIDDLE upon concentration-driven oligomerization. This finding suggests that phosphorylation plays a specific role in RIDDLE beyond supporting dimer/oligomer assembly.

Our data in breast cancer cells differ from findings in B cells, wherein S729 proved uniquely critical for RIDD^50^. The correlation between RIDDLE and oligomerization both in vitro and in cells supports the possibility that deficient oligomerization and/or altered protomer alignment of the R887A mutant underlies its failure to conduct RIDDLE. Residing on human IRE1α B2B dimers, R887 may promote oligomer stabilization via double salt bridging with E876 residues on opposing B2B dimers. Consistent with this notion, overlay of the human IRE1α dimer onto the yeast oligomer places E876 within just 7 Å from R887 both in *cis* and towards the opposing protomers (Fig. 4d).

RNase activity of IRE1α may vary with substrate length. It is possible that dual XBP1u processing occurs more efficiently within phospho-IRE1α tetramers, which bind simultaneously to the two stem-loop endomotifs. Similarly, it is conceivable that for canonical RIDD targets, one IRE1α dimer binds to the stem-loop endomotif, while additional associated dimers within an oligomer cleave the transcript at additional locations. Future structural work is needed to explore oligomeric mammalian IRE1α alone and in complex with different RNAs. In addition, it will be important to investigate how the distinct RNase modalities unraveled here apply to IRE1 from different species and model organisms. In this context, it is interesting that yeast IRE1 was found to cleave HAC1/XBP1 mRNA as an oligomer, and to perform RIDD-like activity as a dimer^63^. However, this work relied primarily on predictions for dimer-interface mutants, without direct verification of actual oligomeric states. Emerging data for yeast IRE1 indicates that the positioning of the RNase domains within dimers regulates substrate recognition^70^.

Intriguingly, even the initial products of XBP1u processing underwent detectable degradation by IRE1-KR-3P, consistent with the previously proposed concept that XBP1 mRNA splicing involves kinetic competition between exon ligation and mRNA degradation^51^. This earlier study, however, identified components of the NO-GO and cytosolic exosome machineries as the exonucleases mediating further decay from the primary cut sites. Importantly, although additional endonucleolytic cleavages near ribosomes stalled on severed mRNA were uncovered, the nuclease(s) responsible for these cuts remained elusive. It has yet to be determined whether IRE1 plays a direct part in such clearing mechanisms by which cells rid themselves of defective mRNA. In the cellular environment, additional factors, including the Sec61 translocon^71^, could also shield processed XBP1u against further decay and thereby permit exon ligation by RtcB^72^.

Our experiments with the R887A mutant suggest that RIDDLE supports the role of IRE1α in enabling 3D growth of certain cancer cells. In concert, analysis of the TCGA database identified specific tumors having higher RIDD and RIDDLE gene expression scores (suggesting less depletion of the corresponding mRNAs) with significantly better patient prognosis (Supplementary Fig. 6 and Table 4). Moreover, based on the COSMIC database, two different cancer-associated mutations were identified at position R887 of human IRE1α (Supplementary Table 7).

In conclusion, our discovery and functional characterization of two fundamental enzymatic modalities of the IRE1α RNase advances the present conceptual framework for investigating how IRE1 operates across different eukaryotes. Our mechanistic dissection carries important implications for the biological understanding of the UPR and for harnessing IRE1α as a potential therapeutic target.

## Methods

### Cell culture and experimental reagents

MDA-MB-231, HCC1806, KMS-27, HCT116, and AMO-1 cells were all obtained from ATCC. U2OS WT and IRE1α KO cells were described in Belyy et al.^54^. All cells were authenticated by short tandem repeat (STR) profiles, and tested to ensure mycoplasma free within 3 months of use. All cell lines were cultured in RPMI1640 media supplemented with 10% (v/v) fetal bovine serum (FBS, Sigma), 2 mM glutaMAX (Gibco) and 100 U/ml penicillin plus 100 μg/ml streptomycin (Gibco).

Thapsigargin (Sigma) was used at a concentration of 100 nM. Compound 4µ8C and Compound 18 were dissolved in DMSO for cellular experiments and used at the indicated concentrations. Antibodies (Abs) for IRE1α (#3294), TGOLN2 (#95649), AIM2 (#12948), Actin (#5125), and GAPDH (#8884) from Cell Signaling Technology. CD59 (#133707), GBA (#125065), BCAM (#134110), HIP1 (#181238), TLR2 (#68159), SIX2 (#111827), SUOX (#129094), and BMP4 (#124715) from Abcam. BLOC1S1 (#19687-1-AP), OAS2 (#19279-1-AP), ALDH1A3 (#25167-1-AP), GPC1 (#16700-1-AP) from ProteinTech. pIRE1 and XBP1s antibodies were generated at Genentech. Secondary antibodies (rabbit #711-035-152 and mouse #715-035-150) were from Jackson ImmunoResearch Laboratories.

### CRISPR/Cas9 knockout: guide RNA sequences and technique

MDA-MB-231 *IRE1*α KO cells were generated using CRISPR by co-transfecting a Cas9 containing plasmid, pRK-TK-Neo-Cas9, with a pair of IRE1 targeting gRNAs (see below) cloned into a pLKO vector. Transfection was done using Lipofectamine 3000 according to manufacturer protocol, and transformants were selected by PCR on genomic DNA for the detection of deletions. Correct clones were then sequenced. *IRE1*α KO cl.1-2 gRNA pair: CTTGTTGTTTGTGTCAACGC & TCTTGCTTCCAAGCGTATAC.

### RNAseq/GROseq

Both RNAseq and GROseq were performed on WT and *IRE1*α KO MDA-MB-231 cells. For each RNAseq and GROseq there were four experimental conditions (WT and *IRE1*α KO, treated with Tg or vehicle control (DMSO) for 8 h with three biological replicates (*n* = 3) for each condition.

For RNAseq, RNA was extracted using the RNeasy kit (Qiagen #74104) performing on-column DNA digestion for 15 min. The concentration of RNA samples was determined using NanoDrop 8000 (Thermo Scientific) and the integrity of RNA was analyzed by Fragment Analyzer (Advanced Analytical Technologies). Approximately 500 ng of total RNA was used as input for library preparation using TruSeq RNA Sample Preparation Kit v2 (Illumina).

For GROseq, cells were pre-treated with 5-ethynyl uridine (EU) for 30 min prior to nuclei fractionation using Sigma kit (#NUC101) followed by Invitrogen Click-iT™ Nascent RNA Capture Kit protocol for nascent transcript extraction (#C10365). RNA was then extracted using the RNeasy kit (Qiagen #74104) performing on-column DNA digestion for 15 min and was subsequently reverse transcribed with Superscript VILO IV Master Mix from Invitrogen. Libraries were prepared following the protocol from NEBNext® Ultra™ II RNA Library Prep Kit for Illumina® (NEB #E7770S) and indexes (NEB #E7335S and #E7500S).

The size of the libraries for both RNAseq and GROseq was confirmed using 4200 TapeStation and High Sensitivity D1K screen tape (Agilent Technologies) and their concentration was determined by qPCR based method using Library quantification kit (KAPA). The libraries were multiplexed and then sequenced on Illumina HiSeq2500 (Illumina) to generate 30 M of single end 50 base pair reads.

### RNAseq-GROseq combined analysis

The DGEList function from the edgeR package (version 3.24.3) in R (version 3.5.1) was applied to the counts from the GRO-seq and RNA-seq datasets concatenated into a single dataset. Genes were kept if their counts per million exceeded 1.0 in at least three samples, and the calcNormFactors function was applied to the resulting count matrix. Negative binomial dispersions were computed using the estimateDisp function with the robust parameter set to true. Observations were log-transformed and weighted using the voomWithQualityWeights function from the limma package (version 3.38.3) in R. Statistics were computed using the lmFit, contrasts.fit, and eBayes functions for the contrast between the GRO-seq expression of wild-type samples treated with Tg at 8 h relative to DMSO, minus the RNA-seq expression of wild-type samples treated with Tg at 8 h relative to DMSO.

To identify IRE1α specific RIDD targets we compared genes’ differential expression in WT *versus* KO cells after 8 h Tg treatment: Log_2_ fold change (Log_2_(FC)), average expression, *p*-values, and false discovery rate (FDR) values were calculated for every protein-coding genes comparing the starting DMSO time point with 8 h after Tg treatment in WT and *IRE1*α KO conditions for both RNAseq and GROseq datasets. Log_2_(FC) in the RNAseq WT dataset for which the *p*-value and the FDR value were above 0.05 were removed. Log_2_(FC) difference between the RNAseq WT and *IRE1*α KO (Log_2_(KO-WT)) datasets falling below 0.5 were removed, and genes with average expression values below 1 were also removed. Finally, Log_2_(FC) differences between the RNAseq and GROseq WT (Log_2_(WTrna-WTgro)) falling below −0.3 were removed. The resulting gene list is 54 entries long.

### Signal sequence analysis

For each mRNA transcript selected as the representative candidate for a gene, we determined the corresponding protein sequence by running GMAP (version 2019-12-01)^73^ by aligning the sequence to itself using the -g flag and extracting the full-length protein translation with the flags “-P -F”. We then ran the program signalp (version 3.0)^74^ on the protein sequence with the flag “-t euk”, which yielded a signal sequence prediction and probabilities for the signal peptide, signal anchor, and cleavage site. Each mRNA was also checked in Uniprot for additional verification.

### Protein immunofluorescence staining with RNA in situ hybridization (ISH) cytospin

*IRE1α* WT and KO MDA-MB-231 cells were prepared as follows: cells were grown on 10 cm dishes, treated with Tg (100 nM), and collected at 0, 6, or 24 h time points. Cells were counted using ViaCell and resuspended in PBS at 1 × 10^6^/ml. Next, cells were centrifuged at 500 × *g* for 5 min at room temperature (RT) and PBS was removed almost completely without disturbing the pellet. The pellets were resuspended by gentle pipetting into the appropriate volume of NBF (10%v/v Neutral Buffered Formalin) to obtain a cell concentration of 1 × 10^6^/ml and incubated for 30 min at 37 °C. Next, samples were centrifuged at 500 × *g* for 5 min at RT, NBF was removed and the cells were washed twice in PBS. After the last PBS wash, cells were resuspended in ice cold 70% ethanol solution at 1 × 10^6^/ml and stored at 4 °C until used for analysis (max 1 month). Samples were centrifuged at 800 RCF for 10 min and the slides were removed from the cytoprep kit. Slides were air-dried for 20 min at RT and dehydrated in 50, 70, and 100% ethanol in preparation for staining.

### Automated procedures

Automated cytospin ISH is a modified single ISH protocol from Advanced Cell Diagnostics RNAScope 2.5 LS Reagent Kit-Red User Manual (ACD, UM-322150 RevA), performed using a Leica Bond-RX system. Pretreatment steps were adjusted to maintain an optimal morphology for cytospin samples. Fluorescent ISH procedure was modified from ACD protocol in the amplification steps.

### Sample pretreatment

After cytospin, slides were removed from 100% ethanol and dried for 30 min in an oven at 37 °C. Slides were labeled with ACD2.5 Red Rev B protocol (without counterstaining step) and inserted into the Bond RX slides racks tray to be processed. Select the “frozen slide delay” as preparation protocol to accommodate the overnight delay run. Antigen retrieval was conducted with *ACD HIER 15 min with ER2 at 88 °C (Bond Epitope Retrieval Solution 2; Leica Cat#AR9640). The enzyme digestion step was omitted to avoid over-digestion of the sample. Endogenous peroxidase activity was quenched with RNAScope 2.5 LS hydrogen peroxide for 10 min at RT and washed twice with 1X Bond wash buffer (Leica 10X concentrate Cat#AR9590). Peroxide quenching step was re-added to the hybridization protocol as a workaround because enzyme treatment and quench steps were linked in the automated program. Therefore, the removal of the enzyme treatment also leads to the removal of the quenching step. This workaround is not necessary if the enzyme digestion step is not eliminated.

### Fluorescent dual ISH/ICC procedure

Dual fluorescent cytospin ISH/ICC (immunocytochemistry) procedure^75^ is a modified staining protocol of single chromogenic RNAScope LS 2.5 Red detection (322150-USM) using RNAScope 2.5 LS reagent kit (ACD, 322150). Following sample pretreatment, hybridization and amplification steps were done according to the RNAScope LS2.5 protocol (ACD, UM-322150 RevA) (see Supplementary Table 8). Probes were hybridized for 2 h at 42 °C. Slides were washed with 1X Bond wash buffer (Leica 10X concentrate, AR9590) at 42 °C 3 times (0, 1, 5 min) followed by eight washes with 1× Bond wash buffer 0 min each. Samples were processed only to the end of the Amplification 4 step (*ACD Amp4) followed by washes. ISH detection was completed using Opal-570 (1:1500) in 1× amplification buffer (PerkinElmer, NEL794001KT) 1 and 10 min each at RT. Slides were washed with 1× Bond wash solution 3 times 0 min each followed by additional 5 times 1 min each at RT. Slides were then rinsed 2 times with deionized water and continued to ICC procedure.

Upon completion of ISH detection, slides were again treated with RNAScope LS2.5 Hydrogen Peroxide to quench endogenous peroxidase for 10 min at RT and three washes with 1× Bond wash buffer. Slides were incubated with TNB blocking (0.1 M Tris-HCl, pH 7.5, 0.15 M NaCl, 0.5% Blocking Reagent PerkinElmer, FP1012) for 30 min at RT. Primary antibody was incubated 60 min at RT. Slides were then open washed 3 times with 1× Bond wash buffer. HRP-conjugated secondary antibody was added for 30 min at RT, and then six open washes with 1× Bond wash solution were performed. Final detection step was conducted with PerkinElmer Opal-690 dye (1:1500) in 1× amplification buffer incubated for 30 min at RT. Excess dye was removed with eight open washes with Bond wash solution. Spectral DAPI (PerkinElmer. FP1490) counterstain was performed for 5 min at RT. Excess DAPI was rinsed off by five washes with deionized water. Finally, the slides were cover slipped with Prolong Gold anti-fade reagent (Life Technology Cat# P36930) or with Tissue Tek Mounting Medium (Sakura, cat#6419) xylene-based permanent mounting medium.

### RT-qPCR

RNA was extracted using the RNeasy Plus kit (Qiagen #74134). Equal amounts of RNA were reverse transcribed and amplified using the TaqMan™ RNA-to-CT™ 1-Step Kit (Applied Biosystems #4392938) on the ABI QuantStudio 7 Flex Real-Time PCR System. The delta-delta C_T_ values were calculated by relating each individual C_T_ value to its internal GAPDH control. Taqman primers for XBP1u (#Hs02856596_m1), XBP1s (#Hs03929085_g1), DGAT2 (#Hs01045913_m1), BLOC1S1 (#Hs00155241_m1), CD59 (#Hs00174141_m1), and TNFAIP8L1 (#Hs00537038_m1), and GAPDH (#Hs02758991_g1) were from Life Technology. Additional primer pairs used for qPCR from cell rescue experiments were ordered from IDT: TNFAIP8L1 (#Hs.PT.58.39992641), SNN (#Hs.PT.58.28146300), SIX2 (#Hs.PT.58.40614621), GAPDH (#Hs.PT.39a.22214836), and custom-designed (see Supplementary Table 9).

### T7 RNA constructs

We prepared T7 RNA transcripts from cDNA templates chosen based upon functional relevance coupled with optimal length for the ribonucleolytic reaction (~0.5–2 kb). cDNA constructs encoding XBP1 (#HG10751-UT), DGAT2 (#HG14114-G), CD59 (#HG12474-UT), TGOLN2 (#HG17252-UT), SIX2 (#HG21116-UT), CFAP45 (#HG22377-UT), MFAP2 (#HG16644-UT), PIGQ (#HG22757-UT), BMP4 (#HG10609-UT), BCAM (#HG10238-UT), SNN (#HG23279-U), GBA (#HG12038-UT), WT1 (#HG12282-UT), CCDC69 (#HG27177-U), AIM2(#HG11654-UT) were from Sino Biological, and BLOC1S1 (#RC224412), TNFAIP8L1 (#RC203912) from Origene. cDNA was amplified using T7 forward primers, and subsequently in vitro transcribed using HiScribe™ T7 Quick High Yield RNA Synthesis Kit from NEB (#E2050S).

T7 RNA mutations were engineered using overlap PCR followed by restriction digests, and the final fragments were purified from agarose gel (Zymoclean Gel DNA Recovery kit #D4001).

### Protein purification and separation of phosphorylated IRE1α fractions

IRE1α KR 0P and 3P were produced by Accelagen and in-house: IRE1α KR (G547-L977) was expressed as N-terminal His_6_-tagged fusion proteins in SF9 cells with a TEV protease cleavage site from an intracellular BEVS expression vector. Cell pellet was resuspended in lysis buffer containing 50 mM HEPES pH 8.0, 300 mM NaCl, 10% glycerol, 1 mM MgCl_2_, 1:1000 benzonase, EDTA-free PI tablets (Roche), 1 mM TCEP, and 5 mM imidazole. The sample was lysed by sonication, centrifuged at 12,000 × *g* for 45 min, and the supernatant filtered through a 0.8 μm Nalgene filter. Cleared supernatant was bound to Ni-NTA Superflow beads (Qiagen) by gravity filtration. Beads were washed in lysis buffer supplemented with 15 mM imidazole, followed by protein elution in lysis buffer containing 300 mM imidazole. The eluate was incubated with TEV protease overnight at 4 °C. The sample of IRE1α KR protein was diluted 1:10 in 50 mM HEPES pH 7.5, 50 mM NaCl, 1 mM TCEP, and then loaded onto a 5 mL pre-packed Q-HP column (GE-Healthcare). The separation of IRE1α KR unphosphorylated and phosphorylated was achieved by eluting the protein with a very shallow gradient (50-300 mM NaCl over 70CV). Fully phosphorylated fraction (MW + 240 by LC-MS) was collected separately, while the rest of the protein fractions were consolidated and incubated with Lambda phosphatase for one hour at room temperature. Dephosphorylation was confirmed by LC-MS. Unphosphorylated and phosphorylated samples were then concentrated and loaded separately onto a HiLoad 16/600 Superdex 200 SEC column (GE Healthcare) equilibrated in 25 mM HEPES pH 7.5, 250 mM NaCl, 1 mM TCEP, 10% glycerol. IRE1α eluted as a monomer.

Mutants S724A, S726A, S729A, S724A-S726A, S724A-S729A, S726A-S729A, and R887A were produced in-house following the same procedure described above.

Phosphorylation site mapping was performed by LC-MS/MS analysis following protease digestion (see Supplementary Table 10).

### Phosphorylation of IRE1α KR and activation loop mutants

IRE1α KR S/A and R887A mutants were allowed to autophosphorylate in the presence of 2 mM ATP and 10 mM MgCl_2_ for one hour at room temperature. The sample was purified from ADP by size-exclusion chromatography (SEC).

Mutant S726A-S729A was not able to autophosphorylate and was instead incubated with pIRE1α LKR (Linker-Kinase-RNase, residues Q470-L977) at 1:40 w/w, 2 mM ATP and 20 mM MgCl_2_. Final phosphorylated proteins were purified from pIRE1α LKR and residual nucleotides by SEC.

### RNA cleavage assay

One microgram of T7 RNA was digested at room temperature by 1 μg of human IRE1α KR recombinant protein (~0.8 μM final) for 15 min in RNA cleavage buffer (HEPES pH 7.5 20 mM; K acetate 50 mM; Mg acetate 1 mM; TritonX-100 0.05% (v/v)). The total volume of the reaction is 25 μl. The digestion was then complemented by an equal volume of formamide and heated up at 70 °C for 10 min to linearize the RNA. After linearization, the mixture was immediately placed on ice for 5 min, and then 20 μl was run on 3% agarose gel at 160 V for 50 min at 4 °C. If inhibitors were used (5 µM), they were incubated with the RNA for 40 min on ice prior to RNA digestion. Gels were visualized on a BioRad Molecular Imager ChemiDoc ZRS+.

### RNA fragment sequencing

Two micrograms of T7 RNA (TNFAIP8L1, DGAT2) is used for digestion by human IRE1α KR-3P as described above. RNA bands are extracted from gel using Zymoclean Gel RNA Recovery Kit (Zymo Research #R1011). Next, the RNA extracted was ligated using RtcB ligase (NEB # M0458S) to a 3′-adapter oligo custom designed and ordered from IDT (caagcagaagacggcatacgagatCGTGAT), following manual protocol. Ligated RNA was then reverse transcribed using SuperScript™ IV First-Strand Synthesis System (Invitrogen #18091050) and a 3′-adapter specific primer (ATCACGatctcgtatgccg). cDNA was then amplified using DGAT2 (gggGCATGCATGAAGACCCTCATAGCCG) or TNFAIP8L1 (gggGCATGCATGGACACCTTCAGCACCAAG) specific forward primer containing an SphI restriction enzyme site, and a common reverse primer containing a EcoRI restriction enzyme site (gggGAATTCATCACGatctcgtatgccg). PCR product were subsequently digested by SphI and EcoRI prior cloning into a pGEM®-T Easy Vector (Promega #A1360). Then, resulting plasmids are transfected into competent cells (Zymo *Mix & Go!* Competent Cells Zymo 10B #T3019), plated on a 10 cm dish and left at 37 °C overnight. The following day, individual colonies are picked and grown onto 96 wells plates, designed for bacterial growth (Thomson Instrument Company #951657), overnight. Finally, DNA is extracted from individual wells using Zyppy-96 Well Plasmid Miniprep Kit (Zymo Research #D4042) and sent for SANGER sequencing (see Supplementary Data 1).

### RNase activity assay (kinetic fluorescence)

A 5′-Carboxyfluorescein (FAM)-and 3′-Black Hole Quencher (BHQ)-labeled single stem-loop mini-substrate containing XBP1 sequence (5′FAM-CAUGUCCGCAGCGCAUG-3′BHQ) was used as substrate for cleavage by IRE1α KR (G547-L977) in 20 mM HEPES pH 7.5, 50 mM potassium acetate, 1 mM magnesium acetate, 1 mM dithiothreitol, 0.05% v/v TritonX-100. 10 nM of protein was incubated with varying concentrations of RNA substrate (twofold dilution series from 3000 to 5.86 nM). RNA cleavage was measured kinetically over an hour at room temperature as an increase in fluorescence. The final reaction was carried out in 20 μL in 384-well plates. Samples were run in duplicate. The velocity of the reaction was measured as the slope of the linearly increasing fluorescence signal over time as relative fluorescence units (RFU)/s and plotted as a function of RNA substrate concentration. Michaelis-Menten kinetics were fit using Prism 1.7 and resulting Vmax and Km constant were reported.

### Immunoblot analysis

Cells were lysed in 1× RIPA buffer (Millipore) supplemented with fresh protease and phosphatase inhibitors (Invitrogen #78440), cleared by centrifugation at 12,000 × *g* for 15 min, and analyzed by BCA protein assay (Thermofisher Scientific #23227). Equal protein amounts were loaded, separated by SDS-PAGE, electrotransferred to nitrocellulose membranes using the iBLOT2 system (Invitrogen), and blocked in 5% nonfat milk solution for 30 min. Membranes were probed with the required antibodies. Signal was detected using appropriate horseradish peroxidase (HRP)-conjugated secondary antibodies. All primary antibodies were used at 1:000 dilution and overnight hybridization at 4 °C, followed by a two-hour incubation with horseradish peroxidase (HRP)-conjugated secondary antibodies at 1:10,000 dilution.

### Crosslinking assay

In vitro: 1 μg of IRE1-KR recombinant protein was crosslinked in 25 μl final volume of RNA cleavage buffer containing 1 μl of disuccinimidyl suberate (DSS, Thermo Fisher Scientific) crosslinker at 6.25 mM (final concentration is 250 μM) for 1 h on ice. For experiments recurring inhibitor and activator compounds, IRE1α KR and the small molecule compound (5 µM) were first pre-incubated together on ice for 40 min. The reaction was quenched using 1 μl of a pH 7.5, 1 M TRIS solution for 15 min on ice. The reaction was then diluted in 500 μl of RNA cleavage buffer and 13 μl of it was used to run on SDS-PAGE gel (corresponding to ~25 μg of protein. The gel was run at 100 V for almost 3 h then electro-transferred to nitrocellulose membranes using the iBLOT2 system (Invitrogen), and blocked in 5% nonfat milk solution. Finally, it was incubated overnight at 4 °C with an IRE1α antibody (Cell Signaling, #3294S) at 1:1000 dilution, followed by a two-hour incubation with horseradish peroxidase (HRP)-conjugated secondary antibodies at 1:10,000 dilution.

In vivo: Cells were lysed in 100 μl 1% Triton X-100 in PBS supplemented with fresh protease and phosphatase inhibitors (Invitrogen #78440), incubated on ice for 10 min, and cleared by centrifugation at 12,000 × *g* for 15 min. Seventy microliters of lysate was crosslinked with 0.7 μl DSS (Thermo Fisher Scientific) crosslinker (250 μM final concentration) for 1 h at RT. The reaction was quenched using 3.5 μl of a pH 7.5, 1 M TRIS solution for 15 min at RT. Protein concentration was determined using BCA assay, and at least 25 μg was used to run on SDS-PAGE gel for western blotting.

### Gel fractionation

Hundred micrograms of IRE1-KR-3P were crosslinked with DSS for 30 min on ice. The reaction was then loaded on Invitrogen 4–16% NativePAGE gel at 4 °C for 4 h at 100 V. Subsequently, the gel was cut in pieces at locations corresponding to monomer, dimer, and oligomer fractions. Gel fractions were then put in 100 μl of RNA cleavage buffer containing 4 μg of the T7 RNA transcript to digest overnight at 4 °C. Finally, 10 μl of the reaction was used and run on a 3% agarose gel for visualization.

### Cellular IRE1α rescue

MDA-MB-231 or HCC1806 shIRE1α cell lines were transfected with a 2 kb IRE1α promoter-driven - shIRE1α resistant - GFP/His tagged IRE1α WT or R887A mutant - Neomycin resistant construct, using Mirus TransIT-X2 delivery system on six-well plates. After 24 h cells were transferred to individual T75 flasks for 4 days. Media was changed and cells were selected using Geneticin at 1.5 mg/ml final for roughly 10 days, then FACS sorted for GFP positive cells.

### Viability assay

In total, 4000 cells were plated in four replicates on 96-well Corning plates either standard flat clear-bottom or ULA (#7007). At plating, cells were treated with a 0.4 μg/ml Doxycycline (Clonetech) final concentration in 200 μl total volume. Seven days later, 100 μl of media was taken out very carefully from each plate, not disturbing the spheroids from the ULA plate. Cell viability was then assessed by CellTiter-Glo 3D, adding 100 μl of buffer (Promega #G9683) to each well and pipetting up and down a few times, and measured in a luminescence reader (Envision; PerkinElmer). The data depicted as Relative viability after Dox treatment is calculated from the means of quadruplicate samples, wherein viability of Dox treated cells is divided by that of untreated cells (ratio) and normalized to 2D mean viability.

### TCGA analysis

RNA-seq data were taken from 635 normal and 6731 tumor samples across 20 tissue types in TCGA. RIDD and RIDDLE scores were computed by taking the mean Z-score for the signature genes. RIDD scores were computed by taking the mean Z-score for the genes: BLOC1S1 (Entrez Gene ID 2647), PIGQ (9091), TGOLN2 (10618), DGAT2 (84649), WT1 (7490), GBA (2629), CD59 (966), and BMP4 (652). RIDDLE scores were computed by taking the mean Z-score for the genes: BCAM (Entrez Gene ID 4059), CCDC69 (26112), MFAP2 (4237), SNN (8303), SIX2 (10736), AIM2 (9447), OAS2 (4939), CFAP45 (25790), and TNFAIP8L1 (126282). Survival data for TCGA samples were obtained from the National Cancer Institute GDC Legacy Archive at http://portal.gdc.cancer.gov/legacy-archive by extracting the 225 files that had clinical data in Biotab format. Right-censored overall survival was determined from fields marked “days_to_death”, “death_days_to”, “days_to_last_followup”, “last_contact_days_to”, and “vital_status”, and matched to the RNA-seq data by the patient barcode to provide survival data on 7283 (98.9\%) of the 7366 samples. For each normal and tumor disease type, patients were divided into two approximately equal groups, depending on whether their RIDD or RIDDLE score was higher or lower than the median within that disease type. A Cox model was fit using the coxph function from the survival package (version 2.44-1.1) in R (version 3.5.1). *P*-values were obtained from applying the summary function to the Cox model. Survival curves were generated using the plot function in R on the object produced by the survfit function. Cancers showing significantly different survival (*p* < 0.01) were illustrated in Fig. S6.

### Statistics and reproducibility

All values are represented as mean (SEM) with at least two independent biological replicates with at least two technical replicates. Statistical analysis of the results was performed by unpaired, two-tailed *t* test or two-way ANOVA. A *P* value ≤0.05 was considered significant, and denoted by *P ≤ 0.05; **P ≤ 0.01. All statistical analyses were performed using GraphPad Prism 9 (GraphPad Software, Inc.). RNA cleavage assays and Western Blots have been repeated independently at least 3 times with similar results.

### Reporting summary

Further information on research design is available in the Nature Research Reporting Summary linked to this article.

## Supplementary information

Supplementary Information

Description of Additional Supplementary Files

Supplementary Dataset 1

Supplementary Software 1

Reporting Summary

## Data Availability

The data supporting the findings of this study are available from the corresponding authors upon reasonable request. Raw sequences and processed data that support the findings of this study are now available at NCBI GEO as GSE169585. Source data for each figure are provided with this paper as a Source Data file.

## Code availability

The gRIDD program is available as a Supplementary Software. All other analysis codes have been previously reported and are publicly available.
