# Supplementary material for: Decoding non-canonical mRNA decay by the endoplasmic-reticulum stress sensor IRE1α
Source: Nat Commun. 2021 Dec 15;12:7310. doi: 10.1038/s41467-021-27597-7 (PMC8674358; doi:10.1038/s41467-021-27597-7)
Supplement: Supplementary file 1 — Supplementary Information [file 41467_2021_27597_MOESM1_ESM.pdf]

Decoding non-canonical mRNA decay by the endoplasmic-reticulum stress sensor IRE1α

Adrien Le Thomas[1], Elena Ferri[2,3], Scot Marsters[1], Jonathan M. Harnoss[1], David A. Lawrence[1], Iratxe Zuazo-Gaztelu[1], Zora Modrusan[4], Sara Chan[5], Margaret Solon[5], Cécile Chalouni[5], Weihan Li[6,7], Hartmut Koeppen[5], Joachim Rudolph[3], Weiru Wang[2], Thomas D. Wu[8], Peter Walter[6,7], and Avi Ashkenazi[1]

Departments of [1]Cancer Immunology, [2]Structural Biology, [3]Discovery Chemistry, [4]Microchemistry, Proteomics and Lipidomics, and [5]Pathology, Genentech, Inc., 1 DNA Way, South San Francisco, CA USA 94080

[6]Howard Hughes Medical Institute and [7]University of California, San Francisco, CA USA 94158,

[8]Department of Oncology Bioinformatics Genentech, Inc., 1 DNA Way, South San Francisco, CA USA 94080

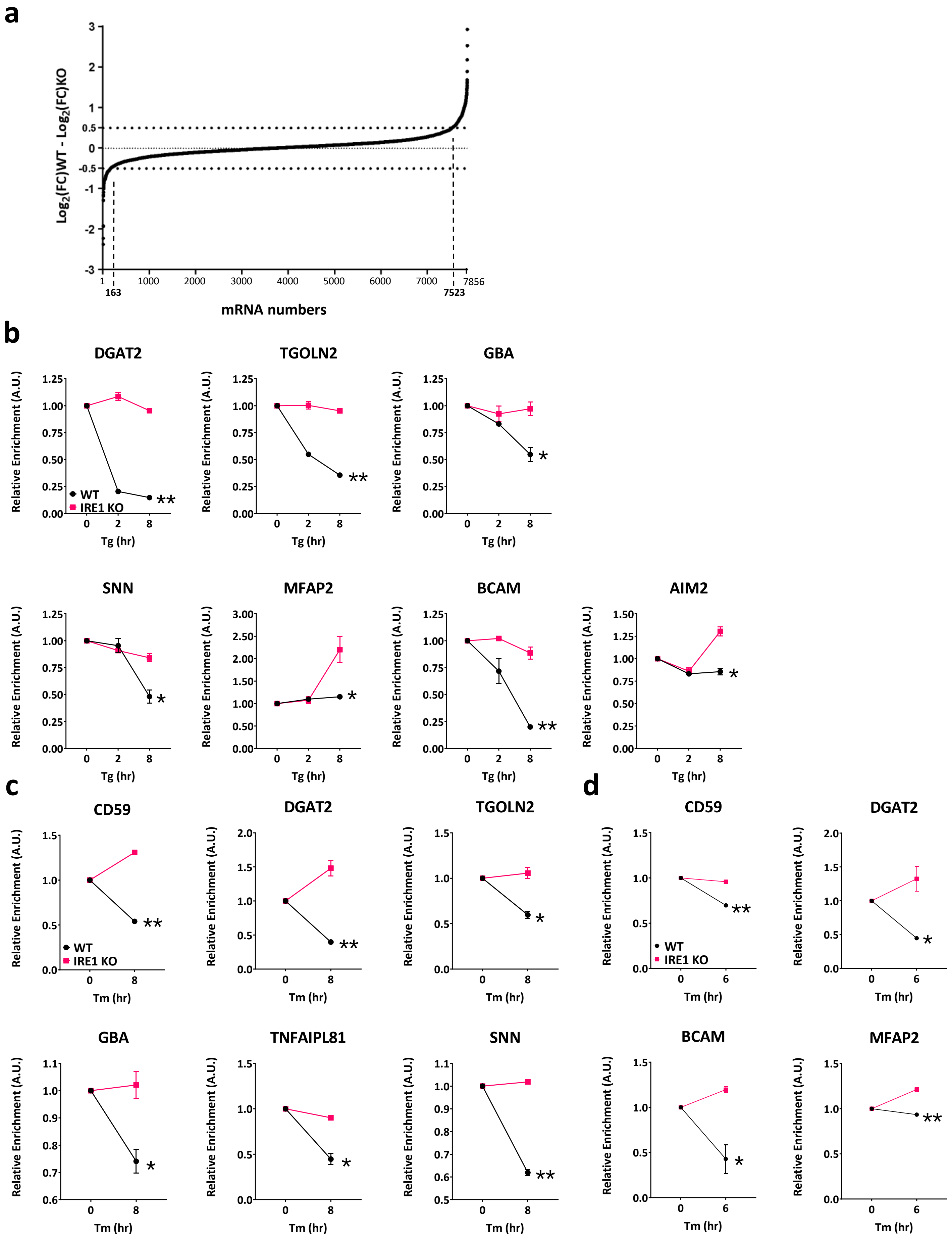
a
Log2(FC)WT - Log2(FC)KO
mRNA numbers
163
7523
7856
b
DGAT2
TGOLN2
GBA
SNN
MFAP2
BCAM
AIM2
Relative Enrichment (A.U.)
Tg (hr)
WT
IRE1 KO
c
CD59
DGAT2
TGOLN2
GBA
TNFAIPL81
SNN
Tm (hr)
d
CD59
DGAT2
BCAM
MFAP2

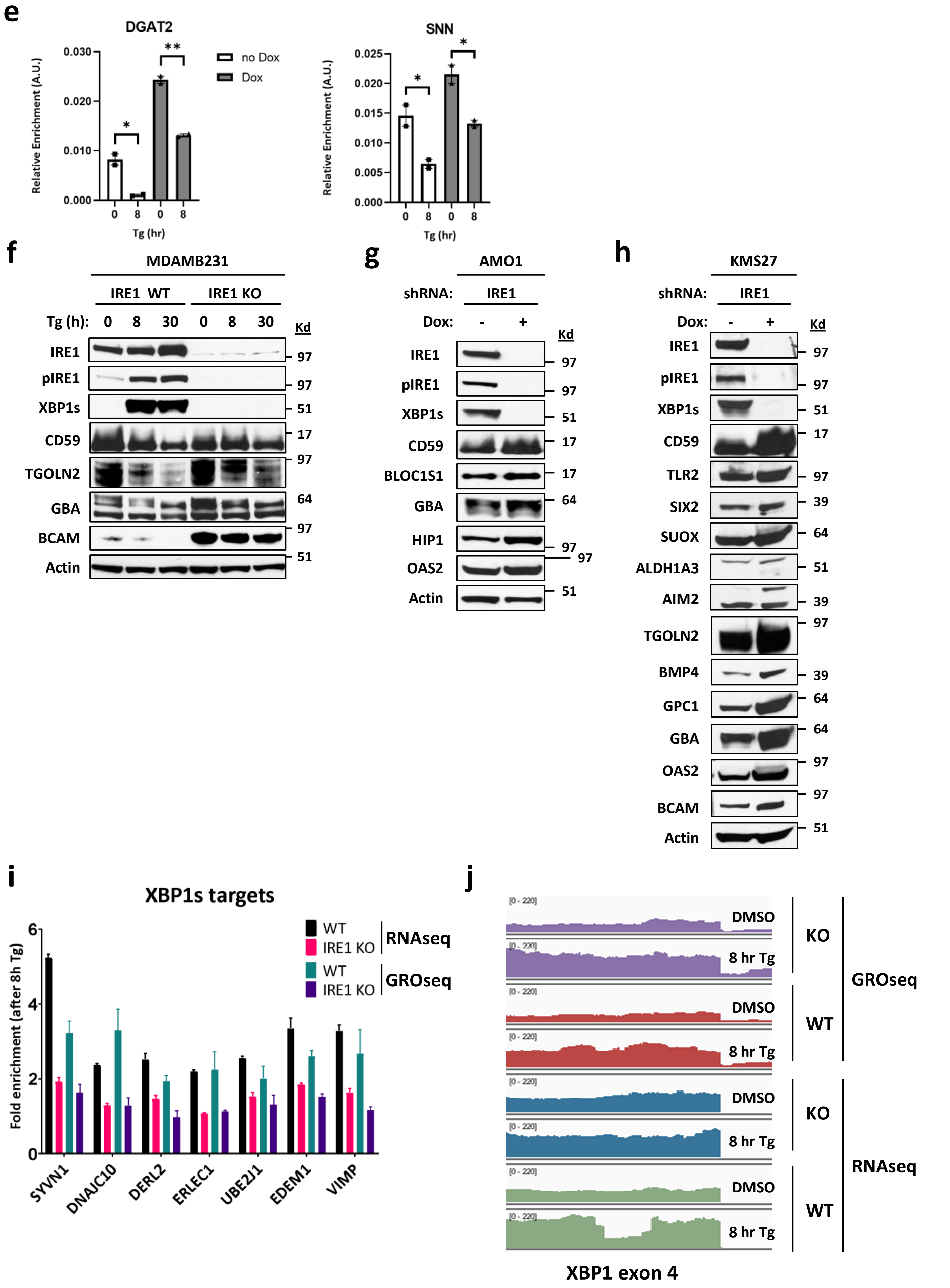
e
DGAT2
Relative Enrichment (A.U.)
0.030
0.020
0.010
0.000
no Dox
Dox
0 8 0 8
Tg (hr)
SNN
0.025
0.020
0.015
0.010
0.005
0.000
f
MDAMB231
IRE1 WT
IRE1 KO
Tg (h): 0 8 30 0 8 30
Kd
IRE1
pIRE1
XBP1s
CD59
TGOLN2
GBA
BCAM
Actin
97
97
51
17
97
64
97
51
g
AMO1
shRNA: IRE1
Dox: - +
Kd
IRE1
pIRE1
XBP1s
CD59
BLOC1S1
GBA
HIP1
OAS2
Actin
h
KMS27
shRNA: IRE1
Dox: - +
Kd
IRE1
pIRE1
XBP1s
CD59
TLR2
SIX2
SUOX
ALDH1A3
AIM2
TGOLN2
BMP4
GPC1
GBA
OAS2
BCAM
Actin
i
XBP1s targets
Fold enrichment (after 8h Tg)
WT
IRE1 KO
RNAseq
WT
IRE1 KO
GROseq
SYVN1
DNAJC10
DERL2
ERLEC1
UBE2J1
EDEM1
VIMP
j
[0 - 220]
DMSO
8 hr Tg
KO
WT
GROseq
RNAseq
XBP1 exon 4

Supplementary Figure 1. Integrative RNAseq and GROseq analyses identify human RIDD and RIDDLE targets. (**a**) IRE1-dependent Log_2 fold change in mRNA levels after Tg treatment of MDA-MB-231 cells, as determined by RNAseq. (**b**) Kinetic RT-qPCRs analysis of gene transcripts identified through integrative RNAseq and GROseq analysis in *IRE1α* WT and KO MDA-MB-231 cells after ER stress induction by Tg (100 nM). n=2 biologically independent experiments. Data are presented as mean values +/- SEM. An unpaired t-test was used to calculate p-values. (**c**) Kinetic RT-qPCRs analysis in *IRE1α* WT and KO MDA-MB-231 cells after ER stress induction by Tunicamycin (Tm, 1 µg/ml). n=2 biologically independent experiments. Data are presented as mean values +/- SEM. An unpaired t-test was used to calculate p-values. (**d**) Kinetic RT-qPCRs analysis in *IRE1α* WT and KO U2OS cells after ER stress induction by Tunicamycin (Tm, 1 µg/ml). n=2 biologically independent experiments. Data are presented as mean values +/- SEM. An unpaired t-test was used to calculate p-values. (**e**) Kinetic RT-qPCRs analysis in *IRE1α* WT and KO HCT116 cells after ER stress induction by Tg (100 nM). n=2 biologically independent experiments. Data are presented as mean values +/- SEM. An unpaired t-test was used to calculate p-values. (**f**) Kinetic immunoblot analysis comparing RIDD and RIDDLE targets in *IRE1α* WT and KO MDA-MB-231 cells after Tg treatment (100 nM). For TGOLN2 and GBA, multiple bands are detected, consistent with the existence of multiple splice variants. (**g-h**) Immunoblot analysis comparing RIDD and RIDDLE targets in AMO1 (g) and KMS27 (h) cell lines under doxycycline induced shRNA knock-down of IRE1α or XBP1 (g), or IRE1α (h). (**i**) Fold enrichment after ER stress induction by Tg for specific XBP1s target genes in IRE1α WT and KO MDA-MB-231 cells, as determined from the RNAseq and GROseq datasets. n=3 biologically independent experiments. Data are presented as mean values +/- SEM. (**j**) Read coverage around the spliced XBP1 genomic region (part of exon 4) in all various datasets. *, $P \le 0.05$; **, $P \le 0.01$.

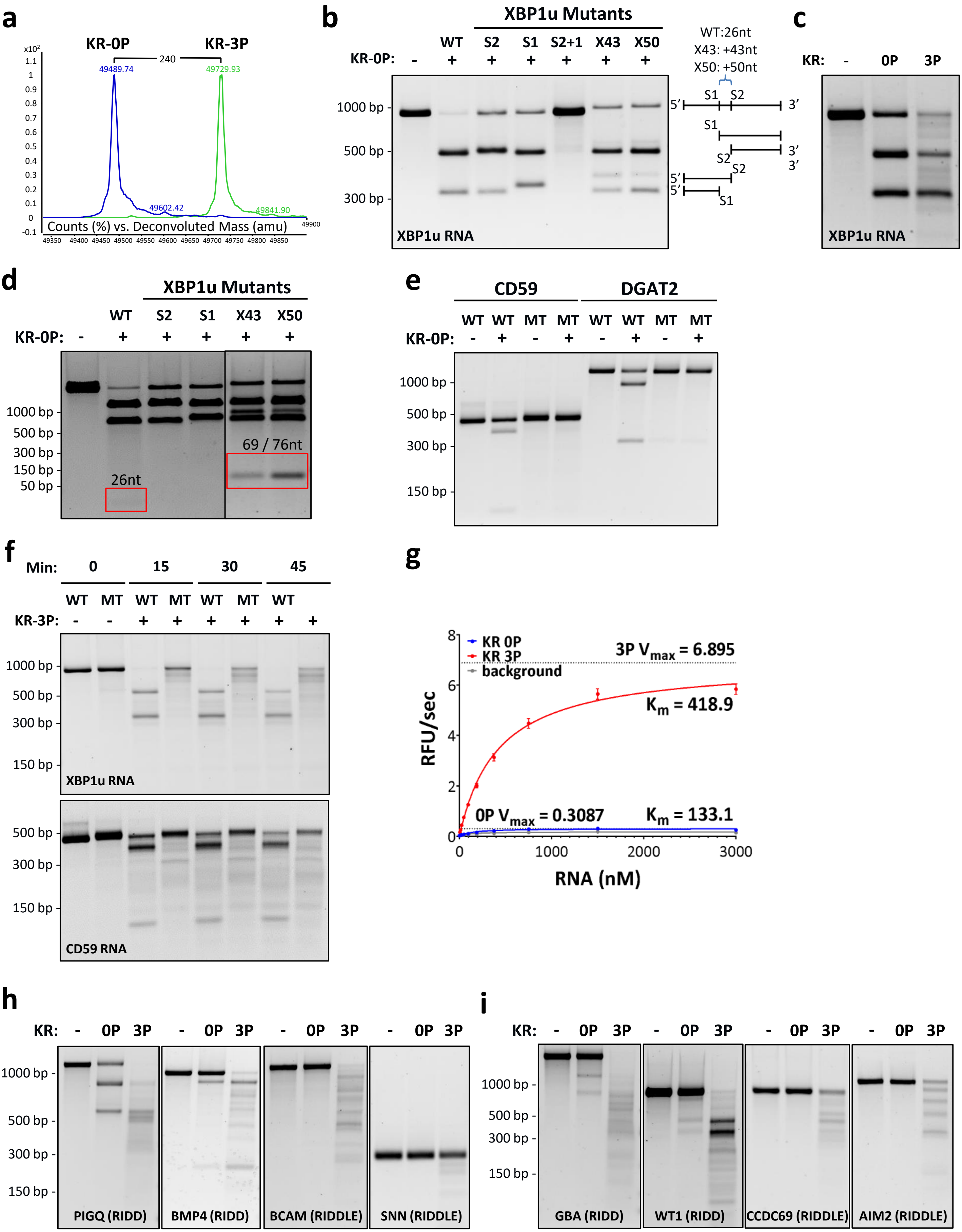
a
KR-0P
KR-3P
240
49489.74
49729.93
49602.42
49841.90
Counts (%) vs. Deconvoluted Mass (amu)
b
XBP1u Mutants
KR-0P: - + + + + + +
WT S2 S1 S2+1 X43 X50
1000 bp
500 bp
300 bp
XBP1u RNA
WT:26nt
X43: +43nt
X50: +50nt
5' S1 S2 3'
c
KR: - 0P 3P
XBP1u RNA
d
XBP1u Mutants
WT S2 S1 X43 X50
KR-0P: - + + + + +
1000 bp
500 bp
300 bp
150 bp
50 bp
26nt
69 / 76nt
e
CD59
DGAT2
WT WT MT MT WT WT MT MT
KR-0P: - + - + - + - +
1000 bp
500 bp
300 bp
150 bp
f
Min: 0 15 30 45
WT MT WT MT WT MT WT
KR-3P: - - + + + + + +
1000 bp
500 bp
300 bp
150 bp
XBP1u RNA
CD59 RNA
g
KR 0P
KR 3P
background
3P V$_{max}$ = 6.895
K$_m$ = 418.9
0P V$_{max}$ = 0.3087
K$_m$ = 133.1
RFU/sec
RNA (nM)
h
KR: - 0P 3P
1000 bp
500 bp
300 bp
150 bp
PIGQ (RIDD)
BMP4 (RIDD)
BCAM (RIDDLE)
SNN (RIDDLE)
i
KR: - 0P 3P
1000 bp
500 bp
300 bp
150 bp
GBA (RIDD)
WT1 (RIDD)
CCDC69 (RIDDLE)
AIM2 (RIDDLE)

j

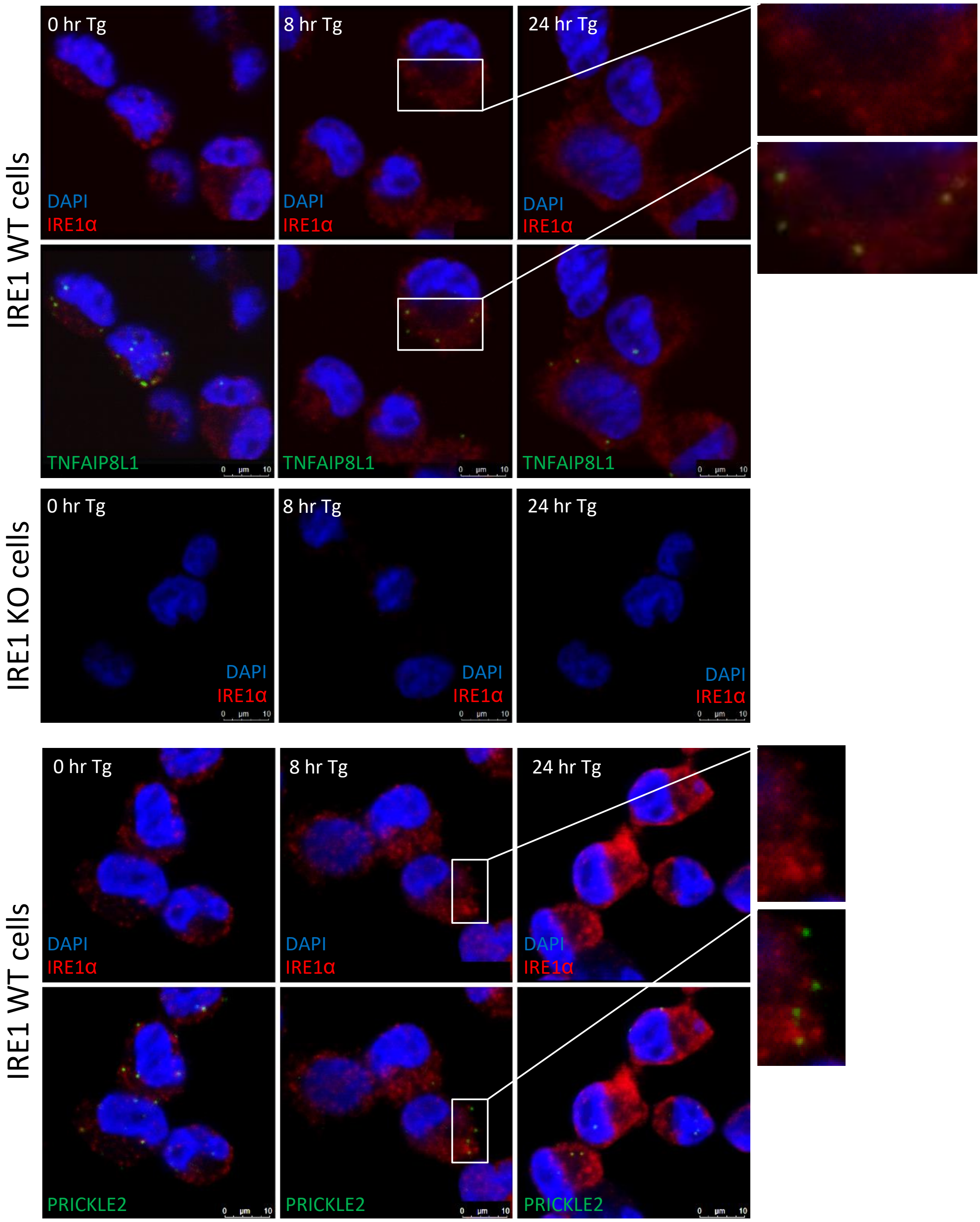
IRE1 WT cells
0 hr Tg
8 hr Tg
24 hr Tg
DAPI
IRE1α
TNFAIP8L1
0 μm 10
IRE1 KO cells
0 hr Tg
8 hr Tg
24 hr Tg
DAPI
IRE1α
0 μm 10
IRE1 WT cells
0 hr Tg
8 hr Tg
24 hr Tg
DAPI
IRE1α
PRICKLE2
0 μm 10

k

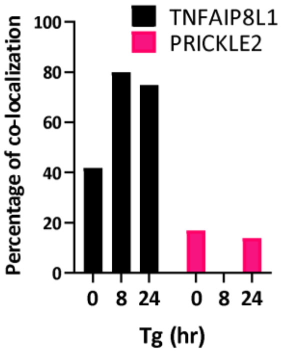
TNFAIP8L1
PRICKLE2
Percentage of co-localization
100
80
60
40
20
0
0 8 24 0 8 24
Tg (hr)

Supplementary Figure 2. Phosphorylation state of IRE1α affects RNase modality. (**a**) Each IRE1-KR protein was purified by anion exchange followed by size exclusion chromatography and its identity and phosphorylation state were verified by liquid chromatography-mass spectrometry (LC-MS). Pre-existing phosphates were removed by treatment with λ-phosphatase (KR-0P). (**b**) IRE-KR-0P digestion of XBP1u transcript variants: WT; loop motif 2 scrambled (S2); loop motif 1 scrambled (S1); loop motif 2 and 1 scrambled (S2+1); intron with 43-nt (X43) or 50-nt (X50) random sequence inserted. Schematic illustration of each variant is depicted on the right side of the gel aligned with corresponding products. (**c**) IRE-KR-0P and IRE1-KR-3P digestion of XBP1u RNA. Share the same molecular weight markers as in (**b**). (**d**) IRE1-KR-0P digestion of XBP1u variants at higher concentrations to visualize resulting spliced fragments (highlighted in the red boxes with their respective expected size). (**e**) IRE1-KR-0P digestions of CD59 and DGAT2 RNA transcripts in WT and scrambled endomotif mutant (MT) versions. (**f**) IRE1-KR-3P digestions of WT or MT (scrambled endomotif) versions of XBP1u, and CD59 RNAs were performed for the indicated time and analyzed as above. (**g**) Michaelis-Menten kinetics for RNase activity of IRE1-KR 0P (blue) and IRE1-KR-3P (red). Each IRE1-KR protein (10 nM) was incubated for 1 hr at room temperature with quenched fluorescein-conjugated RNA substrate at varying concentrations. As the substrate is cleaved, FAM fluorescence is emitted and can be measured at regular intervals during the incubation (see methods section). Velocity was measured as Relative Fluorescent Units (RFU)/sec and is shown as a function of RNA substrate concentration. Background signal of the RNA-only sample is depicted in gray. Kinetic parameters (Km and Vmax) were calculated for both enzymes using Prism. Data are presented as the mean (+/- SEM) for measurements from two independent experiments ($n = 2$) (**h,i**) IRE1-KR-0P and IRE1-KR-3P digestions of T7 RNAs for PIGQ, BMP4, BCAM, and SNN (**h**); or GBA, WT1, CCDC69, and AIM2 (**i**). (**j**) Detection of the endogenous IRE1α protein by immunofluorescence (red), coupled with detection of the RIDDLE substrate TNFAIP8L1, and negative control substrate PRICKLE2, by RNA fluorescence in situ hybridization (FISH, RNA Scope) (green) in MDA-MB-231 cells treated with Tg (100 nM) for 8 hr or 24 hr as compared to baseline (no Tg). Nuclei are stained with DAPI. (**k)** Percentage of mRNA punctae overlapping with IRE1α punctae for TNAFIP8L1 and PRICKLE2 at each timepoint.

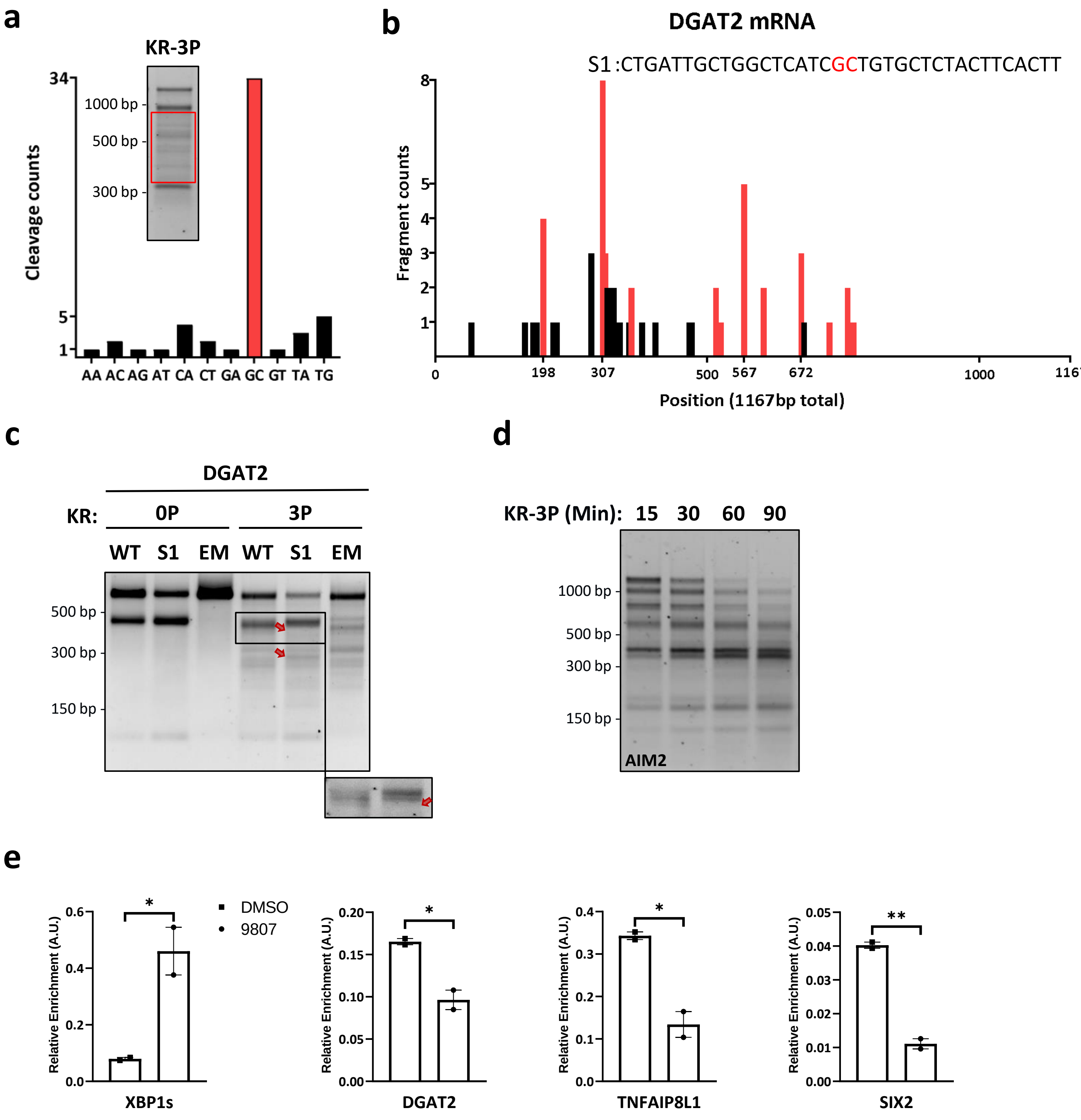
a
KR-3P
34
1000 bp
500 bp
300 bp
Cleavage counts
5
1
AA AC AG AT CA CT GA GC GT TA TG
b
DGAT2 mRNA
S1 :CTGATTGCTGGCTCATCGCTGTGCTCTACTTCACTT
Fragment counts
8
5
4
3
2
1
0
198
307
500
567
672
1000
1167
Position (1167bp total)
c
DGAT2
KR:
0P
3P
WT S1 EM WT S1 EM
500 bp
300 bp
150 bp
d
KR-3P (Min): 15 30 60 90
1000 bp
500 bp
300 bp
150 bp
AIM2
e
DMSO
9807
Relative Enrichment (A.U.)
0.6
0.4
0.2
0.0
XBP1s
0.20
0.15
0.10
0.05
0.00
DGAT2
0.4
0.3
0.2
0.1
0.0
TNFAIP8L1
0.05
0.04
0.03
0.02
0.01
0.00
SIX2
*
**

Supplementary Figure 3. RIDDLE is more promiscuous in substrate recognition, yet non-random. (**a**) Amount of RNA fragments sequenced whose 3’ end leads to the cleaved nt pair designated on the x axis. The first nucleotide in the pair represents the last sequenced nucleotide from the RNA fragment, while the second nucleotide shows the following base in the RNA sequence. Inset: red box indicates the portion of the gel that was extracted for subsequent sequencing. (**b**) Mapping of the last base pair (3’ end) from each individual RNA fragment sequenced within the DGAT2 mRNA. Red bars indicate cleavage sites between a GC nt pair. Black bars indicate non GC cleavage sites. (**c**) RNA digestions of WT DGAT2 and DGAT2 mutated at location S1 or at the stem-loop endomotif (EM). The red arrows indicate change in banding pattern as compared to WT. The zoomed-in section was taken from the same gel as the main image; however, the gel was run longer to enhance band separation. (**d**) Kinetic analysis of IRE1-KR-3P digestion of AIM2 RNA. (**e**) RT-qPCRs analysis comparing RIDD and RIDDLE targets in *IRE1α* WT MDA-MB-231 cells after a 6-hr treatment by cellular IRE1α activator G-9807 (5 µM). n=2 biologically independent experiments. An unpaired t-test was used to calculate p-values. *, $P \leq 0.05$; **, $P \leq 0.01$.

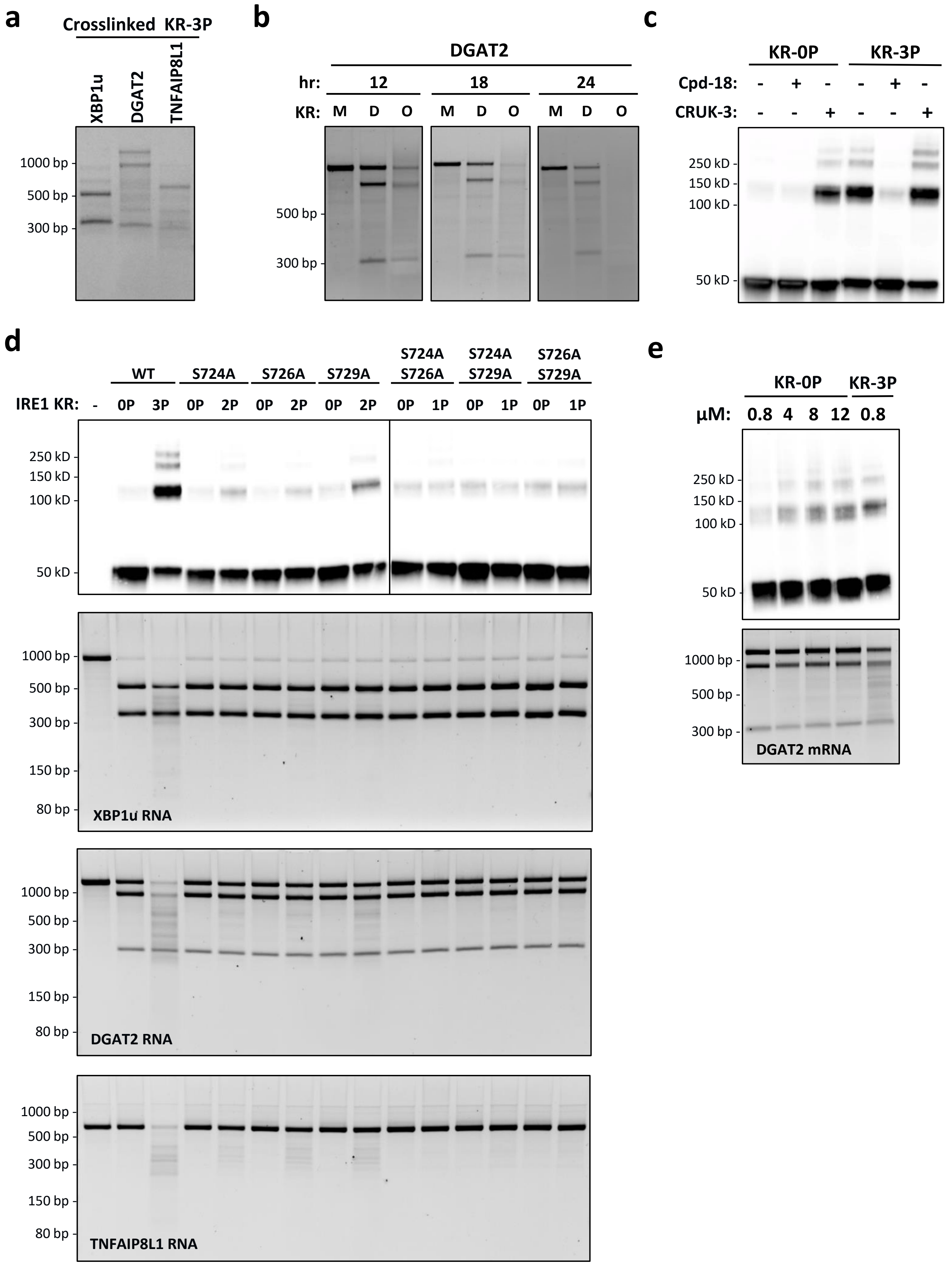
a
Crosslinked KR-3P
XBP1u
DGAT2
TNFAIP8L1
1000 bp
500 bp
300 bp
b
DGAT2
hr:
12
18
24
KR:
M D O
M D O
M D O
500 bp
300 bp
c
KR-0P
KR-3P
Cpd-18:
CRUK-3:
250 kD
150 kD
100 kD
50 kD
d
WT
S724A
S726A
S729A
S724A S726A
S724A S729A
S726A S729A
IRE1 KR:
0P 3P 0P 2P 0P 2P 0P 2P 0P 1P 0P 1P 0P 1P
250 kD
150 kD
100 kD
50 kD
1000 bp
500 bp
300 bp
150 bp
80 bp
XBP1u RNA
DGAT2 RNA
TNFAIP8L1 RNA
e
KR-0P
KR-3P
µM:
0.8 4 8 12 0.8
250 kD
150 kD
100 kD
50 kD
1000 bp
500 bp
300 bp
DGAT2 mRNA

Supplementary Figure 4. Phospho-oligomeric state governs IRE1α's RNase modality. (**a**) IRE1-KR-3P digestion of XBP1u, DGAT2, and TNFAIP8L1 RNA immediately after DSS crosslinking and before fractionation of the protein. (**b**) Digestion of DGAT2 RNA by crosslinked IRE1-KR-3P at the indicated incubation time after protein fractionation. (**c**) DSS crosslinking of IRE1-KR in the presence of Cpd-18 or CRUK-3 (both at 5 µM. (**d**) DSS crosslinking of IRE1-KR WT or IRE1-KR mutated at the indicated activation-loop phosphorylation sites, and corresponding digestion of XBP1u, DGAT2, and TNFAIP8L1 RNA. (**e**) Digestion of DGAT2 RNA by IRE1-KR-0P at increasing concentrations compared to IRE1-KR-3P at standard concentration. Top panel shows DSS crosslinking of IRE1-KR and bottom panel shows RNA digestion.

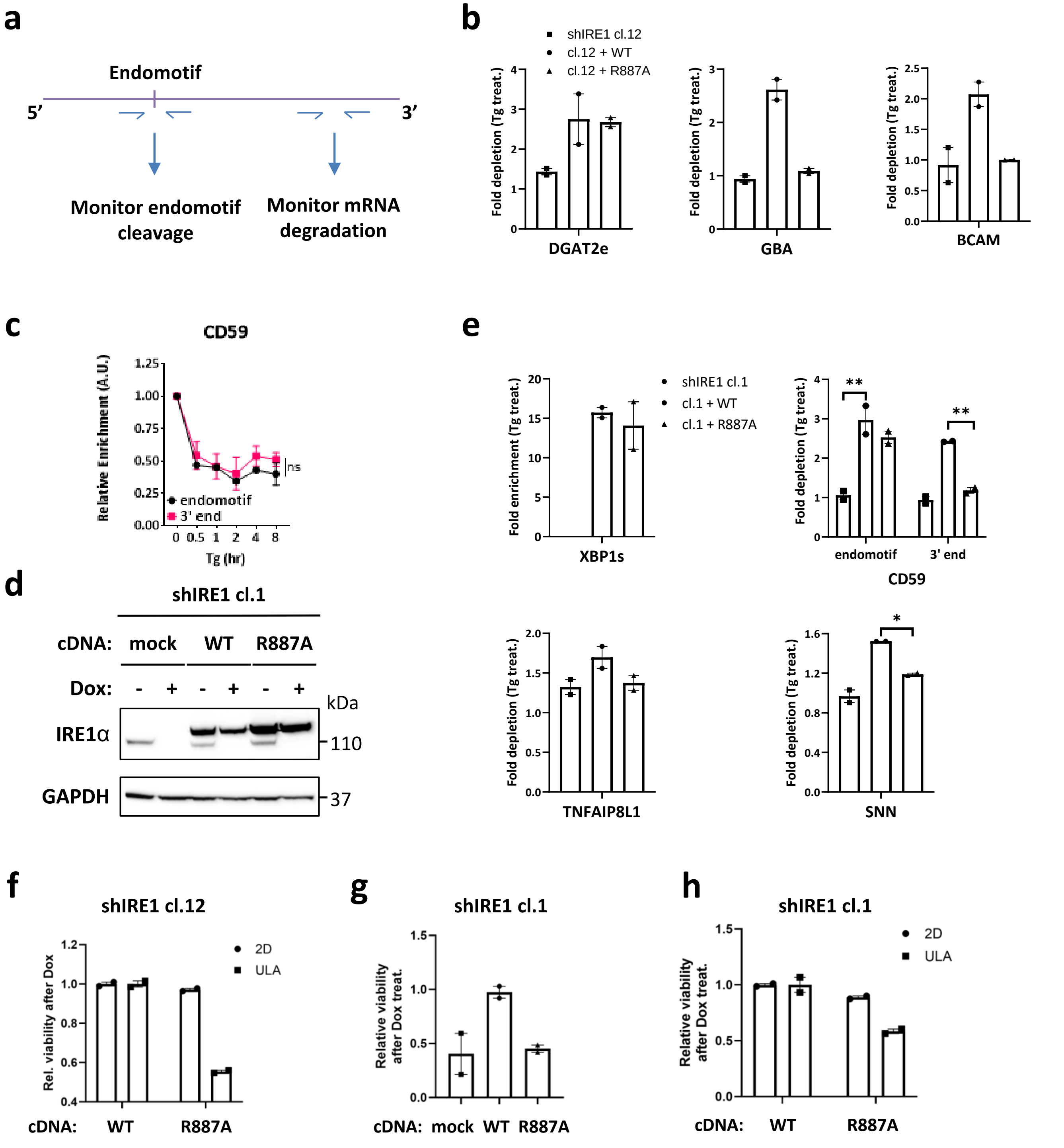

a
Endomotif
5'
3'
Monitor endomotif cleavage
Monitor mRNA degradation
b
shIRE1 cl.12
cl.12 + WT
cl.12 + R887A
Fold depletion (Tg treat.)
DGAT2e
GBA
BCAM
c
CD59
Relative Enrichment (A.U.)
endomotif
3' end
ns
Tg (hr)
d
shIRE1 cl.1
cDNA: mock WT R887A
Dox: - + - + - +
kDa
IRE1α
110
GAPDH
37
e
shIRE1 cl.1
cl.1 + WT
cl.1 + R887A
Fold enrichment (Tg treat.)
XBP1s
Fold depletion (Tg treat.)
endomotif
3' end
CD59
**
**
TNFAIP8L1
SNN
*
f
shIRE1 cl.12
2D
ULA
Rel. viability after Dox
cDNA: WT R887A
g
shIRE1 cl.1
Relative viability after Dox treat.
cDNA: mock WT R887A
h
shIRE1 cl.1
2D
ULA
Relative viability after Dox treat.
cDNA: WT R887A

Supplementary Figure 5. R887A mutant IRE1α displays cellular deficiency in oligomerization and RIDDLE. (**a**) Cartoon depicting the location of the primer pairs used for RT-qPCR analysis of endomotif-directed cleavage vs. 3'-end decay (RIDDLE). (**b**) RT-qPCR analysis of IRE1α RNase targets. DGAT2e was analyzed by primers surrounding the endomotif; GBA and BCAM were analyzed by primers mapping at the 3' end of the transcript. n=2 biologically independent experiments. Data are presented as mean values +/- SEM. (**c**) Kinetic analysis of CD59 depletion comparing RT-qPCR with primers detecting endomotif-directed and 3'-end decay. n=2 biologically independent experiments. Data are presented as mean values +/- SEM. An unpaired t-test was used to calculate p-value. (**d**) Immunoblot analysis of endogenous and ectopic IRE1α variant expression in HCC1806 cells harboring Dox-inducible IRE1α shRNA that were stably transfected with transgenic WT or R887A mutant versions of IRE1α-GFP. (**e**) RT-qPCR analysis of IRE1α RNase targets as described for Fig. 5. n=2 biologically independent experiments. Data are presented as mean values +/- SEM. A 2-way ANOVA test was used to calculate CD59 p-values. An unpaired t-test was used to calculate XBP1s and SNN p-values. (**f**) Analysis of MDA-MB-231 cell viability by Cell-Titer Glo after Dox treatment (0.4 µg/ml) for 7 days on standard flat bottom (2D) or Ultra-Low Attachment plates (ULA). n=2 biologically independent experiments. Data are presented as mean values +/- SEM. (**g**) Analysis of HCC1806 cell viability by Cell-Titer Glo after Dox treatment for 7 days on ULA plates. n=2 biologically independent experiments. Data are presented as mean values +/- SEM. (**h**) Analysis of HCC1806 cell viability by Cell-Titer Glo after Dox treatment for 7 days on standard flat bottom (2D) or ULA plates. n=2 biologically independent experiments. Data are presented as mean values +/- SEM. *, $P \leq 0.05$; **, $P \leq 0.01$.

a

b

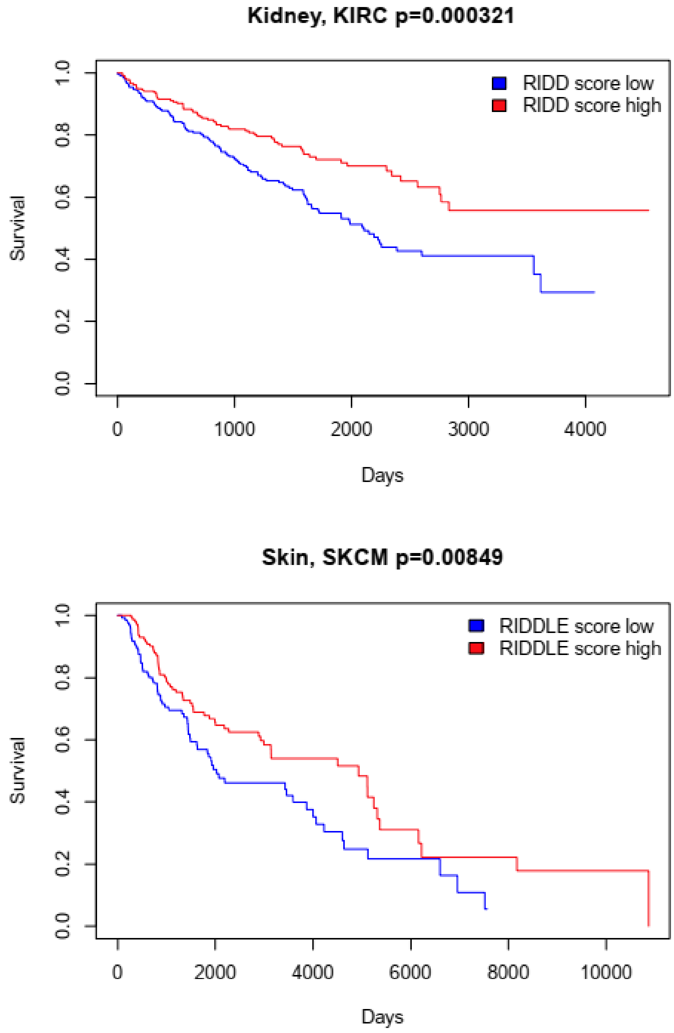

Supplementary Figure 6. Survival plots for cancers displaying significantly better prognosis for patients with lower RIDD scores (a) and lower RIDDLE scores (b) from TCGA. Scores are based on expression levels of genes listed in the first two categories (RIDD) or the last two categories (RIDDLE) of Supplementary Table 4. Survival in these cancers was significantly different ($p < 0.01$). The Cox model was used to calculate p-values.

	Gene name	IRE1-dependent down-regulation (RNAseq)	Fold down-regulation over transcription (RNAseq/GROseq)	Signal sequence
1	*AIM2*	37%	1.67	None
2	*ALDH1A3*	37%	1.25	None
3	*ATF5*	35%	1.58	None
4	*ATP9A*	41%	1.44	None
5	*B3GNT8*	39%	1.89	Type I
6	*BCAM*	79%	1.71	Type I
7	*BLOC1S1*	30%	1.31	None
8	*BMP4*	49%	1.32	Type I
9	*CALHM2*	31%	1.33	None
10	*CCDC69*	33%	1.41	None
11	*CD59*	42%	1.65	Type I
12	*CDKN1C*	47%	1.65	Type I
13	*CEP162*	41%	1.93	None
14	*CFAP45*	32%	1.96	None
15	*CHST4*	45%	3.29	None
16	*CTSO*	45%	1.33	Type I
17	*DGAT2*	81%	4.07	Type II
18	*DSEL*	37%	1.30	Type I
19	*ERCC6*	30%	1.28	None
20	*FAM117B*	30%	1.43	None
21	*FAM63A*	53%	1.29	None
22	*FILIP1L*	50%	1.31	None
23	*FITM2*	31%	2.18	Type II
24	*GATSL2*	29%	1.51	None
25	*GBA*	41%	1.38	Type I
26	*GJD3*	32%	1.36	Type I
27	*GPC1*	33%	1.34	Type I
28	*HAPLN3*	40%	2.01	Type I
29	*HIP1*	40%	1.45	None
30	*IL31RA*	41%	1.27	Type I
31	*KIAA1467*	33%	1.58	None
32	*LRRN4*	32%	3.05	Type I
33	*MAP3K5*	31%	1.30	None
34	*MAST4*	35%	1.30	None
35	*METTL7A*	41%	2.15	None
36	*MFAP2*	37%	1.90	Type I
37	*MILR1*	42%	2.12	Type I
38	*OAS2*	41%	1.32	None
39	*PIGQ*	33%	1.38	None
40	*PROS1*	38%	4.38	Type I
41	*RGS7*	35%	1.39	None
42	*RNF213*	33%	1.41	None
43	*SIX2*	37%	1.60	None
44	*SNN*	32%	1.78	Type II
45	*SRXN1*	36%	1.24	None
46	*SUOX*	32%	1.32	None
47	*TGOLN2*	56%	2.43	Type I
48	*TLR2*	43%	1.68	Type I
49	*TMEM19*	37%	1.86	None
50	*TNFAIP8L1*	30%	4.04	None
51	*TRIM16L*	32%	1.51	None
52	*TRIM62*	30%	1.34	None
53	*WT1*	50%	1.49	None
54	*ZFHX4*	36%	1.27	None

Supplementary Table 1. IRE1α-dependent down-regulated targets in MDA-MB-231 cells. mRNA transcripts down-regulated upon ER stress in an IRE1α-dependent and transcription-independent manner. The percentage of IRE1α-dependent down-regulation upon Tg treatment measured by RNAseq is represented in column 3. Fold depletion of total RNA (RNAseq Log_2(FC)WT) over transcriptional down-regulation (GROseq Log_2(FC)WT) is indicated in column 4. Analysis for the presence of a signal peptide (Type I) or signal anchor (Type II) sequence is indicated in the last column.

Molecular and cellular functions	p-value range	Number of genes
Cell death and survival	1.41e-02 – 6.16e-05	11
Cell signaling	1.18e-02 – 7.77e-05	4
Post-translational modification	8.91e-03 – 7.77e-05	6
Cell morphology	1.41e-02 - 2.44e-04	10
Cell cycle	1.41e-02 - 3.75e-04	8

Supplementary Table 2. Ingenuity Pathway Analysis (IPA) of IRE1α's mRNA decay targets in MDA-MB-231 cells. Top 5 enriched pathways in the Molecular and Cellular Functions category according to the IPA software, along with their p-value range and the number of targets in that group. Fisher's Exact Test was used for p-value calculations.

GO biological process	p-value	Number of genes
Response to endoplasmic reticulum stress (GO:0034976)	1.87E-31	46
Endoplasmic reticulum to Golgi vesicle-mediated transport (GO:0006888)	2.89E-26	37
IRE1-mediated unfolded protein response (GO:0036498)	6.73E-17	18
Cellular response to unfolded protein (GO:0034620)	3.78E-16	23
Protein N-linked glycosylation via asparagine (GO:0018279)	3.80E-15	14
ERAD pathway (GO:0036503)	8.90E-15	19
COPII-coated vesicle cargo loading (GO:0090110)	1.46E-12	10
Protein folding in endoplasmic reticulum (GO:0034975)	3.74E-06	5
Somatostatin signaling pathway (GO:0038170)	7.45E-06	4
SRP-dependent cotranslational protein targeting to membrane (GO:0006614)	7.50E-06	10
COPI coating of Golgi vesicle (GO:0048205)	1.23E-05	4
Protein K69-linked ufmylation (GO:1990592)	2.11E-04	3
Mannose trimming involved in glycoprotein ERAD pathway (GO:1904382)	2.11E-04	3

Supplementary Table 3. GO term analysis of mRNA targets up-regulated in IRE1α-dependent manner after Tg treatment in MDA-MB-231 cells based on the GROseq dataset. Most enriched GO terms in the Biological process category, along with their p-value and the number of targets in that group. Fisher's Exact Test was used for p-value calculations.

Gene	Transcript	Pos	Structure	Loop	Stem	Energy	FL	FS	Type
**Exact consensus sequence**									
MAST4	XM_011543384.2	560	((((((..(((((CUGCAG)))))))))))	Exact	11	−11.60			
PROS1	NM_000313.4	2820	(((((((((CUGCAGU)))))))))	Exact	9	−9.40			
DGAT2	BC015234.1	260	((((.(((((CUGCAGU))))).))))	Exact	9	−9.00			0P
BLOC1S1	NM_001487.2	360	((((((CUGCAGU))))))	Exact	6	−8.70			0P
RNF213	NM_001256071.3	9055	((((((((CUGCAG))))))))	Exact	8	−7.30			
ERCC6	NM_001346440.2	20483	(((((((.(CUGCAG).)))))))	Exact	8	−5.70			
SUOX	XM_017019907.2	5148	((((((CUGCAGAC))))))	Exact	6	−4.90			
HAPLN3	XR_931756.3	1184	((((((((CUGCAGGG))..))))))	Exact	8	−4.80			
ATF5	NM_012068.6	249	((((.((CUGCAGC)).))))	Exact	6	−3.80			
IL31RA	NM_139017.7	5705	((((CUGCAG))))	Exact	4	−3.20			
TGOLN2	NM_006464.3	390	((((CUGCAGA))))	Exact	4	−2.90			0P
GBA	NM_000157.4	612	(.(((CUGCAGUU))).)	Exact	4	−1.70			0P
CD59	NM_203331.2	78	((((CUGCAGU))))	Exact	4	−1.10			0P
MILR1	XM_024450707.1	1433	((((((.(CUGCAGU)))))))	Exact	7	−0.70			
TRIM62	NM_018207.3	1082	((((CUGCAGC))))	Exact	4	−0.60			
CASTOR2	NM_001145064.3	7422	((((CUGCAGAU))))	Exact	4	−0.30			
LRRN4	NM_152611.5	2378	(((CUGCAGC)))	Exact	3	−0.10			
ZFHX4	NM_024721.5	4348	(((CUGCAGCC)))	Exact	3	0.10			
FAM234B	NM_020853.2	1361	(((((CUGCAGAU).))))	Exact	5	1.10			
PIGQ	NM_004204.3	972	((.(((CUGCAG))).))	Exact	5	2.60			0P
CEP162	XM_017010483.2	605	(((((((CUGCAGG).))))))	Exact	7	3.70			
BMP4	NM_130850.3	186	((((..(CUGCAG)))))	Exact	5	3.90			0P
**Variant consensus sequence**									
WT1	BC032861.2	120	(((((((((CaGCAGG))))).))))	Var2	9	−9.70			0P
FITM2	NM_001080472.4	327	((((((((CUGCgGC))))))))	Var5	8	−7.70			
TMEM19	NM_018279.4	1581	((.(((((CUGCuGC)))))))	Var5	7	−5.30			
TRIM16L	NM_001037330.3	967	(((((.(((CaGCAGU))).)))))	Var2	8	−5.20			
GPC1	NM_002081.3	1200	(((((((CgGCAGC))).))))	Var2	7	−2.70			
B3GNT8	NM_198540.2	1417	((((CUGCgGG))))	Var5	4	−2.00			
MAP3K5	NM_005923.4	2467	((((((CaGCAGA).)))))	Var2	6	−1.70			
DSEL	NM_032160.3	4897	(((((((CaGCAGA).))))))	Var2	7	0.00			
**Non-canonical stem–loop**									
ATP9A	NM_006045.3	621	((.(((((CUGCgG))))).))	Var5	7	−5.60	×		
BCAM	NM_005581.3	293	((((CcGCAGU))))	Var2	4	−4.40		×	3P
CALHM2	NM_015916.5	1824	(((CaGCAG)))	Var2	3	0.50	×	×	
CCDC69	NM_015621.2	786	(((CaGCAG)))	Var2	3	−0.10	×	×	3P
CDKN1C	NM_000076.2	1340	((((CcGCAGAG))))	Var2	4	−3.20	×	×	
CTSO	NM_001334.3	308	((((CaGCAGA))))	Var2	4	0.70		×	
FAM117B	NM_173511.4	448	(((CcGCAGGC)))	Var2	3	−2.30	×	×	
FILIP1L	NM_001387850.1	5557	((((CaGCAGU))))	Var2	4	−2.70		×	
HIP1	NM_005338.7	296	((((.(((CUGCuGGA))).))))	Var5	7	−6.20	×		
MFAP2	NM_002403.3	290	((((.(CUGCcG).))))	Var5	5	0.00	×		3P
RGS7	XM_017002011.2	1695	(((CUGCcGG)))	Var5	3	2.80		×	
SIX2	NM_016932.4	720	(((CUGCcGU)))	Var5	3	−1.10		×	3P
SNN	NM_003498.5	108	(((((.(CUGCgGCU).)))))	Var5	6	−3.50	×		3P
SRXN1	NM_080725.3	174	((((((((CUGCcGUC)).))))))	Var5	8	−6.70	×		
TNFAIP8L1	NM_152362.1	219	(((CUGCuGCG)))	Var5	3	3.60	×	×	3P
**Consensus sequence plus stem–loop not found**									
AIM2	NA								3P
ALDH1A3	NA								
CFAP45	NA								3P
CHST4	NA								
GJD3	NA								
METTL7A	NA								
MINDY1	NA								
OAS2	NA								3P
TLR2	NA								

Supplementary Table 4. Canonical stem-loop endomotif analysis of RIDD targets from Table S1 using the gRIDD algorithm. mRNA transcripts down-regulated upon ER stress in an IRE1α-dependent and transcription-independent manner were analyzed for the presence and robustness of a canonical XBP1-like stem-loop endomotif, using a newly developed algorithm termed gRIDD (see supplementary methods for detailed description of gRIDD). Columns 1 and 2 indicate gene names and transcript references. Column 3 indicates mRNA nucleotide position of the G within the GC cleavage site. Column 4 illustrates the loop sequence and stem topology of the endomotif, where a parenthesis indicates a nucleotide that is paired with a complementary one on the opposite leg of the stem, and a dot indicates an unpaired nucleotide. Lowercase letters indicate variation to the consensus loop sequence at position 2 or 5, indicated in column 5 as Var2 and Var5 respectively. Column 6 reports the number of base pairs at the stem. Column 7 indicates the free energy (kcal/mol) for the stem-loop structure represented in column 4. Columns 8 and 9 report failure to meet gRIDD criteria due to excessive loop-length (FL) and/or disrupted base pairing at the stem (FS). Column 10 indicates empirical results for cleavage by IRE1-KR-0P (0P) or IRE1-KR-3P (3P). All transcripts cleaved by IRE1-KR-0P were also cleaved by IRE1-KR-3P. In each case, the best possible stem-loop endomotif is displayed. The first and second categories include mRNAs with stem-loop endomotifs that meet gRIDD criteria (RIDD modality). The third and fourth categories include mRNAs wherein the best possible endomotif nevertheless fails to meet gRIDD criteria or no stem-loop endomotif is found (RIDDLE modality). Within each category, transcripts are ranked by free energy.

Site	Alignment
TNFAIP8L1 S1	gaagcugggacugcugcugcguggggaccagcugggcggugaggagcuggcgCUGCUGcggcgcuuccgccaccgggcgcgcugccuggccaugacggccgucagcuucc<br>((((((..(((.(((((((.((.(((....(((.((.((((.(((((.(.((......)).))))))))))).)).)))......))).)))).).)))).))))))))).<br><br>number of consecutive pairs is only 2 (3 required by gRIDD)
TNFAIP8L1 S2	uucacgcgcagccgcaaggaggcccagaagaugcucaagaaccuggucaaggUGGCCCugaagcugggacugcugcugcguggggaccagcugggcggugaggagcuggc<br>(((.((((((((....((.((.(((((.......(((....(((.....)))......)))..))))).)).)))))))))).)))((((((...........)))))).<br><br>stem-loop structure is not found around the motif (as per gRIDD)
DGAT2 S1	1) cauccucauguacauauucugcacugauugcuggcucaUCGCUGugcucuacuucacuuggcugguguuugacuggaacaca<br>.....(((((((.......)))).)))..(((((((((......))............)))))))((((.....))))...<br><br>number of consecutive pairs is only 2 (3 required by gRIDD)<br><br>2) cauccucauguacauauucugcacugauugcuggcucaUCGCUGUgcucuacuucacuuggcugguguuugacuggaacaca<br>.....(((((((.......)))).)))..((((((.((.......(.....)......))))))))((((.....))))...<br><br>stem-loop structure is not found around the motif (as per gRIDD)

Supplementary Table 5. Sequences surrounding GC cleavage sites of TNFAIP8L1 and DGAT2. The top row shows the nucleotide sequence and the bottom row illustrates loop sequence and stem topology as in Supplementary Table 4.

miRNA	Gencode h38	Consensus sequence and stem-loop by gRIDD
miR-17	ENST00000385012.1	Not found
miR-34a	ENST00000385130.1	Not found
miR-96	ENST0000036228.1	Not found
miR-125b1	ENST00000385236.1	Not found
miR-125b2	ENST00000385128.1	Not found
miR-200a	ENST00000384875.3	Not found
miR-200b	ENST00000384997.3	Not found
miR-200c	ENST00000384980.3	Not found

Supplementary Table 6. Results of miRNA sequences analyses by the gRIDD program. The miRNA sequences listed were analyzed by gRIDD as in Supplementary Table 4.

COSMIC	Mutation	Histology	FATHMM Score
COSM8887124	R887H	Endometrioid Carcinoma	0.99
COSM7488098	R887C	Glioma (Astrocytoma Grade IV)	0.96

Supplementary Table 7. COSMIC identification of IRE1 R887 mutations in cancer patients. cancer.sanger.ac.uk/cosmic/gene/analysis?ln=ERN1

Supplementary information

Probe Names	Vendor	Catalog Number
RNAScope 2.5 LS Human-PRICKLE2	ACD Biosystems	565031
RNAScope 2.5 LS Human-TNFAIP8L1	ACD Biosystems	19255A
RNAScope 2.5 LS Human-PPIB	ACD Biosystems	313908
RNAScope 2.5 LS DapB	ACD Biosystems	312038

Antibody	Vendor	Clone	Cat#/Lot	Concentration
Rat anti-hIRE1α	GNE	GN35-18.ratIgG2b	PUR141700	1 µg/ml
mono-Rat IgG2b	BD Pharmingen	A95-1	553986	1 µg/ml

Procedure	Detection Used	Vendor	Catalog Number
ISH	Opal-570	Perkin Elmer	FP1488001KT
ICC	Opal-690	Perkin Elmer	FP1488001KT

Supplementary Table 8. RNAScope probes, Antibody & Detections used in this study

CD59	Endomotif	f:GCAGTCAGCGTTGGGTTAG	r:CTCGTCCTGGCTGTCTTCT
	3' end	f:GCCTGCAGTGCTACAACT	r:CAATGCTCAAACTTCCAACACT
TGOLN2	Endomotif	f:GCCTCCGCACTCGACTT	r:GCCTGGAGGCTCTACCAA
	3' end	f:TCACAACAAGCGGAAGATCA	r:AGAGGAATATACCATTCTGTTAGGAC
DGAT2e	Endomotif	f:TGGGTCCTGTCCTTCCTT	r:CGATGAGCCAGCAATCAGT

Supplementary Table 9. Custom designed RT-qPCR primers

**IRE1α KR**	**% phosphorylation**		
	**S724**	**S726**	**S729**
**WT 3P**	82	Yes*	52
**S724A 2P**	-	>90	>90
**S726A 2P**	90	-	71
**S729A 2P**	82	Yes*	-
**S724A-S726A 1P**	-	-	97
**S726A-S729A 1P**	89	-	-
**S724A-S729A 1P**	-	Yes*	-

Yes* denotes observed S726 phosphorylation for which quantification was not possible due to the presence of a miscleaved peptide containing phosphorylated S726

Supplementary Table 10. Phosphorylation site mapping

Supplemental method description of gRIDD algorithm

Program and datasets

Our study empirically identified several examples of RIDD substrates, which we used to help refine the canonical pattern for RIDD cleavage sites. To this end, we used RNA transcripts that were cleaved by IREKR-0P as positive examples to be identified by our prediction rules, and transcripts cleaved only by IRE1-KR-3P as negative examples to be excluded.

To implement the prediction rules, we developed a computational program, called gRIDD (Genentech RIDD predictor), to scan a given transcript and report potential RIDD cleavage sites within it. The program first looks for candidate consensus sequences in the transcript. We used existing literature, which suggests that RIDD substrates have the consensus sequence CNGCNG, with four nucleotides highly conserved. The most common, or exact, consensus sequence has been observed to be CUGCAG. We allow for one mismatch from the exact consensus sequence, meaning that either position 2 or position 5 is permitted to deviate, yielding the variant consensus sequences CxGCAG or CUGCxG, respectively, where x indicates a variation from the exact consensus sequence.

Canonical RIDD cleavage sites are believed to lie within a stem– loop structure, determined by the nucleotide content surrounding the consensus sequence. We therefore required the consensus sequence to reside within a loop of 6, 7, or 8 bp, where the nucleotides immediately outside the loop were complementary, meaning an A–U, G–C, or G–U pair. For a loop with 7 bp, we required the consensus sequence to be at the 5' end of the loop, leaving one additional nucleotide at the 3' section of the 6nt consensus sequence. Likewise, for a loop with 8 bp, we required the consensus sequence to be at the 5' end of the loop.

For each candidate consensus sequence with a potential loop, our program then uses the RNAfold program to determine the secondary RNA structure surrounding the loop, providing that program with the constraint that the nucleotides immediately surrounding the loop be base-paired. To ensure that a stem–loop can exist in its global context, we provided RNAfold with a neighborhood of 55–60 nucleotides surrounding

each candidate consensus sequence, testing each value from 60 down to 55, ending if and when a stem–loop structure surrounding the consensus sequence position was predicted. We used a range of nucleotides for the neighborhood length since we were uncertain about the boundaries where critical nucleotides at the end might have affected the global secondary structure.

The result of RNAfold is a secondary structure with minimum free energy, where bases are paired using the "(" and ")" symbols. Our program then analyzes the stem structure to require that it contain at least 3 consecutive base pairs. Each stem is further extended as far distally as possible, allowing for either a single mismatch at the same position in both legs of the stem, or a single bulge of up to 3 nucleotides. The total number of base pairs in the extended stem is used as a factor to discriminate RIDD sites from non-RIDD sites.

We applied the gRIDD program to the transcripts analyzed in our in-vitro experiments, which used a specified set of coding regions, from the start codon to the stop codon. The results of the program are shown in Table 1 below, with the positive examples used shown in black, and the negative ones in red. RNAs that failed to show cleavage by IRE1-KR-0P could potentially contain multiple sites, each of which constitutes a negative example of a RIDD site.

Table 1: Positive (black) and negative (red) examples from this study

Gene	Transcript	Pos	Structure	Loop	Stem
**Observed 0P-type cleavage**					
DGAT2	BC015234.1	260	((((.(((((CUGCAGU))))).))))	Exact	9
XBP1#1	NM_005080.3	519	(((((((CUGCAGC)))))))	Exact	7
BLOC1S1	NM_001487.2	360	((((((CUGCAGU))))))	Exact	6
TGOLN2	NM_006464.3	390	((((CUGCAGA))))	Exact	4
CD59	NM_203331.2	78	((((CUGCAGU))))	Exact	4
PIGQ	NM_004204.3	972	((.(((CUGCAG))).))	Exact	5
BMP4	NM_130850.3	186	((((..(CUGCAG)))))	Exact	5
XBP1#2	NM_005080.3	484	(((((CUGCuGA)))))	Var5	5
**Observed 3P-type cleavage**					
BCAM#1	NM_005581.3	48	((((CUGCuG))))	Var5	4
BCAM#2	NM_005581.3	293	((((CcGCAGU))))	Var2	4
MFAP2#1	NM_002403.3	290	((((.(CUGCcG).))))	Var5	5
MFAP2#2	NM_002403.3	494	(((CaGCAGCG)))	Var2	3
SIX2#1	NM_016932.4	720	(((CUGCcGU)))	Var5	3
SIX2#2	NM_016932.4	66	((((((CaGCAGGG)))).))	Var2	6
SNN	NM_003498.5	108	(((((.(CUGCgGCU).)))))	Var5	6
TNFAIP8L1	NM_152362.1	219	(((CUGCuGCG)))	Var5	3

We also applied the gRIDD program to positive examples of RIDD substrates from the literature, namely, a set of 13 human genes found to be RIDD substrates by Oikawa et al., 2010 (Table 2 below), and a set of 16 additional mammalian genes (4 human and 12 mouse) summarized from the literature by Maurel et al., 2014 (Table 3 below). For the Oikawa dataset, we used the full-length transcript from historical RefSeq archives that matched the transcript length given in their paper. For the Maurel genes, we analyzed all current transcript isoforms in RefSeq corresponding to the given gene. We considered examples from the literature as positive examples, although it is possible that other experiments may have confounded different cleavage modalities.

Table 2: Positive examples from Oikawa et al., 2010. Genes are colored in blue for subsequent reference. Ref indicates the cleavage site from the original paper.

Gene	Transcript	Pos	Ref	Structure	Loop	Stem
CCT3#1	NM_001008883.1	1726		(((CUGCAGA)))	Exact	3
CCT3#2	NM_001008883.1	1754		((((((((((CgGCAGU))).)))))))	Var2	10
FDFT1	NM_004462.3	1073		((((.(CaGCAG)))))	Var2	5
GEMIN5	NM_015465.2	4862	4861	(((((((((((CUGCAGA))))))))..)))	Exact	11
IRAK1	NM_001025242.1	2041		((((((CUGCAGCU))))))	Exact	6
MKRN2#1[a]	NM_014160.3	237		((((((CUGCAGC))))))	Exact	6
MKRN2#2	NM_014160.3	2249	2251	((((CaGCAGU))))	Var2	4
MPC1[b]	NM_016098.1	712		(((CUGCAGUA)))	Exact	3
PDK2	NM_002611.3	1978	1978	(((((CUGCAGU)))))	Exact	5
PEPD	NM_000285.2	1455	1455	((((((((((.(CUGCAGC).))))))))))	Exact	11
PMF1	NM_007221.2	756		(((((CaGCAGU).))))	Var2	5
PPP2R1A	NM_014225.3	1748	1748	((((CUGCAGA))))	Exact	4
PRKCD	NM_006254.3	1736	1736	((((((((CUGCAGU).)))))))	Exact	8
RUVBL1	NM_003707.1	1536	1536	((((CUGCcGU))))	Var5	4
YWHAQ	NM_006826.2	994[c]	1168[d]	((((((.(CUGCAGC).))))))	Exact	7

Induction of predictive rules

We derived predictive rules for RIDD sites based on the results of our program, which reported the loop size and stem lengths for each candidate consensus sequence. We divided our analysis based on the three possible consensus patterns: exact (CUGCAG), variant 5 (CUGCxG), and variant 2 (CxGCAG). We found that the majority of known positive RIDD sites had an exact consensus sequence, followed by a smaller number with variant 5, and a smaller number with variant 2.

For the set of positive and negative examples with an exact consensus sequence, we categorized sites by their loop size and stem lengths (Table 4 below). Genes are colored according to their source, using the same colors as in Tables 1–3. Human genes are shown in all capital letters, while mouse genes are shown with the first letter capitalized, according to standard convention. The most prevalent loop size was 7 nt, with some observed to have loop sizes of 6 or 8 nt. In all cases, the positive examples had stem lengths of 3 or more bp. Therefore, we can capture known RIDD sites around exact consensus sequences by allowing for loop sizes of 6, 7, or 8 bp and requiring 3 or more base pairs in their extended stems.

Table 3: Positive examples from Maurel et al., 2014. Genes are colored in light blue for subsequent reference. Genes from Oikawa et al., 2010, are excluded, as are genes that have no entry as an NM or XM transcript in RefSeq: 28S RNA, MIR17, and μs.

Gene[a]	Transcript	Pos	Structure	Loop	Stem
Angptl3	NM_013913.4	204	((((.(CUGCAGCU)))))	Exact	5
Bloc1s1	NM_015740.3	449	((((((CUGCAGU)))).))	Exact	6
Col6a1	NM_009933.4	1945	(((((((CUGCuGU)))))))	Var5	7
Cyp2e1	NM_021282.3	1326	(((((((CUGCAGG)))))))	Exact	7
ERN1	XM_017024347.2	3086	((((((CUGCAGG))))))	Exact	6
GPC3	NM_004484.4	1374	(((((..(CUGCAGCC))))))	Exact	6
Galnt2	NM_139272.2	589	(((((((CUGCgGA))).))))	Var5	7
Hgsnat#1	NM_029884.1	159	(((((..((CUGCuGC)))))))	Var5	7
Hgsnat#2	NM_029884.1	752	(((((((.(CaGCAGA).)))))))	Var2	8
Itgb2	NM_008404.5	1250	((((((CUGCAGU)).))))	Exact	6
PER1	NM_002616.3	18	(((((CUGCgGG)).)))	Var5	5
Pdgfrb	NM_008809.2	4940	(((CaGCAGC)))	Var2	3
Pmp22	NM_001302257.1	169	(((CUGCAGGC)))	Exact	3
RTN4	NM_020532.5	2663	(((CUGCAGUU)))	Exact	3
Scara3	NM_172604.3	219	(((.(((((CUGCAGA))))).)))	Exact	8
Tapbp	NM_009318.2	591	(.((((CUGCuGG)))))	Var5	5

[a] No stem–loop structures with an endomotif were found for Ces1 (Ces1a, Ces1b, or Ces1c), Cyp1a2, or PDIA4.

Table 4: Exact consensus sequences: CUGCAG

Loop size	Stem ≥ 3	Stem < 3
6	*BMP4*(5), *PIGQ*(5)	
7	*GEMIN5*(11), *PEPD*(11), *DGAT2*(9), *PRKCD*(8), *Scara3*(8), *XBP1#1*(7), *YWHAQ*(7), *Cyp2e1*(7), *BLOC1S1*(6), *Bloc1s1*(6), *ERN1*(6), *Itgb2*(6), *MKRN2#1*(6), *PDK2*(5), *CD59*(4), *TGOLN2*(4), *PPP2R1A*(4)	
8	*GPC3*(6), *IRAK1*(6), *Angptl3*(5), *MPC1*(3), *Pmp22*(3), *RTN4*(3)	

Sites with variant 5 consensus sequences are categorized similarly in Table 5 below. Here, we have a negative example from our study (*SIX2*, site #1) that suggests that for a loop size of 7 nt, 4 or more bp are required in the stem. Other negative examples from our study suggest that, even with stems of 4 or more bp, loop sizes of 6 or 8 bp are not substrates for 0P- type cleavage. Therefore, it appears that the predictive rules for variant 5 consensus sequences allow for only a loop size of 7 nt and requires 4 or more base pairs in their extended stems.

Table 5: Variant 5 consensus sequences: CUGCxG

Loop size	Stem ≥ 4	Stem < 4
6	*MFAP2#1*(5), *BCAM#1*(4)	
7	*Col6a1*(7), *Galnt2*(7), *Hgsnat#1*(7), *XBP1#2*(5), *PER1*(5), *Tapbp*(5), *RUVBL1*(4)	*SIX2#1*(3)
8	*SNN*(6)	*TNFAIP8L1*(3)

Finally, for sites with variant 2 consensus sequences, Table 6 below shows positive examples from our study and the literature for a loop size of 7 nt with stem lengths of 5 or more bp. *BCAM* site #2 suggests that a stem length of 4 bp is not sufficient when a variant 2 consensus sequence is present, and *SIX2* site #2 also suggests that a loop size of 8 nt is not a substrate for IRE1-KR-0P type cleavage with a variant 2 consensus sequence. The example *FDFT1* from the literature can be accounted for by allowing a loop size of 6 nt with a variant 2 consensus sequence. However, we do not wish to implement a rule for a single example from the literature. Therefore, a predictive rule for variant 2 consensus sequences appears to allow only for a loop size of 7 nt and requires 5 or more base pairs in their extended stem.

Table 6: Variant 2 consensus sequences: CxGCAG

Loop size	Stem ≥ 5	Stem < 5
6	*FDFT1*(5)	
7	*CCT3*(10), *Hgsnat#2*(8), *PMF1*(5)	*BCAM#2*(4), *MKRN2#2*(4), *Pdfgrb*(3)
8	*SIX2#2*(6)	*MFAP2#2*(3)

Results

Our predictive rules are able to predict all positive examples and to exclude all negative examples from Table 1. In addition, our program can predict 12 of the 13 genes reported by Oikawa et al., 2010, missing *FDFT1* and showing a different cleavage site for *MKRN2*. Our program identifies 12 of the 16 additional mammalian sites reported by Maurel et al., 2014, missing *Pdgfrb*, as well as *Ces1*, *Cyp1a2*, and *PDIA4*, for which no valid consensus sequences with a stem–loop structure was found in the available RefSeq transcripts. To test our rules further, we selected four additional genes with evidence of cleavage in the presence of *IRE1*: *AIM2*, *CCDC69*, *GBA*, and *WT1*. The predictions of our program and experimental results are shown in Table 7 below. In all four cases, experimental results were consistent with our predictive rules.

Table 7: Test cases

Gene	Transcript	Pos	Structure	Loop	Stem	Pred	Obs
AIM2	NA						3P
CCDC69	NM 015621.2	786	(((CaGCAG)))	Var2	3		3P
GBA	NM_000157.4	612	(.(((CUGCAGUU))).)	Exact	4	0P	0P
WT1	BC032861.2	120	(((((((((CaGCAGG))))).))))	Var2	9	0P	0P

Our rules could be made more sophisticated by using quantitative criteria, such as the minimum free energy predicted for the stem–loop structures. However, it is not clear whether minimum free energy is the most appropriate criterion; alternative stem–loop conformations could suffice, even if not at the minimum but still highly probable. Nevertheless, the number of base pairs in the stem serves as a proxy for energy calculations, with more base pairs helping to stabilize the stem– loop structure. Other considerations of secondary structure may also play a role, such as the presence of an additional hairpin loop upstream of the RIDD cleavage site. Such hairpins have been

found to affect ribosomal processing, resulting in translational stalling. However, it is not clear if such hairpins would also affect in vitro cleavage.

Our analysis suggests that a tradeoff exists between the consensus sequence and the stem–loop structure, with an exact consensus sequence allowing for variable loop sizes and shorter stems, while the variant 2 and variant 5 consensus sequences require a loop size of 7 nt and greater stem lengths. This tradeoff suggests that RIDD cleavage depends on both the consensus sequence and the stem–loop structure as interrelated factors.
