# Supplementary material for: Decoding non-canonical mRNA decay by the endoplasmic-reticulum stress sensor IRE1α
Source: Nat Commun. 2021 Dec 15;12:7310. doi: 10.1038/s41467-021-27597-7 (PMC8674358; doi:10.1038/s41467-021-27597-7)
Supplement: Supplementary file 2 — Description of Additional Supplementary Files [file 41467_2021_27597_MOESM2_ESM.pdf]

Description of Additional Supplementary Files

File Name: Supplementary Data 1
Description: RNA Fragments – SANGER sequences for TNFAIP8L1 and DGAT2

File Name: Supplementary Software 1
Description: The gRIDD program
