# Supplementary material for: Decoding non-canonical mRNA decay by the endoplasmic-reticulum stress sensor IRE1α
Source: Nat Commun. 2021 Dec 15;12:7310. doi: 10.1038/s41467-021-27597-7 (PMC8674358; doi:10.1038/s41467-021-27597-7)
Supplement: Supplementary file 3 — Supplementary Dataset 1 [file 41467_2021_27597_MOESM3_ESM.zip › RNA Fragments - Sanger sequences.docx]

**TNFAIP8L1 Fragment sequences**

>Primer sequence

ATGGACACCTTCAGCACCAAG

>D97

ATGGACACCTTCAGCACCNAGAGCCTGGCTCTGCAGGCGCAGAAGAAGCTCCTGAGTAAGATGGCGTCCAAGGCAGTGGTGGCCGTGCTGGTGGATGACACCAGCAGTGAGGTGCTGGATGAGCTGTACCGCGCCACCAGGGAGTTCACGCGCAGCCGCAAGGAGGCCCATAAGATGCTCAAGAACCTGGTCAAGGTGGCCCTGAAGCTGGGACTGCTGCTGCGTGGGGACCAGCTGGGCGGTGAGGAGCTGGCGCTG

>D98

ATGGACACCTTCAGCACCAAGAGCCTGGCTCTGCAGGCGCAGAAGAAGCTCCTGAGTAAGATGGCGTCCAAGGCAGTGGTGGCCGTGCTGGTGGATGACACCAGCAGTGAGGTGCTGGATGAGCTGTACCGCGCCACCAGGGAGTTCACGCGCAGCCGCAAGGAGGCCCAGAAGATGCTCAAGAACCTGGTCAAGGTGGCCCTGAAGCTGGGACTGCTGCTGCGTGGGGACCAGCTGGGCGGTGAGGAGCTGGCGCTG

>D99

ATGGACACCTTCAGCACCAAGAGCCTGGCTCTGCAGGCGCAGAAGAAGCTCCTGAGTAAGATGGCGTCCAAGGCAGTGGTGGCCGTGCTGGTGGATGACACCCGCGACGAGGTGCTGGATGACCTGTACCCCCCCCCCACGGAGTTCACCCGCAGCCGCATGGAGGCCCAGAACATGCTCAAAAACCAGGGCAAGGTGG

>D100

ATGGACACCTTCAGCACCAAGAGCCTGGCTCTGCAGGCGCAGAAGAAGCTCCTGAGTAAGATGGCGTCCAAGGCAGTGGTGGCCGTGCTGGTGGATGACACCAGCAGTGAGGTGCTGGATGAGCTGTACCGCGCCACCAGGGAGTTCACGCGCAGCCGCAAGGAGGCCCAGAAGATGCTCAAGAACCTGGTCAAGGTGGCCCTGAAGCTGGGACTGCTGCTGCGTGGGGACCAGCTGGGCGGTGAGGAGCTGGCGCTGCTGCGGCGCTTCCGCCACCGGGCGCGCTGCCTGGCCATGACGGCCGTCAGCTTCCACCAGGTGGACTTCACCTTCGACCGGCGCGTGCTGGCCGTCGGGCTGCTCGAGTGCCGCGACCTGCTGCACCAGGCCGTGGGTCCCCACCTGACCG

>D102

ATGGACACCTTCAGCACCAAGAGCCTGGCTCTGCAGGCGCAGAAGAAGCTCCTGAGTAAGATGGCGTCCAAGGCAGTGGTGGCCGTGCTGGTGGATGACACCAGCAGTGAGGTGCTGGATGAGCTGTACCGCGCCACCAGGGAGTTCACGCGCAGCCGCAAGGAGGCCCAGAAGATGCTCAAGAACCTGGTCAAGGTGGCCCTGAAGCTGGGACTGCTGCTG

>D105

ATGGACACCTTCAGCACCAAGAGCCTGGCTCTGCAGGCGCAGAAGAAGCTCCTGAGTAAGATGGCGTCCAAGGCAGTGGTGGCCGTGCTGGTGGATGACACCAGCAGTGAGGTGCTGGATGAG

>D106

ATGGACACCTTCAGCACCAAGAGCCTGGCTCTGCAGGCGCAGAAGAAGCTCCTGAGTAAGATGGCGTCCAAGGCAGTGGTGGCCGTGCTGGTGGATGACACCAGCAGTGAGGTGCTGGATGAGCTGTACCGCGCCACCAGGGAGTTCACGCGCAGCCGCAAGGAGGCCCAGAAGATGCTCAAGAACCTGGTCAAGGTGGCCCTGAAGCTGGGACTGCTGCTGCGTGGGGACCAGCTGGGCGGTGAGGAGCTGGCGCTG

>D107

ATGGACACCTTCAGCACCAAGAGCCTGGCTCTGCAGGCGCAGAAGAAGCTCCTGAGTAAGATGGCGTCCAAGGCAGTGGTGGCCGTGCTGGTGGATGACACCAGCAGTGAGGTGCTGGATGAGCTGTACCGCGCCACCAGGGAGTTCACGCGCAGCCGCAAGGAGGCCCAGAAGATGCTCAAGAACCTGGTCAAGGTGGCCCTGAAGCTGGGACTGCTGCTGCGTGGGGACCAGCTGGGCGGTGAGGAGCTGGCGCTGCTGCGG

>D108

ATGGACACCTTCAGCACCAAGAGCCTGGCTCTGCAGGCGCAGAAGAAGCTCCTGAGTAAGATGGCGTCCAAGGCAGTGGTGGCCGTGCTGGTGGATGACACCAGCAGTGAGGTGCTGGATGAGCTGTACCGCGCCACCAGGGAGTTCACGCGCAGCCGCAAGGAGGCCCAGAAGATGCTCAAGAACCTGGTCAAGGTGG

>D109

ATGGACACCTTCAGCACCAAGAGCCTGGCTCTGCAGGCGCAGAAGAAGCTCCTGAGTAAGATGGCGTCCAAGGCAGTGGTGGCCGTGCTGGTGGATGACACCAGCAGTGAGGTGCTGGATGAGCTGTACCGCGCCACCAGGGAGTTCACGCGCAGCCGCAAGGAGGCCCAGAAGATG

>D110

ATGGACACCTTCAGCACCAAGAGCCTGGCTCTGCAGGCGCAGAAGAAGCTCCTGAGTAAGATGGCGTCCAAGGCAGTGGTGGCCGTGCTGGTGGATGACACCAGCAGTGAGGTGCTGGATGAG

>D112

ATGGACACCTTCAGCACCAAGAGCCTGGCTCTGCAGGCGCAGAAGAAGCTCCTGAGTAAGATGGCGTCCAAGGCAGTGGTGGCCGTGCTGGTGGATGACACCAGCAGTGAGGTGCTGGATGAGCTGTACCGCGCCACCAGGGAGTTCACGCGCAGCCGCAAGGAGGCCCAGAAGATGCTCAAGAACCTGGTCAAGGTGGCCCTGAAGCTGGGACTGCTGCTGCGTGGGGACCAGCTGGGCGGTGAGGAGCTGGCGCTG

>D113

ATGGACACCTTCAGCACCAAGAGCCTGGCTCTGCAGGCGCAGAAGAAGCTCCTGAGTAAGATGGCGTCCAAGGCAGTGGTGGCCGTGCTGGTGGATGACACCAGCAGTGAGGTGCTGGATGAGCTGTACCGCGCCACCAGGGAGTTCACGCGCAGCCGCAAGGAGGCCCAGAAGATGCTCAAGAACCTGGTCAAGGTGGCCCTGAAGCTGGGACTGCTGCTGCGTGGGGACCAGCTGGGCGGTGAGGAGCTGGCGCTGCTGCGGCGCTTCCGCCACCGGGCGCGCTGCCTGGCCATGACGGCCGTCAGCTTCCACCAGGTGGACTTCACCTTCGACCGGCGCGTGCTGGCCGTCGGGCTGCTCGAGTGCCGCGACCTGCTGCACCAGGCCGTGGGTCCCCACCTGACCGCCAAGTCCCACGGCCGCATCAACCACGTGTTCGGCCACCTAGCCGACTGCGACTTCCTGGCTGCGCTCTACGGCCCCGCCGAGCCCTACCG

>D114

ATGGACACCTTCAGCACCAAGAGCCTGGCTCTGCAGGCGCAGAAGAAGCTCCTGAGTAAGATGGCGTCCAAGGCAGTGGTGGCCGTGCTGGTGGATGACACCAGCAGTGAGGTGCTGGATGAGCTGTACCG

>D115

ATGGACACCTTCAGCACCAAGAGCCTGGCTCTGCAGGCGCAGAAGAAGCTCCTGAGTAAGATGGCGTCCAAGGCAGTGGTGGCCGTGCTGGTGGATGACACCAGCAGTGAGGTGCTGGATGAGCTGTACCGCGCCACCAGGGAGTTCACGCGCAGCCGCAAGGAGGCCCAGAAGATGCTCAAGAACCTGGTCAAGGTGGCCCTGAAGCTGGGACTGCTGCTGCGTGGGGACCAGCTGGGCGGTGAGGAGCTGGCGCTG

>D117

ATGGACACCTTCAGCACCAAGAGCCTGGCTCTGCAGGCGCAGAAGAAGCTCCTGAGTAAGATGGCGTCCAAGGCAGTGGTGGCCGTGCTGGTGGATGACACCAGCAGTGAGGTGCTGGATGAGCTGTACCGCGCCACCAGGGAGTTCACGCGCAGCCGCAAGGAGGCCCAGAAGATGCTCAAGAACCTGGTCAAGGTGGCCCTGAAGCTGGGACTGCTGCTGCGTGGGGACCAGCTGGGCGGTGAGGAGCTGGCGCTG

>D118

ATGGACACCTTCAGCACCAAGAGCCTGGCTCTGCAGGCGCAGAAGAAGCTCCTGAGTAAGATGGCGTCCAAGGCAGTGGTGGCCGTGCTGGTGGATGACACCAGCAGTGAGGTGCTGGATGAGCTGTACCGCGCCACCAGGGAGTTCACGCGCAGCCGCAAGGAGGCCCATAAGATGCTCAAGAACCTGGTCTAGGTGGCCCTGAAGCTGGGACTGCTGCTGCGTGGGGACCAGCTGGGCGGTGAGGAGCTGGCGCTG

>D120

ATGGACACCTTCAGCACCAAGAGCCTGGCTCTGCAGGCGCAGAAGAAGCTCCTGAGTAAGATGGCGTCCAAGGCAGTGGTGGCCGTGCTGGTGGATGACACCAGCAGTGAGGTGCTGGATGAGCTGTACCGCGCCACCAGGGAGTTCACGCGCAGCCGCAAGGAGGCCCAGAAGATGCTCGACAACCTGATCAAGGTGG

>D121

ATGGACACCTTCAGCACCAAGAGCCTGGCTCTGCAGGCGCAGAAGAAGCTCCTGAGTAAGATGGCGTCCAAGGCAGTGGTGGCCGTGCTGGTGGATGACACCAGCAGTGAGGTGCTGGATGAG

>D122

ATGGACACCTTCAGCACCAAGAGCCTGGCTCTGCAGGCGCAGAAGAAGCTCCTGAGTAAGATGGCGTCCAAGGCAGTGGTGGCCGTGCTGGTGGATGACACCAGCAGTGAGGTGCTGGATGAGCTGTACCGCGCCACCAGGGAGTTCACGCGCAGCCGCAAGGAGGCCCAGAAGATGCTCAAGAACCTGGTCAAGGTGG

>D123

ATGGACACCTTCAGCACCAAGAGCCTGGCTCTGCAGGCGCAGAAGAAGCTCCTGAGTAAGATGGCGTCCAAGGCAGTGGTGGCCGTGCTGGTGGATGACACCAGCAGTGAGGTGCTGGATGAGCTGTACCGCGCCACCAGGGAGTTCACGCGCAGCCGCAAGGAGGCCCAGAAGATGCTCAAGAACCTGGTCAAGGTGGCCCTGAAGCTGGGACTGCTGCTGCGTGGGGACCAGCTGGGCGGTGAGGAGCTGGCGCTG

>D124

ATGGACACCTTCAGCACCAAGAGCCTGGCTCTGCAGGCGCAGAAGAAGCTCCTGAGTAAGATGGCGTCCAAGGCAGTGGTGGCCGTGCTGGTGGATGACACCAGCAGTGAGGTGCTGGATGAGCTGTACCGCGCCACCAGGGAGTTCACGCGCAGCCGCAAGGAGGCCCAGAAGAT

>D125

ATGGACACCTTCAGCACCAAGAGCCTGGCTCTGCAGGCGCAGAAGAAGCTCCTGAGTAAGATGGCGTCCAAGGCAGTGGTGGCCGTGCTGGTGGATGACACCAGCAGTGAGGTGCTGGATGAGCTGTACCGCGCCACCAGGGAGTTCACGCGCAGCCGCAAGGAGGCCCAGAAGATGCTCAAGAACCTGGTCAAGGTGGCCCTGAAGCTGGGACTGCTGCTGCGTGGGGACCAGCTGGGCGGTGAGGAGCTGGCGCTGCTGCGGCGCTTCCGCCACCGGGCGCGCTGCCTGGCCATGATGGCCGTCAGCTTCCACCAGGTGGACTTCACC

>D126

ATGGACACCTTCAGCACCAAGAGCCTGGCTCTGCAGGCGCAGAAGAAGCTCCTGAGTAAGATGGCGTCCAAGGCAGTGGTGGCCGTGCTGGTGGATGACACCAGCAGTGAGGTGCTGGATGAGCTGTACCGCGCCACCAGGGAGTTCACGCGCAGCCGCAAGGAGGCCCAGAAGATGCTCAAGAACCTGGTCAAGGTGGCCCTGAAGCTGGGACTGCTGCTG

>D127

ATGGACACCTTCAGCACCAAGAGCCTGGNTCTGCAGGCGCAGAAGAAGCTCCTGAGTAAGATGGCGTCCAAGGCAGTGGTGGCCGTGCTGGTGGATGACACCAGCAGTGAGGTGCTGGATGAGCTGTACCGCGCCACCAGGGAGTTC

>D130

ATGGACACCTTCAGCACCAAGAGCCTGGCTCTGCAGGCGCAGAAGAAGCTCCTGAGTAAGATGGCGTCCAAGGCAGTGGTGGCCGTGCTGGTGGATGACACCAGCAGTGAGGTGCTGGATGAGCTGTACCGCGCCACCAGGGAGTTCACGCGCAGCCGCAAGGAGGCCCAGAAGATGCTCAAGAACCTGGTCAAGGTGG

>D133

ATGGACACCTTCAGCACCAAGAGCCTGGCTCTGCAGGCGCAGAAGAAGCTCCTGAGTAAGATGGCGTCCAAGGCAGTGGTGGCCGTGCTGGTGGATGACACCAGCAGTGAGGTGCTGGATGAGCTGTACCGCGCCACCAGGGAGTTCACGCGCAGCCGCAAGGAGGCCCAGAAGATGCTCAAGAACCTGGTCAAGGTGGCCCTGAAGCTGGGACTGCTGCTGCGTGGGGACCAGCTGGGCGGTGAGGAGCTGG

>D134

ATGGACACCTTCAGCACCAAGAGCCTGGCTCTGCAGGCGCAGAAGAAGCTCCTGAGTAAGATGGCGTCCAAGGCAGTGGTGGCCGTGCTGGTGGATGACACCAGCAGTGAGGTGCTGGATGAGCTGTACCGCGCCACCAGGGAGTTCACGCGCAGCAGCAAGGAGGCCCAGAAGATGCTCAAGAACCTGGTCAAGGTGGCCCTGAAGCTGGGACTGCTGCTGCGTGGGGACCAGCTGGGCGGTGAGGAGCTGGCGCTG

>D137

ATGGACACCTTCAGCACCAAGAGCCTGGCTCTGCAGGCGCAGAAGAAGCTCCTGAGTAAGATGGCGTCCAAGGCAGTGGTGGCCGTGCTGGTGGATGACACCAGCAGTGAGGTGCTGGATGAGCTGTACCGCGCCACCAGGGAGTTCACGCGCAGCCGCAAGGAGGCCCAGAAGATGCTCAAGAACCTGGTCAAGGTGGCCCTGAAGCTGGGACTGCTGCTG

>D138

ATGGACACCTTCAGCACCAAGAGCCTGGCTCTGCAGGCGCAGAAAAAGCTCCTGAGTAAAATGGCGTCCAAGGCAGTGGTGGCCGTGCTGGTGTCATCCACCAGCAGTGAGGTGCTGGATGAGCTGTACCGCGCCACCAGGGAGTTCACGCGCAGCCGCAAGGAGGCCCAGAAAATG

>D140

ATGGACACCTTCAGCACCAAGAGCCTGGCTCTGCAGGCGCAGAAGAAGCTCCTGAGTAAGATGGCGTCCAAGGCAGTGGTGGCCGTGCTGGTGGATGACACCAGCAGTGAGGTGCTGGATGAGCTGTACCGCGCCACCAGGGAGTTCACGCGCAGCCGCAAGGAGGCCCAGAAGATGCTCAAGAACCTGGTCAAGGTGGCCCTGAAGCTGGGACTGCTGCTGCGTGGGGACCAGCTGGGCGGTGAGGAGCTGGCGCTG

>D142

ATGGACACCTTCAGCACCAAGAGCCTGGCTCTGCAGGCGCAGAAGAAGCTCCTGAGTAAGATGGCGTCCAAGGCAGTGGTGGCCGTGCTGGTGGATGACACCAGCAGTGAGGTGCTGGATGAGCTGTACCGCGCCACCAGGGAGTTCACG

>D143

ATGGACACCTTCAGCACCAAGAGCCTGGCTCTGCAGGCGCAGAAGAAGCTCCTGAGTAAGATGGCGTCCAAGGCAGTGGTGGCCGTGCTGGTGGATGACACCAGCAGTGAGGTGCTGGATGAGCTGTACCGCGCCACCAGGGAGTTCACGCGCAGCCGCAAGGAGGCCCAGAAGATGCTCAAGAACCTGGTCAAGGTGG

>D144

ATGGACACCTTCAGCACCAAGAGCCTGGCTCTGCAGGCGCAGAAGAAGCTCCTGAGTAAGATGGCGTCCAAGGCAGTGGTGGCCGTGCTGGTGGATGATACCAGCAGTGAGGTGCTGGATGAGCTGTACCGCGCCACCAGGGAGTTCACGCGCAGCCGCAAGGAGGCCCAGAAGATGCTCAAGAACCTGGTCAAGGTGGCCCTGAAGCTGGGACTGCTGCTGCGTGGGGACCAGCTGGGCGGTGAGGAGCTGGCGCTGCTGCGGCGCTTCCGCCACCGGGCGCGCTGCCTGGCCATGACGGCCGTCAGCTTCCACCAGGTGGACTTCACCTTCGACCGGCGCGTGCTGGCCGTCGGGCTGCTCGAGTGCCGCGACCTG

>D145

ATGGACACCTTCAGCACCAAGAGCCTGGCTCTGCAGGCGCAGAAGAAGCTCCTGAGTAAGATGGCGTCCAAGGCAGTGGTGGCCGTGCTGGTGGATGACACCAGCAGTGAGGTGCTGGATGAGCTGTACCGCGCCACCAGGGAGTTCACGCGCAGCCGCAAGGAGGCCCAGAAGATGCTCAAGAACCTGGTCAAGGTGGCCCTGAAGCTGGGACTGCTGCTGCGTGGGGACCAGCTGGGCGGTGAGGAGCTGGCGCTG

>D146

ATGGACACCTTCAGCACCAAGAGCCTGGCTCTGCAGGCGCAGAAGAAGCTCCTGAGTAAGATGGCGTCCAAGGCAGTGGTGGCCGTGCTGGTGGATGACACCAGCAGTGAGGTGCTGGATGAGCTGTACCGCGCCACCAGGGAGTTCACGCGCAGCCGCAAGGAGGCCCAGAAGATGCTCAAGAACCTGGTCAAGGTGGCCCTGAAGCTGGGACTGCTGCTGCGTGGGGACCAGCTGGGCGGTGAGGAGCTGGCGCTGCTGCGGCGCTTCCGCCACCGGGCGCGCTGCCTGGCCATGACGGCCGTCAGCTTCCACC

>D147

ATGGACACCTTCAGCACCAAGAGCCTGGCTCTGCAGGCGCAGAAGAAGCTCCTGAGTAAGATGGCGTCCAAGGCAGTGGTGGCCGTGCTGGTGGATGACACCAGCAGTGAGGTGCTGGATGAGCTGTACCGCGCCACCAGGGAGTTCACGCGCAGCCGCAAGGAGGCCCAGAAGATGCTCAAGAACCTGGTCAAGGTGG

>D148

ATGGACACCTTCAGCACCAAGAGCCTGGCTCTGCAGGCGCAGAAGAAGCTCCTGAGTAAGATGGCGTCCAAGGCAGTGGTGGCCGTGCTGGTGGATGACACCAGCAGTGAGGTGCTGGATGAGCTGTACCGCGCCAC

>D149

ATGGACACCTTCAGCACCAAGAGCCTGGCTCTGCAGGCGCAGAAGAAGCTCCTGAGTAAGATGGCGTCCAAGGCAGTGGTGGCCGTGCTGGTGGATGACACCAGCAGTGAGGTGCTGGATGAGCTGTACCGCGCCACCAGGGAGTTCACGCGCAGCCGCAAGGAGGCCCAGAAGATGCTCAAGAACCTGGTCAAGGTGGCCCTGAAGCTGGGACTGCTGCTGCGTGGGGACCAGCTGGGCGGTGAGGAGCTGGCGCTGCTGCGGCGCTTCCGCCACCGGGCGCGCTGCCTGGCCATGACGGCCGTCAGCTTCCACCAGGTGGACTTCACCTTCGACCGGCGCGTGCTGGCCGTCGGGCTGCTCGAGTGCCGCGACCTGCTGCACCAGGCCGTGGGTCCCCACCTGACCG

>D150

ATGGACACCTTCAGCACCAAGAGCCTGGCTCTGCAGGCGCAGAAGAAGCTCCTGAGTAAGATGGCGTCCAAGGCAGTGGTGGCCGTGCTGGTGGATGACACCAGCAGTGAGGTGCTGGATGAGCTGTACCGCGCCACCAGGGAGTTCACGCGCAGCCGCAAGGAGGCCCAGAAGATGCTGCACCAGGCCGTGGGTCCCCACCTGACCGCCAAGTCCCACGGCCGCATCAACCACGTGTTCGGCCACCTAGCCGACTGCGACTTCCTGGCTGCGCTCTACGGCCCCGCCGAGCCCTACCGCTCCCACCTGCGCAGGATCTGCGAGGGCCTGGGCCGGATGCTGGACGAGGGCAGCCTCACGCGTACGCGGCCGCTCGAGCAGAAACTCATCTCAGAAGC

>D152

ATGGACACCTTCAGCACCAAGAGCCTGGCTCTGCAGGCGCAGAAGAAGCTCCTGAGTAAGATGGCGTCCAAGGCAGTGGTGGCCGTGCTGGTGGATGACACCAGCAGTGAGGTGCTGGATGAGCTGTACCGCGCCACCAGGGAGTTCACGCGCAGCCGCAAGGAGGCCCAGAAGATGCTCAAGAACCTGGTCAAGGTGGCCCTGAAGCTGGGACTGCTGCTGCGTGGGGACCAGCTGGGCGAAGAGGAGCTGGCGCTGCTGCGGCGCTTCCGCCACCGCCTGCNCTGCCTGGTATGGGAGAGCTCCCAATTCGTTGGATGGACTTCACCTTCGANNATAGCGTGCTGGCCGTCGGGCGGCTC

>D153

ATGGACACCTTCAGCACCAAGAGCCTGGCTCTGCAGGCGCAGAAGAAGCTCCTGAGTAAGATGGCGTCCAAGGCAGTGGTGGCCGTGCTGGTGGATGACACCAGCAGTGAGGTGCTGGATGAGCTGTACCGCGCCACCAGGGAGTTCACGCGCAGCCGCAAGGAGGCCCAGAAGATGCTCAAGAACCTGGTCAAGGTGGCCCTGAAGCTGGGACTGCTGCTGCGTGGGGACCAGCTGGGCGGTGAGGAGCTGGCGCTG

>D154

ATGGACACCTTCAGCACCAAGAGCCTGGCTCTGCAGGCGCAGAAGAAGCTCCTGAGTAAGATGGCGTCCAAGGCAGTGGTGGCCGTGCTGGTGGATGACACCAGCAGTGAGGTGCTGGATGAGCTGTACCGCGCCACCAGGGAGTTCACGCGCAGCCGCAAGGAGGCCCAGAAGATGCTCAAGAACCTGGTCAAGGTGGCCCTGAAGCTGGGACTGCTGCTGCGTGGGGACCAGCTGGGCGGTGAGGAGCTGGCGCTGCTGCGGCGCTTCCGCCACCGAGATCGCTG

>D155

ATGGACACCTTCAGCACCAAGAGCCTGGCTCTGCAGGCGCAGAAGAAGCTCCTGAGTAAGATGGCGTCCAAGGCAGTGGTGGCCGTGCTGGTGGATGACACCAGCAGTGAGGTGCTGGATGAGCTGTACCGCGCCACCAGGGAGTTCACGCGCAGCCGCAAGGAGGCCCAGAAGATGCTCAAGAACCTGGTCAAGGTGGCCCTGAAGCTGGGACTGCTGCTGCGTGGGGACCAGCTGGGCGGTGAGGAGCTGGCGCTG

>D156

ATGGACACCTTCAGCACCAAGAGCCTGGCTCTGCAGGCGCAGAAGAAGCTCCTGAGTAAGATGGCGTCCAAGGCAGTGGTGGCCGTGCTGGTGGATGACACCAGCAGTGAGGTGCTGGATGAGCCGTACCG

>D157

ATGGACACCTTCAGCACCAAGAGCCTGGCTCTGCAGGCGCAGAAGAAGCTCCTGAGTAAGATGGCGTCCAAGGCAGTGGTGGCCGTGCTGGTGGATGACACCAGCAGTGAGGTGCTGGATGAGCTGTACCGCGCCACCAGGGAGTTCACGCGCAGCCGCAAGGAGGCCCAGAAGATGCTCAAGAACCTGGTCAAGGTGGCCCTGAAGCTGGGACTGCTGCTGCGTGGGGACCAGCTGGGCGGTGAGGAGCTGGCGCTG

>D158

ATGGACACCTTCAGCACCAAGAGCCTGGCTCTGCAGGCGCAGAAGAAGCTCCTGAGTAAGATGGCGTCCAAGGCAGTGGTGGCCGTGCTGGTGGATGACACCAGCAGTGAGGTGCTGGATGAGCTGTACCGCGCCACCAGGGAGTTCACGCGCAGCCGCAAGGAGGCCCAGAAGATGCTCAAGAACCTGGTCAAGGTGG

>D160

ATGGACACCTTCAGCACCAAGAGCCTGGCTCTGCAGGCGCAGAAGAAGCTCCTGAGTAAGATGGCGTCCAAGGCAGTGGTGGCCGTGCTGGTGGATGACACCAGCAGTGAGGTGCTGGATGAGCTGTACCGCGCCACCAGGGAGTTCACGCGCAGCCGCAAGGAGGCCCAGAAGATGCTCAAGAACCTGGTCAAGGTGGCCCTGAAGCTGGGACTGCTGCTGCGTGGGGACCAGCTGGGCGGTGAGGAGCTGGCGCTG

>D161

ATGGACACCTTCAGCACCAAGAGCCTGGCTCTGCAGGCGCAGAAGAAGCTCCTGAGTAAGATGGCGTCCAAGGCAGTGGTGGCCGTGCTGGTGGATGACACCAGCAGTGAGGTGCTGGATGAGCTGTACCGCGCCACCAGGGAGTTCCCACCCAACCACAGGGAGGCACATAATATG

>D162

ATGGACACCTTCAGCACCAAGAGCCTGGCTCTGCAGGCGCAGAAGAAGCTCCTGAGTAAGATGGCGTCCGAGGCANTGGTGGNCGTGCTGGTGGATGACACCAGCAGTGAGGTGCTGGNTGANCTGTTCCGCGCCACCAGGGAGTTCACNCGCCTCCGGTANGAGGCCCAGAANATGCTCAAGAACCTGGTCAAGGTGG

>D163

ATGGACACCTTCAGCACCAAGAGCCTGGCTCTGCAGGCGCAGAAGAAGCTCCTGAGTAAGATGGCGTCCAAGGCAGTGGTGGCCGTGCTGGTGGATGACACCAGCAGTGAGGTGCTGGATGAGCTGTACCGCGCCACCAGGGAGTTCACGCGCAGCCGCAAGGAGGCCCAGAAGATGCTCAAGAACCTGGTCAAGGTGGCCCTGAAGCTGGGACTGCTGCTGCGTGGGGACCAGCTGGGCGGTGAGGAGCTGGCGCTGCTGCGGCGCTTCCGCCACCGGGCGCGCTGCCTGGCCATGACGGCCGTCAGCTTCCACCAGGTGGACTTCACCTTCGACCGGCGCGTGCTGGCCGTCGGGCTGCTCGAGTGCCGCGACCTGCTGCACCAGGCCGTGGGTCCCCACCTGACCGCCAAGTCCCACGGCCGCATCAACCACGTGTTCGGCCACCTAGCCGACTGCGACTTCCTGGCTGCGCTCTACGGCCCCGCCGAGCCCTACCGCTCCCACCTGCGCAGGATCTGCGAGGGCCTGGGCCGGATGCTGGACGAGGGCAGCCTCACGCGT

>D164

ATGGACACCTTCAGCACCAAGAGCCTGGCTCTGCAGGCGCAGAAGAAGCTCCTGAGTAAGATGGCGTCCAAGGCAGTGGTGGCCGTGCTGGTGGATGACACCAGCAGTGAGGTGCTGGATGAGCTGTACCGTGCCACCAGGGAGTTCACGCGCAGCCGCAAGGATGTCCAGAAGATGCTCAAGAACCTGGTCAAGGTGGCCCTGAAGCTGGGACTGCTG

>D165

ATGGACACCTTCAGCACCAAGAGCCTGGCTCTGCAGGCGCAGAAGAAGCTCCTGAGTAAGATGGCGTCCAAGGCAGTGGTGGCCGTGCTGGTGGATGACACCAGCAGTGAGGTGCTGGATGAGCTGTACCGCGCCACCAGGGAGTTCACGCGCAGCCGCAAGGAGGCCCAGAAGATGCTCAAGAACCTGGTCAAGGTGGCCCTGAAGCTGGGACTGCTGCTGCGTGGGGACCAGCTGGGCGGTGAGGAGCTGGCGCTGCTGCGGCGCTTCCGCCACCGGGCGCGCTGCCTGGCCATGACGGCCGTCAGCTTCCACCAGGTGGACTTCACCTTCGACCGGCGCGTGCTGGCCGTCGGGCTGCTCGAGTGCCGCGACCTGCTGC

>D166

ATGGACACCTTCAGCACCAAGAGCCTGGCTCTGCAGGCGCAGAAGAAGCTCCTGAGTAAGATGGCGTCCAAAGCAGAGGAGGCGTGCTGATGGATGACAAATTCGCGGAGGTGCTGGATGANCCATACCNCGCCACCTCGGAGTTCACTCGATGCCGCNTGAGGTATTCTNNATGGTCACCAACCTGNCTTGGGTGGCCCTGGGTCTGGGACGGCTGCTGCGTGAGGACGATCTGGGCTGTGAGGAGCTGGCGCTG

>D168

ATGGACACCTTCAGCACCAAGAGCCTGGCTCTGCAGGCGCAGAAGAAGCTCCTGAGTAAGATGGCGTCCAAGGCAGTGGTGGCCGTGCTGGTGGATGACACCAGCAGTGAGGTGCTGGATGAGCTGTACCGCGCCACCAGGGAGTTCACGCGCAGCCGCAAGGAGGCCCAGAAGATGCTCAAGAACCTGGTCAAGGTGGCCCTGAAGCTGGGACTGCTGCTGCGTGGGGACCAGCTGGGCGGTGAGGAGCTGGCGCTGCTGCGGCGCTTCCGCCACCGGGCGCGCTGCCTGGCCATGACGGCCGTCAGCTTCCACCAGGTGGACTTCACCTTCGACCGGCGCGTGCTGGCCGTCGGGCTGCTCGAGTGCCGCGACCTGCTGCACCAGGCCGTGGGTCCCCACCTGACCG

>D169

ATGGACACCTTCAGCACCAAGAGCCTGGCTCTGCAGGCGCAGAAGAAGCTCCTGAGTAAGATGGCGTCCAAGGCAGTGGTGGCCGTGCTGGTGGATGACACCAGCAGTGAGGTGCTGGATGAGCTGTACCGCGCCACCAGGGAGTTCACGCGCAGCCGCAAGGAGGCCCAGAAGATGCTCAAGAACCTGGTCAAGGTGGCCCTGAAGCTGGGACTGCTGCTGCGTGGGGACCAGCTGGGCGGTGAGGAGCTGGCGCTG

>D170

ATGGACACCTTCAGCACCAAGAGCCTGGCTCTGCAGGCGCAGAAGAAGCTCCTGAGTAAGATGGCGTCCAAGGCAGTGGTGGCCGTGCTGGTGGATGACACCAGCAGTGAGGTGCTGGATGAGCTGTACCGCGCCACCAGGGAGTTC

>D171

ATGGACACCTTCAGCACCAAGAGCCTGNCTCTGCAGGCGCAGAAGAAGCTCCTGAGTAAGATGGCGTCCAAGGCAGTGGTGGCCGTGCTGGTGGATGACACCAGCAGTGAGGTGCTGGATGAGCTGTACCGCGCCACCAGGGAGTTCACGCGCAGCCGCAAGGAGGCCCAGAAGATGCTCAAGAACCTGGTCAAGGTGGCCCTGAAGCTGGGACTGCTGCTGCGTGGGGACCAGCTGGGCGGTGAGGAGCTGGCGCTGCTGCGGCGCTTCCGCCACCGGGCGCGCTG

>D172

ATGGACACCTTCAGCACCAAGAGCCTGGCTCTGCAGGCGCAGAAGAAGCTCCTGAGTAAGATGGCGTCCAAGGCAGTGGTGGCCGTGCTGGTGGATGACACCAGCAGTGAGGTGCTGGATGAGCTGTACCGCGCCACCAGGGAGTTCACGCGCAGCCGCAAGGAGGCCCAGAAGATGCTCAAGAACCTGGTCAAGGTGGCCCTGAAGCTGGGACTGCTGCTGCGTGGGGACCAGCTGGGCGGTGAGGAGCTGGCGCTG

>D173

ATGGACACCTTCAGCACCAAGAGCCTGGCTCTGCAGGCGCAGAAGAAGCTCCTGAGTAAGATGGCGTCCAAGGCAGTGGTGGCCGTGCTGGTGGATGACACCAGCAGTGAGGTGCTGGATGAGCTGTACCGCGCCACCAGGGAGTTCACGCGCAGCCGCAAGGAGGCCCAGAAGATG

>D174

ATGGACACCTTCAGCACCAAGAGCCTGGCTCTGCAGGCGCAGAAGAAGCTCCTGAGTAAGATGGCGTCCAAGGCAGTGGTGGCCGTGCTGGTGGATGACACCAGCAGTGAGGTGCTGGATGAGCTGTACCGCGCCACCAGGGAGTTCACGCGCAGCCGCAAGGAGGCCCAAAAGATGCTCAAGAACCTGGTCAAGGTGGCCCTGAAACTGGGACAGCTGCTGCGTGGCGACCGACTGGGCGGTGANGANAGGCGCTGCTGCCGCGCTTCCGCCACCGGGCGCGCTGCATGGCCATGACGGCCGTCCTCTTCCACCAGGTGGACTTCACCTTCGA

>D175

ATGGACACCTTCAGCACCAAGAGCCTGGCTCTGCAGGCGCAGAAGAAGCTCCTGAGTAAGATGGCGTCCAAGGCAGTGGTGGCCGTGCTGGTGGATGACACCAGCAGTGAGGTCCTGGATGAGCTGTACCGCGCCACCAGGGAGTTCACGCGCAGCCGCAAGGAGGCCCAGAAGATGCTCAAGAACCTGGTCAAGGTGG

>D176

ATGGACACCTTCAGCACCAAGAGCCTGGCTCTGCAGGCGCAGAAGAAGCTCCTGAGTAAGATGGCGTCCAAGGCAGTGGTGGCCGTGCTGGTGGATGACACCAGCAGTGAGGTGCTGGATGAGCTGTACCGCGCCACCAGGGAGTTCACGCGCAGCCGCAAGGAGGCCCAGAAGATGCTCAAGAACCTGGTCAAGGTGGCCCTGAAGCTGGGACTGCTGCTGCGTGGGGACCAGCTGGGCGGTGAGGAGCTGGCGCTG

>D177

ATGGACACCTTCAGCACCAAGAGCCTGGCTCTGCAGGCGCAGAAGAAGCTCCTGAGTAAGATGGCGTCCAAGGCAGTGGTGGCCGTGCTGGTGGATGACACCAGCAGTGAGGTGCTGGATGAGCTGTACCGCGCCACCAGGGAGTTCACGCGCAGCCGCAAGGAGGCCCAGAAGATGCTCAAGAACCTGGTCAAGGTGGCCCTGAAGCTGGGACTGCTGCTGCGTGGGGACCAGCTGGGCGGTGAGGAGCTGGCGCTG

>D178

ATGGACACCTTCAGCACCAAGAGCCTGGCTCTGCAGGCGCAGAAGAAGCTCCTGAGTAAGATGGCGTCCAAGGCAGTGGTGGCCGTGCTGGTGGATGACACCAGCAGTGAGGTGCTGGATGAGCTGTACCGCGCCACCAGGGAGTTCACGCGCAGCCGCAAGGAGGCCCAGAAGATGCTCAAGAACCTGGTCAAGGTGGCCCTGAAGCTGGGACTGCTGCTG

>D179

ATGGACACCTTCAGCACCAAGAGCCTGGCTCTGCAGGCGCAGAAGAAGCTCCTGAGTAAGATGGCGTCCAAGGCAGTGGTGGCCGTGCTGGTGGATGACACCAGCAGTGAGGTGCTGGATGAGCTGT

>D180

ATGGACACCTTCAGCACCAAGAGCCTGGCTCTGCAGGCGCAGAAGAAGCTCCTGAGTAAGATGGCGTCCAAGGCAGTGGTGGCCGTGCTGGTGGATGACACCAGCAGTGAGGTGCTGGATGAGCTGTACCGCGCCACCAGGGAGTTCACGCGCAGCCGCAAGGAGGCCCAGAAGATGCTCAAGAACCTGGTCAAGGTGGCCCTGAAGCTGGGACTGCTGCTGCGTGGGGACCAGCTGGGCGGTGAGGAGCTGGCGCTG

>D181

ATGGACACCTTCAGCACCAAGAGCCTGGCTCTGCAGGCGCAGAAGAAGCTCCTGAGTAAGATGGCGTCCAAGGCAGTGGTGGCCGTGCTGGTGGATGACACCAGCAGTGAGGTGCTGGATGAGCTGTACCGCGCCACCAGGGAGTTCACGCGCAGCCGCAAGGAGGCCCAGAAGATGCTCAAGAACCTGGTCAAGGTGG

>D183

ATGGACACCTTCAGCACCAAGAGCCTGGCTCTGCAGGCGCAGAAGAAGCTCCTGAGTAAGATGGCGTCCAAGGCAGTGGTGGCCGTGCTGGTGGATGACACCAGCAGTGAGGTGCTGGATGAGCTGTACCGCGCCACCAGGGAGTTCACGCGCAGCCGCAAGGAGGCCCAGAAGATGCTCACGAACCGGCGGCATGCGACCTNGAAACTGGGACTGCTGCTG

>D184

ATGGACACCTTCAGCACCAAGAGCCTGGCTCTGCAGGCGCAGAAGAAGCTCCTGAGTAAGATGGCGTCCAAGGCAGTGGTGGCCGTGCTGGTGGATGACACCAGCAGTGAGGTGCTGGATGAGCTGTACCGCGCCACCAGGGAGTTCACGCGCAGCCGCAAGGAGGCCCAGAAGATG

>D186

ATGGACACCTTCAGCACCAAGAGCCTGGCTCTGCAGGCGCAGAAGAAGCTCCTGAGTAAGATGGCGTCCAAGGCAGTGGTGGCCGTGCTGGTGGATGACACCAGCAGTGAGGTGCTGGATGAGCTGTACCGCGCCACCAGGGAGTTCACGCGCAGCCGCAAGGAGGCCCAGAAGATGCTCAAGAACCTGGTCAAGGTGGCCCTGAAGCTGGGACTGCTGCTGCGTGGGGACCAGCTGGGCGGTGAGGAGCTGGCGCTGCTGCGGCGCTTCCGCCACCGGGCGCGCTGCCTGGCCATGACGGCCGTCAGCTTCCACCAGGTGGACTTCACCTTCGACCGGCGCGTGCTGGCCGTCGGGCTGCTCGAGTGCCGCGACCTGCT

>D187

ATGGACACCTTCAGCACCAAGAGCCTGGCTCTGCAGGCGCAGAAGAAGCTCCTGAGTAAGATGGCGTCCAAGGCAGTGGTGGCCGTGCTGGTGGATGACACCAGCAGTGAGGTGCTGGATGAGCTGTACCGCGCCACCAGGGAGTTCACGCGCAGCCGCAAGGAGGCCCAGAAGATGCTCAAGAACCTGGTCAAGGTGGCCCTGAAGCTGGGACTGCTGCTGCGTGGGGACCAGCTGGGCGGTGAGGAGCTGGCGCTGCTGCGGCGCTTCCGCCACCGGGCGCGCTGCCTGGCCATGACGGCCGTCAGCTTCCACCAGGTGGACTTCACCTTCGACCGGCGCGTG

>D188

ATGGACACCTTCAGCACCAAGAGCCTGGCTCTGCAGGCGCAGAAGAAGCTCCTGAGTAAGATGGCGTCCAAGGCAGTGGTGGCCGTGCTGGTGGATGACACCAGCAGTGAGGTGCTGGATGAGCTGTACCGCGCCACCAGGGAGTTCACGCGCAGCCGCAAGGAGGCCCAGAAGATGCTCAAGAACCTGGTCAAGGTGGCCCTGAAGCTGGGACTGCTGCTGCGTGGGGACCAGCTGGGCGGTGAGGAGCTGGCGCTGCTGCGGCGCTTCCGCCACCGGGCGCGCTGCCTGGCCATGACGGCCGTCAGCTTCCACCAGGTGGACTTCACCTTCGACCGGCGCGTGCTGGCCGTCGGGCTGCTCGAGTGCCGCGACCTGCTGCACCAGGCCGTGGGTCCCCACCTGACCGCCAAGTCCCACGGCCGCATCAACCACGTGTTCGGCCACCTAGCCGACTGCGACTTCCTGGCTGCGCTCTACGGCCCCG

>D189

ATGGACACCTTCAGCACCAAGAGCCTGGCTCTGCAGGCGCAGAAGAAGCTCCTGAGTAAGATGGCGTCCAAGGCAGTGGTGGCCGTGCTGGTGGATGACACCAGCAGTGAGGTGCTGGATGAGCTGTACCGCGCCACCAGGGAGTTCACGCGCAGCCGCAAGGAGGCCCAGAAGATGCTCAAGAACCTGGTCAAGGTGGCCCTGAAGCTGGGACTGCTGCTGCGTGGGGACCAGCTGGGCGGTGAGGAGCTGGCGCTGCTGCGGCGCTTCCGCCACCGGGCGCGCTGCCTGGCCATGACGGCCGTCAGCTTCCACCAGGTGGACTTCACCTTCGACCGGCGCGTGCTGGCCGTCGGGCTGCTCGAGTGCCGCGACCTGCTGCACCAGGCCGTGGGTCCCCACCTGACCGCCAAGTCCCACGGCCGCATCAACCACGTGTTCGGCCACCTAGCCGACTGCGACTTCCTGGCTGCG

>D190

ATGGACACCTTCAGCACCAAGAGCCTGGCTCTGCAGGCGCAGAAGAAGCTCCTGAGTAAGATGGCGTCCAAGGCAGTGGTGGCCGTGCTGGTGGATGACACCAGCAGTGAGGTGCTGGATGAGCTGTACCGCGCCACCAGGGAGTTCACGCGCAGCGGCATACGAGATCGTGATGAATTCGCGGCCGCCTGCAGGTCGACCATATGGGAGAGCTCCCAACGCGTTGGATGCATAGCTTGAGTATTCTATAGTGTCACCTAAATAGCTTGGCGTAATCATGGTCATAGCTGTTTCCTGTGTGAAATTGTTATCCGCTCACAATTCCACACAACATACGAGCCGGAAGCATAAAGTGTAAAGCCTGGGGTGCCTAATGAGTGAGCTAACTCACATTAATTGC

>D191

ATGGACACCTTCAGCACCAAGAGCCTGGCTCTGCAGGCGCAGAAGAAGCTCCTGAGTAAGATGGCGTCCAAGGCAGTGGTGGCCGTGCTGGTGGATGACACCAGCAGTGAGGTGCTGGATGAGCTGTACCGCGCCACTAGGGAGTTCACGCGCAGCCGCAAGGAGGCCCAGAAGATGCTCAAGAACCTGGTCAAGGTGGCCCTGAAGCTGGGACTGCTGCTGCGTGGGGACCAGCTGGGCGGTGAGGAGCTGGCGCTGCTGCGGCGCTTCCGCCACCGGGCGCGCTGCCTGGCCATGACGGCCGTCAGCTTCCACCAGGTGGACTTCACCTTCGACCGGCGCGTGCTGGCCGTCGGGCTGCTCGAGTGCCGCGACCTGCTGCACCAGGCCGTGGGTCCCCACCTGACCGCCAAGTCCCACGGCCGCATCAACCACGTGTTCGGCCACCTAGCCGACTGCGACTTCCTGGCTGCGCTCTACGGCCCCGCCGAGCCCTACCGCTCCCACCTGCGCAGGATCTG

**DGAT2 Fragment sequences**

>Primer sequence

ATGAAGACCCTCATAGCCG

>D2

ATGAAGACCCTCATAGCCGCCTACTCCGGGGTCCTGCGCGGCGAGCGTCAGGCCGAGGCTGACCGGAGCCAGCGCTCTCACGGAGGACCTGCGCTGTCGCGCGAGGGGTCTGGGAGATGGGGCACTGGATCCAGCATCCTCTCCGCCCTCCAGGACCTCTTCTCTGTCACCTGGCTCAATAGGTCCAAGGTGGAAAAGCAGCTACAGGTCATCTCAGTGCTCCAGTGGGTCCTGTCCTTCCTTGTACTGGGAGTGGCCTGCAGTGCCATCCTCATGTACATATTCTGCACTGATTGCTGGCTCATCGCTGTG

>D3

ATGAAGACCCTCATAGCCGCCTACTCCGGGGTCCTGCGCGGCGAGCGTCAGGCCGAGGCTGACCGGAGCCAGCGCTCTCACGGAGGACCTGCGCTGTCGCGCGAGGGGTCTGGGAGATGGGGCACTGGATCCAGCATCCTCTCCGCCCTCCAGGACCTCTTCTCTGTCACCTGGCTCAATAGGTCCAAGGTGGAAAAGCAGCTACAGGTCATCTCAGTGCTCCAGTGGGTCCTGTCCTTCCTTGTACTGGGAGTGGCCTGCAGTGCCATCCTCATGTACATATTCTGCACTGATTGCTGGCTCATCGCTGTGCTCTACTTCACTTGGCTGGTGTTTGACTGGAACACACCCAAGAAAGGTGGCAGGAGGTCACAGTGGGTCCGAAACTGGGCTGTGTGGCGCTACTTTCGAGACTACTTTCCCATCCAGCTGGTGAAGACACACAACCTGCTGACCACCAGGAACTATATC

>D5

ATGAAGACCCTCATAGCCGCCTACTCCGGGGTCCTGCGCGGCGAGCGTCAGGCCGAGGCTGACCGGAGCCAGCGCTCTCACGGAGGACCTGCGCTGTCGCGCGAGGGGTCTGGGAGATGGGGCACTGGATCCAGCATCCTCTCCGCCCTCCAGGACCTTTCTCTGTCACCTGGCTCAATAGGTCCAAGGTGGAAAAGCAGCTACAGGTCATCTCAGTGCTCCAGTGGGTCCTGTCCTTCCTTGTACTGGGAGTGGCCTGCAGTGCCATCCTCATGTACATATTCTGCACTGATTGCTGGCTCATCGCTGTG

>D6

ATGAAGACCCTCATAGCCGCCTACTCCGGGGTCCTGCGCGGCGAGCGTCAGGCCGAGGCTGACCGGAGCCAGCGCTCTCACGGAGGACCTGCGCTGTCGCGCGAGGGGTCTGGGAGATGGGGCACTGGATCCAGCATCCTCTCCGCCCTCCAGGACCTCTTCTCTGTCACCTGGCTCAATAGGTCCAAGGTGGAAAAG

>D7

ATGAAGACCCTCATAGCCGCCTACTCCGGGGTCCTGCGCGGCGAGCGTCAGGCCGAGGCTGACCGG

>D8

ATGAAGACCCTCATAGCCGCCTACTCCGGGGTCCTGCGCGGCGAGCGTCAGGCCGAGGCTGACCGGAGCCAGCGCTCTCACGGAGGACCTGCGCTGTCGCGCGAGGGGTCTGGGAGATGGGGCACTGGATCCAGCATCCTCTCCGCCCTCCAGGACCTCTTCTCTGTCACCTGGCTCAATAGGTCCAAGGTGGAAAAGCAGCTACAGGTCATCTCAGTGCTCCAGTGGGTCCTGTCCTTCCTTGTACTGGGAGTGGCCTGCAGTGCCATCCTCATGTACATATTCTGCACTGATTGCTGGCTCATCGCNNTGCTCTACTTCACTTGGCTGGTGTTTGACTGGAACACACCCAAGAAAGGTGGCAGGAGGTCACAGTGGGTCCGAAACTGGGCTGTGTGGCGCTACTTTCGAGACTACTTTCCCATCCAGCTGGTGAAGACACACAACCTGCTGACCACCAGGAACTATATCTTTGGATACCACCCCCATGGTATCATGGGCCTGGGTGCCTTCTGCAACTTCAGCACAGAGGCCACAGAAGTGAGCAAGAAGTTCCCAGGCATACGGCCTTACCTGGCTACACTGGCAGGCAACTTCCGAATGCCTGTGTTGAGGGAGTACCTGATGTCTGGAGGTATCTGCCCTGTCAGCCGGGACACCATAGACTATTTG

>D11

ATGAAGACCCTCATAGCCGCCTACTCCGGGGTCCTGCGCGGCGAGCGTCAGGCCGAGGCTGACCGGAGCCAGCGCTCTCACGGAGGACCTGCGCTGTCGCGCGAGGGGTCTGGGAGATGGGGCACTGGATCCAGCATCCTCTCCGCCCTCCAGGACCTCTTCTCTGTCACCTGGCTCAATAGGTCCAAGGTGGAAAAGCAGCTACAGGTCATCTCAGTGCTCCAGTGGGTCCTGTCCTTCCTTGTACTGGGAGTGGTTGCAGTGCCATCCTCATGTACATATTCTG

>D12

ATGAAGACCCTCATAGCCGCCTACTCCGGGGTCCTGCGCGGCGAGCGTCAGGCCGAGGCTGACCGGAGCCAGCGCTCTCACGGAGGACCTGCGCTGTCGCGCGAGGGGTCTGGGAGATGGGGCACTGGATCCAGCATCCTCTCCGCCCTCCAGGACCTCTTCTCTGTCACCTGGCTCAATAGGTCCAAGGTGGAAAAGCAGCTACAGGTCATCTCAGTGCTCCAGTGGGTCCTGTCCTTCCTTGTACTGGGAGTGGCCTGCAGTGCCATCCTCATGTACATATTCTGCACTGATTGCTGGCTCATCGCTGTGCTCTACTTCACTTGGCTGGTGTTTGACTGGAACACACCCAAGAAAGGTGG

>D16

ATGAAGACCCTCATAGCCGCCTACTCCGGGGTCCTGCGCGGCGAGCGTCAGGCCGAGGCTGACCGGAGCCAGCGCTCTCACGGAGGACCTGCGCTGTCGCGCGAGGGGTCTGGGAGATGGGGCACTGGATCCAGCATCCTCTCCGCCCTCCAGGACCTCTTCTCTGTCACCTGGCTCAATAGGTCCAAGGTGGAAAAGCAGCTACAGGTCATCTCAGTGCTCCAGTGGGTCCTGTCCTTCCTTGTACTGGGAGTGGCCTGCAGTGCCATCCTCATGTACATATTCTGCACTGATTGCTGGCTCATCGCTGTGCTCTACTTCACTTGGCTGGTGTTTGACTGGAACACACCCAAGAAAGGTGGCAGGAGGTCACAGTGGGTCCGAAACTGGGCTGTGTGGCGCTACTTTCGAGACTACTTTCCCATCCAGCTGGTGAAGACACACAACCTGCTGACCACCAGGAACT

>D17

ATGAAGACCCTCATAGCCGCCTACTCCGGGGTCCTGCGCGGCGAGCGTCAGGCCGAGGCTGACCGGAGCCAGCGCTCTCACGGAGGACCTGCGCTGTCGCGCGAGGGGTCTGGGAGATGGGGCACTGGATCCAGCATCCTCTCCGCCCTCCAGGACCTCTTCTCTGTCACCTGGCTCAATAGGTCCAAGGTGGAAAAGCAGCTACAGGTCATCTCAGTGCTCCAGTGGGTCCTGTCCTTCCTTGTACTGGGAGTGGCCTGCAGTGCCATCCTCATGTACATATTCTGCACTGATTGCTGGCTCATCAC

>D18

ATGAAGACCCTCATAGCCGCCTACTCCGGGGTCCTGCGCGGCGAGCGTCAGGCCGAGGCTGACCGGAGCCAGCGCTCTCACGGAGGACCTGCGCTGTCGCGCGAGGGGTCTGGGAGATGGGGCACTGGATCCAGCATCCTCTCCGCCCTCCAGGACCTCTTCTCTGTCACCTGGCTCAATAGGTCCAAGGTGGAAAAGCAGCTACAGGTCATCTCAGTGCTCCAGTGGGTCCTGTCCTTCCTTGTACTGGGAGTGGCCTGCAGTGCCATCCTCATGTACATATTCTGCACTGATTGCTGGCTCATCGCTGTGCTCTACTTCACTTGGCTGGTGTTTGACTGGAACACACCCAAGAAAGGTGGCAGGAGGTCACAGTGGGTCCGAAACTGGGCTGTGTGGCGCTACTTTCGAGACTACTTTCCCATCCAGCTGGTGAAGACACACAACCTGCTGACCACCAGGAACTATATCTTTGGATACCACCCCCATGGTATCATGGGCCTGGGTGCCTTCTGCAACTTCAG

>D19

ATGAAGACCCTCATAGCCGCCTACTCCGGGGTCCTGCGCGGCGAGCGTCAGGCCGAGGCTGACCGGAGCCAGCGCTCTCACGGAGGACCTGCGCTGTCGCGCGAGGGGTCTGGGAGATGGGGCACTGGATCCAGCATCCTCTCCGCCCTCCAGGACCTCTTCTCTGTCACCTGGCTCAATAGGTCCAAGGTGGAAAAGCAGCTACAGGTCATCTCAGTGCTCCAGTGGGTCCTGTCCTTCCTTGTACTGGGAGTGGCCTGCAGTGCCATCCTCATGTACATATTCTGCACTGATTGCTGGCTCATCG

>D20

ATGAAGACCCTCATAGCCGCCTACTCCGGGGTCCTGCGCGGCGAGCGTCAGGCCGAGGCTGACCGGAGCCAGCGCTCTCACGGAGGACCTGCGCTGTCGCGGAGGGGTCTGGGAGATGGGGCACTGGATCCAGCATCCTCTCCGCCCTCCAGGACCTCTTCTCTGTCACCTGGCTCAATAGGTCCAAGGTGGAAAAGCAGCTACAGGTCATCTCAGTGCTCCAGTGGGTCCTGTCCTTCCTTGTACTGGGAGTGGCCTG

>D21

ATGAAGACCCTCATAGCCGCCTACTCCGGGGTCCTGCGCGGCGAGCGTCAGGCCGAGGCTGACCGGAGCCAGCGCTCTCACGGAGGACCTGCGCTGTCGCGCGAGGGGTCTGGGAGATGGGGCACTGGATCCAGCATCCTCTCCGCCCTCCAGGACCTCTTCTCTGTCACCTGGCTCAATAGGTCCAAGGTGGAAAAGCAGCTACAGGTCATCTCAGTGCTCCAGTGGGTCCTGTCCTTCCTTGTACTGGGAGTGGCCTGCAGTGCCATCCTCATGTACATATTCTGCACTGATTGCTGGCTCATCGCTGTGCTCTACTTCACTTGGCTGGTGTTTGA

>D23

ATGAAGACCCTCATAGCCGCCTACTCCGGGGTCCTGCGCGGCGAGCGTCAGGCCGAGGCTGACCGGAGCCAGCGCTCTCACGGAGGACCTGCGCTGTCGCGCGAGGGGTCTGGGAGATGGGGCACTGGATCCAGCATCCTCTCCGCCCTCCAGGACCTCTTCTCTGTCACCTGGCTCAATAGGTCCAAGGTGGAAAAGCAGCTACAGGTCATCTCAGTGCTCCAGTGGGTCCTGTCCTTCCTTGTACTGGGAGTGGCCTGCAGTGCCATCCTCATGTACATATTCTGCACTGATTGCTGGCTCATCGCTGTGCTCTACTTCACTTGGCTGGTGTTTGACTGGAACACACCCAAGAAAGGTGGCAGGAGGTCACAGTGGGTCCGAAACTGGGCTGTGTGGCGCTACTTTCGAGACTACTTTCCCATCCAGCTGGTGAAGACACACAACCTGCTGACCACCAGGAACTATATCTTTGGATACCACCCCCATGGTATCATGGGCCTGGGTGCCTTCTGCAACTTCAGCACAGAGGCCACAGAAGTGAGCAAGAAGTTCCCAGGCATACGG

>D25

ATGAAGACCCTCATAGCCGCCTACTCCGGGGTCCTGCGCGGCGAGCGTCAGGCCGAGGCTGACCGGAGCCAGCGCTCTCACGGAGGACCTGCGCTGTCGCGCGAGGGGTCTGGGAGATGGGGCACTGGATCCAGCATCCTCTCCGCCCTCCAGGACCTCTTCTCTGTCACCTGGCTCAATAGGTCCAAGGTGGAAAAGCAGCTACAGGTCATCTCAGTGCTCCAGTGGGTCCTGTCCTTCCTTGTACTGGGAGTGGCCTGCAGTGCCATCCTCATGTACATATTCTGCACTGATTGCTGGCTCATCGCTGTGCTCT

>D26

ATGAAGACCCTCATAGCCGCCTACTCCGGGGTCCTGCGCGGCGAGCGTCAGGCCGAGGCTGACCGGAGCCAGCGCTCTCACGGAGGACCTGCGCTGTCGCGCGAGGGGTCTGGGAGATGGGGCACTGGATCCAGCATCCTCTCCGCCCTCCAGGACCTCTTCTCTGTCACCTGGCTCAATAGGTCCAAGGTGGAAAAGCAGCTACAGGTCATCTCAGTGCTCCAGTGGGTCCTGTCCTTCCTTGTACTGGGAGTGGCCTGCAGTGCCATCCTCATGTACATATTCTGCACTGATTGCTGGCTCATCGCTGTGCTCTACTTCACTTGGCTGGTGTTTGACTGGAACACACCCAAGAA

>D27

ATGAAGACCCTCATAGCCGCCTACTCCGGGGTCCTGCGCGGCGAGCGTCAGGCCGAGGCTGACCGGAGCCAGCGCTCTCACGGAGGACCTGCGCTGTCGCGCGAGGGGTCTGGGAGATGGGGCACTGGATCCAGCATCCTCTCCGCCCTCCAGGACCTCTTCTCTGTCACCTGGCTCAATAGGTCCAAGGTGGAAAAGCAGCTACAGGTCATCTCAGTGCTCCAGTGGGTCCTGTCCTTCCTTGTACTGGGAGTGGCCTGCAGTGCCATCCTCATGTACATATTCTGCACTGATTGCTGGCTCATCGCTGTGCTCTACTTCACTTGGCTGGTGTTTGACTGGAACACACCCAAGAAAGGTGGCAGGAGGTCACAGTGGGTCCGAAACTGGGCTGTGTGGCGCTACTTTCGAGACTACTTTCCCATCCAGCTGGTGAAGACACACAACCTGCTGACCACCAGGAACTAT

>D28

ATGAAGACCCTCATAGCCGCCTACTCCGGGGTCCTGCGCGGCGAGCGTCAGGCCGAGGCTGACCGGAGCCAGCGCTCTCACGGAGGACCTGCGCTGTCGCGCGAGGGGTCTGGGAGATGGGGCACTGGATCCAGCATCCTCTCCGCCCTCCAGGACCTCTTCTCTGTCACCTGGCTCAATAGGTCCAAGGTGGAAAAGCAGCTACAGGTCATCTCAGTGCTCCAGTGGGTCCTGTCCTTCCTTGTACTGGGAGTGGCCTGCAGTGCCATCCTCATGTACATATTCTGCACTGATTGCTGGCTCATCG

>D30

ATGAAGACCCTCATAGCCGCCTACTCCGGGGTCCTGCGCGGCGAGCGTCAGGCCGAGGCTGACCGGAGCCAGCGCTCTCACGGAGGACCTGCGCTGTCGCGCGAGGGGTCTGGGAGATGGGGCACTGGATCCAGCATCCTCTCCGCCCTCCAGGACCTCTTCTCTGTCACCTGGCTCAATAGGTCCAAGGTGGAAAAG

>D31

CCTACTCCGGGGTCCTGCGCGGCGAGCGTCAGGCCGAGGCTGACCGGAGCCAGCGCTCTCACGGAGGACCTGCGCTGTCGCGCGAGGGGTCTGGGAGATGGGGCACTGGATCCAGCATCCTCTCCGCCCTCCAGGACCTCTTCTCTGTCACCTGGCTCAATAGGTCCAAGGTGGAAAAGCAGCTACAGGTCATCTCAGTGCTCCAGTGGGTCCTGT

>D32

ATGAAGACCCTCATAGCCGCCTACTTCGGGGTCCTGCGCGGCGAGCGTCAGGCCGAGGCTGACCGGAGCCAGCGCTCTCACGGAGGACCTGCGCTGTCGCGCGAGGGGTCTGGGAGATGGGGCACTGGATCCAGCATCCTCTCCGCCCTCCAGGACCTCTTCTCTGTCACCTGGCTCAATAGGTCCAAGGTGGAAAAGCAGCTACAGGTCATCTCAGTGCTCCAGTGGGTCCTGTCCTTCCTTGTACTGGGAGTGGCCTGCAGTGCCATCCTCATGTACACAACCTGCTGACCACCAGGAACT

>D33

ATGAAGACCCTCATAGCCGCCTACTCCGGGGTCCTGCGCGGCGAGCGTCAGGCCGAGGCTGACCGGAGCCAGCGCTCTCACGGAGGACCTGCGCTGTCGCGCGAGGGGTCTGGGAGATGGGGCACTGGATCCAGCATCCTCTCCGCCCTCCAGGACCTCTTCTCTGTCACCTGGCTCAATAGGTCCAAGGTGGAAAAGCAGCTACAGGTCATCTCAGTGCTCCAGTGGGTCCTGTCCTTCCTTGTACTGGGAGTGGCCTGCAGTGCCATCCTCATGTACATATTCTGCACTGATTGCTGGCTCATCGCTGTGCTCTACTTCACTTGGCTGGTGTTTGACTGGAACACACCCAAGAAAGGTGGCAGGAGGTCACAGTGGGTCCGAAACTGGGCTGTGTGGCGCTACTTTCGAGACTACTTTCCCATCCAGCTGGTGAAGACACACAACCTGCTGACCACCAGGAACTATATCTTTGGATACCACCCCCATGGTATCATGGGCCTGGGTGCCTTCTGCAACTTCAGCACAGAGGCCACAGAAGTGAGCAAGAAGTTCCCAGGCATACGGCCTTACCTGGCTACACTGGCAGGCAACTTCCGAATGCCTGTGTTGAGGGAGTACCTGATGTCTGGANGTATCTGCCCTGTCAGCCGGGACACCATAGACTATTTGCTTTC

>D34

ATGAAGACCCTCATAGCCGCCTACTCCGGGGTCCTGCGCGGCGAGCGTCAGGCCGAGGCTGACCGGAGCCAGCGCTCTCACGGAGGACCTGCGCTGTCGCGCGAGGGGTCTGGGAGATGGGGCACTGGATCCAGCATCCTCTCCGCCCTCCAGGACCTCTTCTCTGTCACCTGGCTCAATAGGTCCAAGGTGGAAAAGCAGCTACAGGTCATCTCAGTGCTCCAGTGGGTCCTGTCCTTCCTTGTACTGGGAGTGGCCTGCAGTGCCATCCTCATGTACATATTCTGCACTGATTGCTGGCTCATCGCTGTGCTCTACTTCACTTGGCTGGTGTTTGACTGGAACACACCCAAGAAAGGTGGCAGGAGGTCACAGTGGGTCCGAAACTGGGCTGTGTGGCGCTACTTTCGAGACTACTTTCCCATCCAGCTGGTGAAGACACACAACCTGCTGACCACCAGGAACTATATCTTTGGATACCACCCCCATGGTATCATGGGCCTGGGTGCCTTCTGCAACTTCAGCACAGAGGCCACAGAAGTGAGCAAGAAGTTCCCAGGCATACGGCCTTACCTGGCTACACTGGCAGGCAACTTCCGAATGCCTGTGTTGAGGGAGTACCTGATGTCTGGAGGTATCTGCCCTGTCAGCCGGGACACCATAGACTATTTG

>D37

ATGAAGACCCTCATAGCCGCCTACTCCGGGGTCCTGCGCGGCGAGCGTCAGGCCGAGGCTGACCGGAGCCAGCGCTCTCACGGAGGACCTGCGCTGTCGCGCGAGGGGTCTGGGAGATGGGGCACTGGATCCAGCATCCTCTCCGCCCTCCAGGACCTCTTCTCTGTCACCTGGCTCAATAGGTCCAAGGTGGAAAAGCAGCTACAGGTCATCTCAGTGCTCCAGTGGGTCCTGTCCTTCCTTGTACTGGGAGTGGCCTGCAGTGCCATCCTCATGTACATATTCTGCACTGATTGCTGGCTCATCGCTGTGCTCTACTTCCCTTGG

>D40

ATGAAGACCCTCATAGCCGCCTACTCCGGGGTCCTGCGCGGCGAGCGTCAGGCCGAGGCTGACCGGAGCCAGCGCTCTCACGGAGGACCTGCGCTGTCGCGCGAGGGGTCTGGGAGATGGGGCACTGGATCCAGCATCCTCTCCGCCCTCCAGGACCTCTTCTCTGTCACCTGGCTCAATAGGTCCAAGGTGGAAAAGCAGCTACAGGTCATCTCAGTGCTCCAGTGGGTCCTGTCCTTCCTTGTACTGGGAGTGGCCTGCAGTGCCATCCTCATGTACATATTCTGCACTGATTGCTGGCTCATCG

>D41

ATGAAGACCCTCATAGCCGCCTACTCCGGGGTCCTGCGCGGCGAGCGTCAGGCCGAGGCTGACCGGAGCCAGCGCTCTCACGGAGGACCTGCGCTGTCGCGCGAGGGGTCTGGGAGATGGGGCACTGGATCCAGCATCCTCTCCGCCCTCCAGGACCTCTTCTCTGTCACCTGGCTCAATAGGTCCAAGGTGGAAAAGCAGCTACAGGTCATCTCAGTGCTCCAGTGGGTCCTGTCCTTCCTTGTACTGGGAGTGGCCTGCAGTGCCATCCTCATGTACATATTCTGCACTGATTGCTGGCTCATCG

>D44

ATGAAGACCCTCATAGCCGCCTACTCCGGGGTCCTGCGCGGCGAGCGTCAGGCCGAGGCTGACCGGAGCCAGCGCTCTCACGGAGGACCTGCGCTGTCGCGCGAGGGGTCTGGGAGATGGGGCACTGGATCCAGCATCCTCTCCGCCCTCCAGGACCTCTTCTCTGTCACCTGGCTCAATAGGTCCAAGGTGGAAAAGCAGCTACAGGTCATCTCAGTGCTCCAGTGGGTCCTGTCCTTCCTTGTACTGGGAGTGGCCTGCAGTGCCATCCTCATGTACATATTCTGCACTGATTGCTGGCTCATCGCTGTGCTCTACTTCACTTGGCTGGTGTTTGACTGGAACACACCCAAGAAAGGTGGCAGGAGGTCACAGTGGGTCCGAAACTGGGCTGTGTGGCGCTACTTTCGAGACTACTTTCCCATCCAGCTGGTGAAGACACACAACCTGCTGACCACCAGGAACTATATCTTTGGATACCACCCCCATGGTATCATGGGCCTGGGTGCCTTCTGCAACTTCAGCACAGAGGCCACAGAAGTGAGCAAGAAGTTCCCAGGCATACGGCCTTACCTGGCTACACTGGCAGGCAACTTCCGAATG

>D45

ATGAAGACCCTCATAGCCGCCTACTCCGGGGTCCTGCGCGGCGAGCGTCAGGCCGAGGCTGACCGGAGCCAGCGCTCTCACGGAGGACCTGCGCTGTCGCGCGAGGGGTCTGGGAGATGGGGCACTGGATCCAGCATCCTCTCCGCCCTCCAGGACCTCTTCTCTGTCACCTGGCTCAATAGGTCCAAGGTGGAAAAGCAGCTACAGGTCATCTCAGTGCTCCAGTGGGTCCTGTCCTTCCTTGTACTGGGAGTGGCCTGCAGTGCCATCCTCATGTACATATT

>D46

ATGAAGACCCTCATAGCCGCCTACTCCGGGGTCCTGCGCGGCGAGCGTCAGGCCGAGGCTGACCGGAGCCAGCGCTCTCACGGAGGACCTGCGCTGTCGCGCGAGGGGTCTGGGAGATGGGGCACTGGATCCAGCATCCTCTCCGCCCTCCAGGACCTCTTCTCTGTCACCTGGCTCAATAGGTCCAAGGTGGAAAAGCAGCTACAGGTCATCTCAGTGCTCCAGTGGGTCCTGTCCTTCCTTGTACTGGGAGTGGCCTGCTTTCAAAGAATGGGAGTGGCAATGCTATCATCATCGTGGTCGGGGGTGCGGCTGAGTCTCTGAGCTCCATGCCTGGCAAGAATGCAGTCACCCTG

>D47

ATGAAGACCCTCATAGCCGCCTACTCCGGGGTCCTGCGCGGCGAGCGTCAGGCCGAGGCTGACCGGAGCCAGCGCTCTCACGGAGGACCTGCGCTGTCGCGCGAGGGGTCTGGGAGATGGGGCACTGGATCCAGCATCCTCTCCGCCCTCCAGGACCTCTTCTCTGTCACCTGGCTCAA

>D49

ATGAAGACCCTCATAGCCGCCTACTCCGGGGTCCTGCGCGGCGAGCGTCAGGCCGAGGCTGACCGGAGCCAGCGCTCTCACGGAGGACCTGCGCTGTCGCGCGAGGGGTCTGGGAGATGGGGCACTGGATCCAGCATCCTCTCCGCCCTCCAGGACCTCTTCTCTGTCACCTGGCTCAATAGGTCC

>D53

ATGAAGACCCTCATAGCCGCCTACTCCGGGGTCCTGCGCGGCGAGCGTCAGGCCGAGGCTGACCGGAGCCAGCGCTCTCACGGAGGACCTGCGCTGTCGCGCGAGGGGTCTGGGAGATGGGGCACTGGATCCAGCATCCTCTCCGCCCTCCAGGACCTCTTCTCTGTCACCTGGCTCAATAGGTCCAAGGTGGAAAAGCAGCTACAGGTCATCTCAGTGCTCCAGTGGGTCCTGTCCTTCCTTGTACTGGGAGTGGCCTGCAGTGCCATCCTCATGTACATATTCTGCACTGATTGCTGGCTCATCGCTGTGCTCTACTTCACTTGGCTGGTGTTTGACTGGAACACACCCAAGAAAGGTGGCAGGAGGTCACAGTGGGTCCGAAACTGGGCTGTGTGGCGCTACTTTCGAGACTACTTTCCCATCCAGCTGGTGAAGACACACAACCTGCTGACCACCAGGAACTATATCTTTGGATACCACCCCCATGGTATCATGGGCCTGGGTGCCTTCTGCAACTTCAGCACAGAGGCCACAGAAGTGAGCAAGAAGTTCCCAGGCATACGG

>D54

ATGAAGACCCTCATAGCCGCCTACTCCGGGGTCCTGCGCGGCGAGCGTCAGGCCGAGGCTGACCGGAGCCAGCGCTCTCACGGAGGACCTGCGCTGTCGCGCGAGGGGTCTGGGAGATGGGGCACTGGATCCAGCATCCTCTCCGCCCTCCAGGACCTCTTCTCTGTCACCTGGCTCAATAGGTCCAAGGTGGAAAAGCAGCTACAGGTCATCTCAGTGCTCCAGTGGGTCCTGTCCTTCCTTGTACTGGGAGTGGCCTGCAGTTCCATCCTCATGTACATATTCTGCACTGATTGCTGGCTCATCGCTGTGCTCTACTTCACTTGGCTGGTGTTTGACTGGAACACACCCAAGAAAGGTGGCAGGAGGTCACAGTGGGTCCGAAACTGGGCTGTGTGGCGCTA

>D55

ATGAAGACCCTCATAGCCGCCTACTCCGGGGTCCTGCGCGGCGAGCGTCAGGCCGAGGCTGACCGGAGCCAGCGCTCTCACGGAGGACCTGCGCTGTCGCGCGAGGGGTCTGGGAGATGGGGCACTGGATCCAGCATCCTCTCCGCCCTCCAGGACCTCTTCTCTGTCACCTGGCTCAATAGGTCCAAGGTGGAAAAGCAGCTACAGGTCATCTCAGTGCTCCAGTGGGTCCTGTCCTTCCTTGTACTGGGAGTGGCCTGCAGTGCCATCCTCATGTACATATTCTGCACTGATTGCTGGCTCATCG

>D58

ATGAAGACCCTCATAGCCGCCTACTCCGGGGTCCTGCGCGGCGAGCGTCAGGCCGAGGCTGACCGGAGCCAGCGCTCTCACGGAGGACCTGCGCTGTCGCGCGAGGGGTCTGGGAGATGGGGCACTGGATCCAGCATCCTCTCCGCCCTCCAGGACCTCTTCTCTGTCACCTGGCTCAATAGGTCCAAGGTGGAAAAG

>D59

ATGAAGACCCTCATAGCCGCCTACTCCGGGGTCCTGCGCGGCGAGCGTCAGGCCGAGGCTGACCGGAGCCAGCGCTCTCACGGAGGACCTGCGCTGTCGCGCGAGGGGTCTGGGAGATGCACTGGATCCAGCATCCTCTCCGCCCTCCAGGACCTCTTCTCTGTCACCTGGCTCAATAGGTCCAAGGTGGAAAAGCAGCTACAGGTCATCTCAGTGCTCCAGTGGGTCCTGTCCTTCCTTGTACTGGGAGTGGCCTGCAGTGCCATCCTCATGTACATATTCTGCACTGATTGCTGGCTCATCGCTGTGCTCTACTTCACTTGGCTGGTGTTTGACTGGAACACACCCAAGAAAGGTGGCAGGAGGTCACAGTGGGTCCGAAACTGGGCTGTGTGGCGCTACTTTCGAGACTACTTTCCCATCCAGCTGGTGAAGACACACAACCTGCTGACCACCAGGAACTATATCTTT

>D60

ATGAAGACCCTCATAGCCGCCTACTCCGGGGTCCTGCGCGGCGAGCGTCAGGCCGAGGCTGACCGGAGCCAGCGCTCTCACGGAGGACCTGCGCTGTCGCGCGAGGGGTCTGGGAGATGGGGCACTGGATCCAGCATCCTCTCCGCCCTCCAGGACCTCTTCTCTGTCACCTGGCTCAATAGGTCCAAGGTGGAAAAGCAGCTACAGGTCATCTCAGTGCTCCAGTGGGTCCTGTCCTTCCTTGTACTGGGAGTGGCCTGCAGTGCCATCCTCATGTACATATTCTGCACTGATTGCTGGCTCATCGCTGTGCTCTACTTCACTTGGCTGGTGTTTGACTGGAACACACCCAAGAAAGGTGGCAGGAGGTCACAGTGGG

>D62

ATGAAGACCCTCATAGCCGCCTACTCCGGGGTCCTGCGCGGCGAGCGTCAGGCCGAGGCTGACCGGAGCCAGCGCTCTCACGGAGGACCTGCGCTGTCGCGCGAGGGGTCTGGGAGATGGGGCACTGGATCCAGCATCCTCTCCGCCCTCCAGGACCTCTTCTCTGTCACCTGGCTCAATAGGTCCAAGGTGGAAAAGCAGCTACAGGTCATCTCAGTGCTCCAGTGGGTCCTGTCCTTCCTTGTACTGGGAGTGGCCTGCAGTGCCATACGGCCTTACCTGGCTACACTGGCAGGCAACTTCCGAATG

>D63

ATGAAGACCCTCATAGCCGCCTACTCCGGGGTCCTGCGCGGCGAGCGTCAGGCCGAGGCTGACCGGAGCCAGCGCTCTCACGGAGGACCTGCGCTGTCGCGCGAGGGGTCTGGGAGATGGGGCACTGGATCCAGCATCCTCTCCGCCCTCCAGGACCTCTTCTCTGTCACCTGGCTCAATAGGTCCAAGGTGGAAAAGCAGCTACAGGTCATCTCAGTGCTCCAGTGGGTCCTGTCCTTCCTTGTACTGGGAGTGGCCTGCAGTGCCATCCTCATGTACATATTCTGCACTGATTGCTGGCTCATCGCTGTGCTCT

>D65

ATGAAGACCCTCATAGCCGCCTACTCCGGGGTCCTGCGCGGCGAGCGTCAGGCCGAGGCTGACCGGAGCCAGCGCTCTCACGGAGGACCTGCGCTGTCGCGCGAGGGGTCTGGGAGATGGGGCACTGGATCCAGCATCCTCTCCGCCCTCCAGGACCTCTTCTCTGTCACCTGGCTCAATAGGTCCAAGGTGGAAAAGCAGCTACAGGTCATCTCAGTGCTCCAGTGGGTCCTGTCCTTCCTTGTACTGGGAGTGGCCTGCAGTGCCATCCTCATGTACATATTCTGCACTGATTGCTGGCTCATCG

>D66

ATGAAGACCCTCATAGCCGCCTACTCCGGGGTCCTGCGCGGCGAGCGTCAGGCCGAGGCTGACCGGAGCCAGCGCTCTCACGGAGGACCTGCGCTGTCGCGCGAGGGGTCTGGGAGATGGGGCACTGGATCCAGCATCCTCTCCGCCCTCCAGGACCTCTTCTCTGTCACCTGGCTCAATAGGTCCAAGGTGGAAAAGCAGCTACAGGTCATCTCAGTGCTCCAGTGGGTCCTGTCCTTCCTTGTACTGGGAGTGGCCTGCAGTGCCATCCTCATGTACATATTCTGCACTGNATGATGGCTCATCACAGNGCTCTACTTCACTTGGCTGGTGTTTGACTGGAACACNNNAAAGAAACGTGGCAGGAGGANGCANTGGGTCCGAAACTGGGCTGTGTGGCGCTACTTTNGANACTACTTTCNCATCCNNCTGNNGANGACAC

>D68

ATGAAGACCCTCATAGCCGCCTACTCCGGGGTCCTGCGCGGCGAGCGTCAGGCCGAGGCTGACCGGAGCCAGCGCTCTCACGGAGGACCTGCGCTGTCGCGCGAGGGGTCTGGGAGATGGGGCACTGGATCCAGCATCCTCTCCGCCCTCCAGGACCTCTTCTCTGTCACCTGGCTCAATAGGTCCAAGGTGGAAAAGCAGCTACAGGTCATCTCAGTGCTCCAGTGGGTCCTGTCCTTCCTTGTACTGGGAGTGGCCTGCAGTGCCATCCTCATGTACATATTCTGCACTGATTGCTGGCTCATCGCTGTGCTCTACTTCACTTGGCTGGTGTTTGACTGGAACACACCCAAGAAAGGTGGCAGGAGGTCACAGTGGGTCCGAAACTGGGCTGTGTGGCGCTACTTTCGAGACTACTTTCCCATCCAGCTGGTGAAGACACACAACCTGCTGACCACCAGGAACTATATCTTTGGATACCACCCCCATGGTATCATGGGCCTGGGTGCCTTCTG

>D69

ATGAAGACCCTCATAGCCGCCTACTCCGGGGTCCTGCGCGGCGAGCGTCAGGCCGAGGCTGACCGGAGCCAGCGCTCTCACGGAGGACCTGCGCTGTCGCGCGAGGGGTCTGGGAGATGGGGCACTGGATCCAGCATCCTCTCCGCCCTCCAGGACCTCTTCTCTGTCACCTGGCTCAATAGGTCCAAGGTGGAAAAGCAGCTACAGGTCATCTCAGTGCTCCAGTGGGTCCTGTCCTTCCTTGTACTGGGAGTGGCCTG

>D70

ATGAAGACCCTCATAGCCGCCTACTCCGGGGTCCTGCGCGGCGAGCGTCAGGCCGAGGCTGACCGGAGCCAGCGCTCTCACGGAGGACCTGCGCTGTCGCGCGAGGGGTCTGGGAGATGGGGCACTGGATCCAGCATCCTCTCCGCCCTCCAGGACCTCTTCTCTGTCACCTGGCTCAATAGGTCCAAGGTGGAAAAGCAGCTACAGGTCATCTCAGTGCTCCAGTGGGTCCTGTCCTTCCTTGTACTGGGAGTGGCCTGCAGTGCCATCCTCATGTACATATTCTGCACTGATTGCTGGCTCATCGCTGTGCTCTACTTCACTTGGCTGGTGTTTGACTGGAACACACCCAAGAAAGGTGGCAGGAGGTCACAGTGGGTCCGAAACTGGGCTGTGTGGCGCTACTTTCGAGACTACTTTCCCATCCAGCTGGTGAAGACACACAACCTGCTGACCACCAGGAACTATATCTTTGGATACCACCCCCATGGTATCATGGGCCTGGGTGCCTTCTGCAACTTCAGCACAGAGGCCACAGAAGTGAGCAAGAAGTTCCCAGGCATACGGCCTTACCTGGCTACACTGGCAGGCAACTTCCGAATG

>D71

ATGAAGACCCTCATAGCCGCCTACTCCGGGGTCCTGCGCGGCGAGCGTCAGGCCGAGGCTGACCGGAGCCAGCGCTCTCACGGAGGACCTGCGCTGTCGCGCGAGGGGTCTGGGAGATGGGGCACTGGATCCAGCATCCTCTCCGCCCTCCAGGACCTCTTCTCTGTCACCTGGCTCAATAGGTCCAAGGTGGAAAAGCAGCTACAGGTCATCTCAGTGCTCC

>D73

ATGAAGACCCTCATAGCCGCCTACTCCGGGGTCCTGCGCGGCGAGCGTCAGGCCGAGGCTGACCGGAGCCAGCGCTCTCACGGAGGACCTGCGCTGTCGCGCGAGGGGTCTGGGAGATGGGGCACTGGATCCAGCATCCTCTCCGCCCTCCAGGACCTCTTCTCTGTCACCTGGCTCAATAGGTCCAAGGTGGAAAAGCAGCTACAGGTCATCTCAGTGCTCCAGTGGGTCCTGTCCTTCCTTGTACTGGGAGTGGCCTG

>D74

ATGAAGACCCTCATAGCCGCCTACTCCGGGGTCCTGCGCGGCGAGCGTCAGGCCGAGGCTGACCGGAGCCAGCGCTCTCACGGAGGACCTGCGCTGTCGCGCGAGGGGTCTGGGAGATGGGGCACTGGATCCAGCATCCTCTCCGCCCTCCAGGACCTCTTCTCTGTCACCTGGCTCAATAGGTCCAAGGTGGAAAAGCAGCTACAGGTCATCTCAGTGCTCCAGTGGGTCCTGTCCTTCCTTGTACTGGGAGTGGCCTGCAGTGCCATCCTCATGTACATATTCTGCACTGATTGCTGGCTCATCGCTGTG

>D76

ATGAAGACCCTCATAGCCGCCTACTCCGGGGTCCTGCGCGGCGAGCGTCAGGCCGAGGCTGACCGGAGCCAGCGCTCTCACGGAGGACCTGCGCTGTCGCGCGAGGGGTCTGGGAGATGGGGCACTGGATCCAGCATCCTCTCCGCCCTCCAGGACCTCTTCTCTGTCACCTGGCTCAATAGGTCCAAGGTGGAAAAGCAGCTACAGGTCATCTCAGTGCTCCAGTGGGTCCTGTCCTTCCTTGTACTGGGAGTGGCCTGCAGTGCCATCCTCATGTACATATTCTGCACTGATTGCTGGCTCATCGCTGTGCTCTACTTCACTTGGCTGGTGTTTGACTGGAACACACCCAAGAAAGGTGGCAGGAGGTCACAGTGGGTCCGAAACTGGGCTGTGTGGCGCTACTTTCGAGACTACTTTCCCATCCAGCTGGTGAAGACACACAACCTGCTGACCACCAGGAACTATATCTTTGGATACCACCCCCATGGTATCATGGGCCTGGGTGCCTTCTGCAACTTCAGCACAGAGGCCACAGAAGTGAGCAAGAAGTTCCCAGGCATACGGCCTTACCTGGCTACACTGGCAGGCAACTTCCGAATGCCTGTGTTGANGGAGTACCTGATGTCTGGANGTATCTGCCCTGTCAGCCGGGACACCATAGACTATTTGCTTTCAAAGAATGGGAGTGGCAATGCTATCATCATCGTGGTCGGGGGTGCGGCTGAGTCTCTGAGCTCCATGCCTGGCAAGAATG

>D78

ATGAAGACCCTCATAGCCGCCTACTCCGGGGTCCTGCGCGGCGAGCGTCAGGCCGAGGCTGACCGGAGCCAGCGCTCTCACGGAGGACCTGCGCTGTCGCGCGAGGGGTCTGGGAGATGGGGCACTGGATCCAGCATCCTCTCCGCCCTCCAGGACCTCCTTTCTGTCACCTGGCTCAATAGGTCCAAGGTGGAAAAGCAGCTACAGGTCATCTCAGTGCTCCAGTGGGTCCTGTCCTTCCTTGTACTGGGAGTGGCCTGCAGTGCCATCCTCATGTACATATTCTGC

>D79

ATGAAGACCCTCATAGCCGCCTACTCCGGGGTCCTGCGCGGCGAGCGTCAGGCCGAGGCTGACCGGAGCCAGCGCTCTCACGGAGGACCTGCGCTGTCGCGCGAGGGGTCTGGGAGATGGGGGCACTGGATCCAGCATCCTCTCCGCCCTCCAGGACCTCTTCTCTGTCACCTGGCTCAATAGGTCCAAGGTGGAAAAGCAGCTACAGGTCATCTCAGTGCTCCAGTGGGTCCTGTCCTTCCTTGTACTGGGAGTGGCCTGCAGTGCCATCCTCATGTACATATTCTGCACTGATTGCTGGCTCATCGCTGTGCTCTACTT

>D80

ATGAAGACCCTCATAGCCGCCTACTCCGGGGTCCTGCGCGGCGAGCGTCAGGCCGAGGCTGACCGGAGCCAGCGCTCTCACGGAGGACCTGCGCTGTCGCGCGAGGGGTCTGGGAGATGGGGCACTGGATCCAGCATCCTCTCCGCCCTCCAGGACCTCTTCTCTGTCACCTGGCTCAATAGGTCCAAGGTGGAAAAGCAGCTACAGGTCATCTCAGTGCTCCAGTGGGTCCTGTCCTTCCTTGTACTGGGAGTGGCCTGCAGTGCCATCCTCATGTACATATTCTGCACTGATTGCTGGCTCATCGCTGTGCTCTACTTCACTTGGCTGGTGTTTGACTGGAACACACCCAAGAAAGGTGGCAGGAGGTCACAGTGGGTCCGAAACTGGGCTGTGTGGCGCTACTTTCGAGACTACTTTCCCATCCAGCTGGTGAAGACACACAACCTGCTGACCACCAGGAACTATATCTTTGGATACCACCCCCATGGTATCATGGGCCTGGGTGCCTTCTGCAACTTCAGCACAGAGGCCACAGAAGTGAGCAAGAAGTTCCCAGGCATACGG

>D81

ATGAAGACCCTCATAGCCGCCTACTCCGGGGTCCTGCGCGGCGAGCGTCAGGCCGAGGCTGACCGGAGCCAGCGCTCTCACGGAGGACCTGCGCTGTCGCGCGAGGGGTCTGGGAGATGGGGCACTGGATCCAGCATCCTCTCCGCCCTCCAGGACCTCTTCTCTGTCACCTGGCTCAATAGGTCCAAGGTGGAAAAGCAGCTACAGGTCATCTCAGTGCTCCAGTGGGTCCTGTCCTTCCTTGTACTGGGAGTGGCCTG

>D82

ATGAAGACCCTCATAGCCGCCTACTCCGGGGTCCTGCGCGGCGAGCGTCAGGCCGAGGCTGACCGGAGCCAGCGCTCTCACGGAGGACCTGCGCTGTCGCGCGAGGGGTCTGGGAGATGGGGCACTGGATCCAGCATCCTCTCAGGTGATCTTCGAGGAGGGCTCCTGGGACCGATGGGTCCAGAAGAAGTTCCAGAAATACATTGGTTTCGCCCCATGCATCTTCCATGGTCGAGGCCTCTTCTCCTCCGACACCTGGGGGCTGGTGCCCTACTCCAAGCCCATCACCACTGTTGTGGGAGAGCCCATCACCATCCCCAAGCTGGAGCACCCAACCCAGCAAGACATCGACCTGTACCACACCATGTACATGGAGGCCCTGGTGAAGCTCTTCGACAAGCACAAGACCAAGTTCGGCCTCCCGGAGACTGAGGTCCTGGAGGTGAACTGAAATCACTAGTGCGGCCGCCTGCCGCCCTCCAGGACCTCTTCTCTGTCACCTGGCTCAATAGGTCCAAGGTGGAAAAGCAGCTACAGGTCATCTCAGTGCTCCAGTGGGTCCTGTCCTTCCTTGTACTGGGAGTGGCCTG

>D83

ATGAAGACCCTCATAGCCGCCTACTCCGGGGTCCTGCGCGGCGAGCGTCAGGCCGAGGCTGACCGGAGCCAGCGCTCTCACGGAGGACCTGCGCTGTCGCGCGAGGGGTCTGGGAGATGGGGCACTGGATCCAGCATCCTCTCCGCCCTCCAGGACCTCTTCTCTGTCACCTGGCTCAATAGGTCCAAGGTGGAAAAG

>D84

ATGAAGACCCTCATAGCCGCCTACTCCGGGGTCCTGCGCGGCGAGCGTCAGGCCGAGGCTGACCGGAGCCAGCGCTCTCACGGAGGACCTGCGCTGTCGCGCGAGGGGTCTGGGAGATGGGGCACTGGATCCAGCATCCTCTCCGCCCTCCAGGACCTCTTCTCTGTCACCTGGCTCAATAGGTCCAAGGTGGAAAAGCAGCTACAGGTCATCTCAGTGCTCCAGTGGGTCCTGTCCTTCCTTGTACTGGGAGTGGCCTGCAGTGCCATCCTCATGTACATATTCTGCACTGATTGCTGGCTCATCGCTGTGCTCTACTTCACTTGGCTGGTGTTTGACTGGAACACACCCAAGAAAGGTGGCAGGAGGTCACAGTGGGTCCGAAACTGGGCTGTGTGGCGCTACTTTCGAGACTACTTTCCCATCCAGCTGGTGAAGACACACAACCTGCTGACCACCAGGAACTATATCTTTGGATACCACCCCCATGGTATCATGGGCCTGGGTGCCTTCTG

>D86

ATGAAGACCCTCATAGCCGCCTACTCCGGGGTCCTGCGCGGCGAGCGTCAGGCCGAGGCTGACCGGAGCCAGCGCTCTCACGGAGGACCTGCGCTGTCGCGCGAGGGGTCTGGGAGATGGGGCACTGGATCCAGCATCCTCTCCGCCCTCCAGGACCTCTTCTCTGTCACCTGGCTCAATAGGTCCAAGGTGGAAAAGCAGCTACAGGTCATCTCAGTGCTCCAGTGGGTCCTGTCCTTCCTTGTACTGGGAGTGGCCTGCAGTGCCATCCTCATGTACATATTCTGCACTGATTGCTGGCTCATCGCTGTGCTCTACTTCACTTGGCTGGTGTTTGACTGGAACACACCCAAGAAAGGTGGCAGGAGGTCACAGTGGGTCCGAAACTGGGCTGTGTGGCGCTACTTTCGAGACTACTTTCCCATCCAGCTGGTGAAGACACACAACCTGCTGACCACCAGGAACTATATCTTTGGATACCACCCCCATGGTATCATGGGCCTGGGTGCCTTCTGCAACTTCAGCACAGAGGCCACAGAAGTGAGCAAGAAGTTCCCAGGCATACGGCCTTACCTGGCTACACTGGCAGGCAACTTCCGAATGCCTGTGTTGAGGGAGTACCTGATGTCTGGAGGTATCTGCCCTGTCAGCCGGGACACCATAGACTATTTG

>D87

ATGAAGACCCTCATAGCCGCCTACTCCGGGGTCCTGCGCGGCGAGCGTCAGGCCGAGGCTGACCGGAGCCAGCGCTCTCACGGAGGACCTGCGCTGTCGCGCGAGGGGTCTGGGAGATGGGGCACTGGATCCAGCATCCTCTCCGCCCTCCAGGACCTCTTCTCTGTCACCTGGCTCAATAGGTCCAAGGTGGAAAAGCAGCTACAGGTCATCTCAGTGCTCCAGTGGGTCCTGTCCTTCCTTGTACTGGGAGTGGCCTGCAGTGCCATCCTCATGTACATATTCTGCACTGATTGCTGGCTCATCGCTGTGCTCTACTTCACTTGGCTGGTGTTTGACTGGAACACACCCAAGAAAGGTGGCAGGAGGTCACAGTGGGTCCGAAACTGGGCTGTGTGGCGCTACTTTCGAGACTACTTTCCCATCCAGCTGGTGAAGACACACAACCTGCTGACCACCAGGAACTATATCTTTGGATACCACCCCCATGGTATCATGGGCCTGGGTGCCTTCTGCAACTTCAGCACAGAGGCCACAGAAGTGAGCAAGAAGTTCCCAGGCATACGGCCTTACCTGGCTACACTGGCAGGCAACTTCCGAATGCCTGTGTTGAGGGAGTACCTGATGTCTGGAGGTATCTGCCCTGTCAGCCGGGACACCATAGACTATTTGCTTTCAAAGAATGGGAGTGGCAATGCTATCATCATCGTGGTCGGGGGTGCGG

>D88

ATGAAGACCCTCATAGCCGCCTACTCCGGGGTCCTGCGCGGCGAGCGTCAGGCCGAGGCTGACCGGAGCCAGCGCTCTCACGGAGGACCTGCGCTGTCGCGCGAGGGGTCTGGGAGATGGGGCACTGGATCCAGCATCCTCTCCGCCCTCCAGGACCTCTTCTCTGTCACCTGGCTCAATAGGTCCAAGGTGGAAAAGCAGCTACAGGTCATCTCAGTGCTCCAGTGGGTCCTGTCCTTCCTTGTACTGGGAGTGGCCTGCAGTGCCATCCTCATGTACATATTCTGCACTGATTGCTGGCTCATCGCTGTGCTCTACTTCACTTGGCTGGTGTTTGACTGGAACACACCCAAGAAAGGTGGCAGGAGGTCACAGTGGGTCCGAAACTGGGCGGTGTGGCGCTACTTTCGAGACTACTTTCCCATCCAGCTGGTGAAGACACACAACCTGCTGACCACCAGGAACTATATCTTTGGATACCACCCCCATGGTATCATGGGCCTGGGTGCCTTCTGCAACTTCAGCACAGAGGCCACAGAAGTGAGCAAGAAGTTCCCAGGCATACGGCCTTACCTGGCTACACTGGCAGGCAACTTCCGAATGCCTGTGTTGAGGGAGTACCTGATGTCTGGANGTATCTGCCCTGTCAGCCGGGACACCATAGACTATTTGCTTTCAAAGAATGGGAGTGGCAATGCTATCATCATCGTGGTCGGGGGTGCGGCTGAGTCTCTGAGCTCCATGCCTGGNAAGAATG

>D89

ATGAAGACCCTCATAGCCGCCTACTCCGGGGTCCTGCGCGGCGAGCGTCAGGCCGAGGCTGACCGGAGCCAGCGCTCTCACGGAGGACCTGCGCTGTCGCGCGAGGGGTCTGGGAGATGGGGCACTGGATCCAGCATCCTCTCCGCCCTCCAGGACCTCTTCTCTGTCACCTGGCTCAATAGGTCCAAGGTGGAAAAGCAGCTACAGGTCATCTCAGTGCTCCAGTGGGTCCTGTCCTTCCTTGTACTGGGAGTGGCCTGCAGTGCCATCCTCATGTACATATTCTGCACTGATTGCTGGCTCATCGCTGTGCTCTACTTCACTTGG

>D90

ATGAAGACCCTCATAGCCGCCTACTCCGGGGTCCTGCGCGGCGAGCGTCAGGCCGAGGCTGACCGGAGCCAGCGCTCTCACGGAGGACCTGCGCTGTCGCGCGAGGGGTCTGGGAGATGGGGCACTGGATCCAGCATCCTCTCCGCCCTCCAGGACCTCTTCTCTGTCACCTGGCTCAATAGGTCCAAGGTGGAAAAGCAGCTACAGGTCATCTCAGTGCTCCAGTGGGTCCTGTCCTTCCTTGTACTGGGAGTGGCCTGCAGTGCCATCCTCATGTACATATTCTGCACTGATTGCTGGCTCATCGCTGTGCTCTACTTCACTTGGCTGGTGTTTGACTGGAACACACCCAAGAAAGGTGG

>D91

ATGAAGACCCTCATAGCCGCCTACTCCGGGGTCCTGCGCGGCGAGCGTCAGGCCGAGGCTGACCGGAGCCAGCGCTCTCACGGAGGACCTGCGCTGTCGCGCGAGGGGTCTGGGAGATGGGGCACTGGATCCAGCATCCTCTCCGCCCTCCAGGACCTCTTCTCTGTCACCTGGCTCAATAGGTCCAAGGTGGAAAAGCAGCTACAGGTCATCTCAGTGCTCCAGTGGGTCCTGTCCTTCCTTGTACTGGGAGTGGCCTGCAGTGCCATCCTCATGTACATATTCTGCACTGATTGCTGGCTCATCGCTGTGCTCTACTTCACTTGGCTGGTGTTTGACTGGAACACACCCAAGAAAGGTGGCAGGAGGTCACAGTGGGTCCGAAACTGGGCTGTGTGGCGCTACTTTCGAGACTACTTTCCCATCCAGCTGGTGAAGACACACAACCTGCTGACCACCAGGAACTATATCTTTGGATACCACCCCCATGGTATCATGGGCCTGGGTGCCTTCTGCAACTTCAGCACAGAGGCCACAGAAGTGAGCAAGAAGTTCCCAGGCATACGG

>D93

ATGAAGACCCTCATAGCCGCCTACTCCGGGGTCCTGCGCGGCGAGCGTCAGGCCGAGGCTGACCGGAGCCAGCGCTCTCACGGAGGACCTGCGCTGTCGCGCGAGGGGTCTGGGAGATGGGGCACTGGATCCAGCATCCTCTCCGCCCTCCAGGACCTCTTCTC

>D94

ATGAAGACCCTCATAGCCGCCTACTCCGGGGTCCTGCGCGGCGAGCGTCAGGCCGAGGCTGACCGGAGCCAGCGCTCTCACGGAGGACCTGCGCTGTCGCGCGAGGGGTCTGGGAGATGGGGCACTGGATCCAGCATCCTCTCCGCCCTCCAGGACCTCTTCTCTGTCACCTGGCTCAATAGGTCCAAGGTGGAAAAGCAGCTACAGGTCATCTCAGTGCTCCAGTGGGTCCTGTCCTTCCTTGTACTGGGAGTGGCCTGCAGTGCCATCCTCATGTACATATTCTGCACTGATTGCTGGCTCATCGCTGTGCTCTACTTCACTTGGCTGGTGTTTGACTGGAACACACCCAAGAAAGGTGGCAGGAGGTCACAGTGGGTCCGAAACTGGGCTGTGTGGCGCTACTTTCGAGACTACTTTCCCATCCAGCTGGTGAAGACACACAACCTGCTGACCACCAGGAACTATATCTTTGGATACCACCCCCATGGTATCATGGGCCTGGGTGCCTTCTGCAACTTCAGCACAGAGGCCACAGAAGTGAGCAAGAAGTTCCCAGGCATACGG

>D95

ATGAAGACCCTCATAGCCGCCTACTCCGGGGTCCTGCGCGGCGAGCGTCAGGCCGAGGCTGACCGGAGCCAGCGCTCTCACGGAGGACCTGCGCTGTCGCGCGAGGGGTCTGGGAGATGGGGCACTGGATCCAGCATCCTCTCCGCCCTCCAGGACCTCTTCTCTGTCACCTGGCTCAATAGGTCCAAGGTGGAAAAGCAGCTACAGGTCATCTCAGTGCTCCAGTGGGTCCTGTCCTTCCTTGTACTGGGAGTGGCCTGCAGTGCCATCCTCATGTACATATTTGCACTGATTGCTGGCTCATCG

>D96

ATGAAGACCCTCATAGCCGCCTACTCCGGGGTCCTGCGCGGCGAGCGTCAGGCCGAGGCTGACCGGAGCCAGCGCTCTCACGGAGGACCTGCGCTGTCGCGCGAGGGGTCTGGGAGATGGGGCACTGGATCCAGCATCCTCTCCGCCCTCCAGGACCTCTTCTCTGTCACCTGGCTCAATAGGTCCAAGGTGGAAAAGCAGCTACAGGTCATCTCAGTGCTCCAGTGGGTCCTGTCCTTCCTTGTACTGGGAGTGGCCTGCAGTGCCATCCTCATGTACATATTCTGCACTGATTGCTGGCTCATCGCTGTGCTCTACTTCAACTTCAGCACAGAGGCCAC
