# Supplementary material for: Decoding non-canonical mRNA decay by the endoplasmic-reticulum stress sensor IRE1α
Source: Nat Commun. 2021 Dec 15;12:7310. doi: 10.1038/s41467-021-27597-7 (PMC8674358; doi:10.1038/s41467-021-27597-7)
Supplement: Supplementary file 5 — Reporting Summary [file 41467_2021_27597_MOESM5_ESM.pdf]

nature portfolio

Corresponding author(s): Thomas D. Wu, Peter Walter, and Avi Ashkenazi

Last updated by author(s): Oct 18, 2021

Reporting Summary

Nature Portfolio wishes to improve the reproducibility of the work that we publish. This form provides structure for consistency and transparency in reporting. For further information on Nature Portfolio policies, see our Editorial Policies and the Editorial Policy Checklist.

Statistics

For all statistical analyses, confirm that the following items are present in the figure legend, table legend, main text, or Methods section.

n/a	Confirmed	
☐	☒	The exact sample size (n) for each experimental group/condition, given as a discrete number and unit of measurement
☐	☒	A statement on whether measurements were taken from distinct samples or whether the same sample was measured repeatedly
☐	☒	The statistical test(s) used AND whether they are one- or two-sided<br>*Only common tests should be described solely by name; describe more complex techniques in the Methods section.*
☒	☐	A description of all covariates tested
☒	☐	A description of any assumptions or corrections, such as tests of normality and adjustment for multiple comparisons
☐	☒	A full description of the statistical parameters including central tendency (e.g. means) or other basic estimates (e.g. regression coefficient) AND variation (e.g. standard deviation) or associated estimates of uncertainty (e.g. confidence intervals)
☐	☒	For null hypothesis testing, the test statistic (e.g. F, t, r) with confidence intervals, effect sizes, degrees of freedom and P value noted<br>*Give P values as exact values whenever suitable.*
☒	☐	For Bayesian analysis, information on the choice of priors and Markov chain Monte Carlo settings
☒	☐	For hierarchical and complex designs, identification of the appropriate level for tests and full reporting of outcomes
☒	☐	Estimates of effect sizes (e.g. Cohen's d, Pearson's r), indicating how they were calculated

Our web collection on statistics for biologists contains articles on many of the points above.

Software and code

Policy information about availability of computer code

Data collection	Image data for western blots were acquired using Azure c600 Imaging system. RNA agarose gels were visualized using a BioRad Molecular Imager ChemiDoc ZRS+. RT-qPCR were acquired using the ABI QuantStudio 7 Flex Real-Time PCR system. Luminescence was acquired using an Envision system from PerkinElmer. Refseq transcripts for analysing RIDD sites were obtained with the biomartr package (version 0.9.2) in R, using the getRNA function for organisms "Homo sapiens" and "Mus musculus".
Data analysis	edgeR package (version 3.24.3), R (version 3.5.1 & 4.0.0), limma package (version 3.38.3), GMAP (version 2019-12-01), signalp (version 3.0),survival package (version 2.44-1.1), Graphpad Prism, Microsoft Excel, gRIDD program is available as Supplementary File as part of this paper.

For manuscripts utilizing custom algorithms or software that are central to the research but not yet described in published literature, software must be made available to editors and reviewers. We strongly encourage code deposition in a community repository (e.g. GitHub). See the Nature Portfolio guidelines for submitting code & software for further information.

Data

Policy information about availability of data

All manuscripts must include a data availability statement. This statement should provide the following information, where applicable:

- Accession codes, unique identifiers, or web links for publicly available datasets
- A description of any restrictions on data availability
- For clinical datasets or third party data, please ensure that the statement adheres to our policy

Protein structures overlay of human B2B dimers onto oligomeric S. cerevisiae IRE1 are from the Protein Data Bank with respective accession codes: 6W3C, and 3FBV. Raw sequences and processed data that support the findings of this study are now available at NCBI GEO as GSE169585.

Field-specific reporting

Please select the one below that is the best fit for your research. If you are not sure, read the appropriate sections before making your selection.

- [x] Life sciences
- [ ] Behavioural & social sciences
- [ ] Ecological, evolutionary & environmental sciences

For a reference copy of the document with all sections, see nature.com/documents/nr-reporting-summary-flat.pdf

Life sciences study design

All studies must disclose on these points even when the disclosure is negative.

Sample size	we determined the sample size based on similar experimental setups in previous publications.
Data exclusions	no data were excluded
Replication	Samples were replicated in duplicates or triplicates in a single experiment as stated in the methods. Every experiment was repeated a minimum of two separate times.
Randomization	Only biochemical or cell-based experiments (no animals) were carried out in this study and as such there was no need for randomization of our samples.
Blinding	Only biochemical or cell-based experiments (no animals) were carried out in this study and as such there was no need for randomization of our samples.

Reporting for specific materials, systems and methods

We require information from authors about some types of materials, experimental systems and methods used in many studies. Here, indicate whether each material, system or method listed is relevant to your study. If you are not sure if a list item applies to your research, read the appropriate section before selecting a response.

Materials & experimental systems

n/a	Involved in the study	
☐	☒	Antibodies
☐	☒	Eukaryotic cell lines
☒	☐	Palaeontology and archaeology
☒	☐	Animals and other organisms
☒	☐	Human research participants
☒	☐	Clinical data
☒	☐	Dual use research of concern

Methods

n/a	Involved in the study	
☒	☐	ChIP-seq
☒	☐	Flow cytometry
☒	☐	MRI-based neuroimaging

Antibodies

Antibodies used	IRE1α (#3294), TGOLN2 (#95649), AIM2 (#12948), Actin (#3700), and GAPDH (#8884) from Cell Signaling Technology. CD59 (#133707), GBA (#125065), BCAM (#134110), HIP1 (#181238), TLR2 (#68159), SIX2 (#111827), SUOX (#129094), and BMP4 (#124715) from Abcam. BLOC1S1 (#19687-1-AP), OAS2 (#19279-1-AP), ALDH1A3 (#25167-1-AP), GPC1 (#16700-1-AP) from ProteinTech. pIRE1 and XBP1s antibodies were generated at Genentech. Secondary antibodies (rabbit #711-035-152 and mouse #715-035-150) were from Jackson ImmunoResearch Laboratories. mono-Rat IgG2b (#553986) from BD Pharmingen. Anti-hIRE1α for microscopy was made in-house at Genentech (GN35-18.ratIgG2b).
Validation	The antibodies used in this study were validated by the manufacturers and can be check using their respective catalog # on the following websites:<br>Cell Signaling: https://www.cellsignal.com/<br>Abcam: https://www.abcam.com/<br>ProteinTech: https://www.ptglab.com/<br>Jackson ImmunoResearch Laboratories: https://www.jacksonimmuno.com/<br>BD Pharmingen: https://www.bdbiosciences.com/en-us/products/reagents/flow-cytometry-reagents/research-reagents/flow-cytometry-controls-and-lysates/purified-rat-igg2b-isotype-control.553986<br>In-house pIRE1 and XBP1s antibodies, see Chang et al. j.molcel.2018.06.038 were tested by Western Blots, comparing WT and gene-specific knock-down by RNAi.<br>In-house human IRE1α antibody was previously reported in Harnoss et al., 2020, Cancer Research: IRE1α Disruption in Triple-Negative Breast Cancer Cooperates with Antiangiogenic Therapy by Reversing ER Stress Adaptation and Remodeling the Tumor Microenvironment.

Eukaryotic cell lines

Policy information about cell lines

Cell line source(s)	MDAMB231, HCC1806, KMS27, HCT116, AMO1, and U2OS cells were obtained from ATCC and mainained in an internal repository at Genentech.
Authentication	short tandem repeat (STR) profiles
Mycoplasma contamination	tested to ensure mycoplasma free within 3 months of use
Commonly misidentified lines (See ICLAC register)	none
